# Oxidation characteristic and thermal runaway of isoprene

**DOI:** 10.1186/s13065-023-01016-y

**Published:** 2023-09-02

**Authors:** Min Liang, Suyi Dai, Haijun Cheng, Chang Yu, Weiguang Li, Fang Lai, Kang Yang, Li Ma, Xiongmin Liu

**Affiliations:** School of Chemistry and Chemical Engineering, Guangxi University, Nanning, 530004 China

**Keywords:** Isoprene, Oxidation kinetic, Peroxide, Thermal runaway, Hazard

## Abstract

In this study, the oxidation characteristics of isoprene were investigated using a custom-designed mini closed pressure vessel test (MCPVT). The results show that isoprene is unstable and polymerization occurs under a nitrogen atmosphere. Under an oxygen atmosphere, the oxidation process of isoprene was divided into three stages: (1) isoprene reacts with oxygen to produce peroxide; (2) Peroxides produce free radicals through thermal decomposition; (3) Free radicals cause complex oxidation and thermal runaway reactions. The oxidation of isoprene conforms to the second-order reaction kinetics, and the activation energy was 86.88 kJ·mol^−1^. The thermal decomposition characteristics of the total oxidation product and purified peroxide mixture were determined by differential scanning calorimetry (DSC). The initial exothermic temperatures *T*_on_ were 371.17 K and 365.84 K, respectively. And the decomposition heat *Q*_DSC_ were 816.66 J·g^−1^ and 991.08 J·g^−1^, respectively. It indicates that high concentration of isoprene peroxide has a high risk of thermal runaway. The results of thermal runaway experiment showed that the temperature and pressure of isoprene oxidation were prone to rise rapidly, which indicates that the oxidation reaction was dangerous. The reaction products of isoprene were analyzed by gas chromatography-mass spectrometry (GC–MS). The main oxidation products were methyl vinyl ketone, methacrolein, 3-methylfuran, etc. The main thermal runaway products were dimethoxymethane, 2,3-pentanedione, naphthalene, etc. Based on the reaction products, the possible reaction pathway of isoprene was proposed.

**Supplementary Information:**

The online version contains supplementary material available at 10.1186/s13065-023-01016-y.

## Introduction

Isoprene (C_5_H_8_, 2-methyl-1,3-butadiene) is the basic unit of terpene compounds (including natural rubber and camphor) and an essential raw material of synthetic rubber [1]. It can be primarily used as a monomer that polymerizes with itself and various olefins to form polymers and copolymers with different properties. For example, poly(cis-1,4-isoprene), butyl rubber (isobutene-isoprene rubber, IIR), and thermoplastic elastic styrene–isoprene-styrene (SIS) rubber [2, 3]. Isoprene also accounts for many applications in the fine chemicals industry. It can be used as a platform chemical for the production of adhesives, pesticides, pharmaceuticals, oil additives, fragrances, and biofuels [4]. Among them, fragrances are one of the major products in the application of isoprene. Takabe et al.[5] reported that myrcene had been synthesized by the oligomerization of isoprene catalyzed by sodium/dialkylamine catalyst. Kudryavtsev et al.[6] generated geranyl chloride through the telomerization of isoprene and converted it into citral via the Sommelet reaction. Myrcene and citral are terpenes widely used in the everyday chemical and food industries and have special medical and medicinal value in the medical field [7–9]. As the research continues, the synthesis of isoprene is the main trend in the world today. It is seen that isoprene can be used as a promising chemical feedstock, which should attract our attention.

Isoprene is one of the critical volatile organic compounds (VOCs), the primary emission from terrestrial vegetation, second only to methane [10]. Due to its high reactivity, it has a significant impact on atmospheric chemistry. Isoprene plays an important role in the Earth's climate by lengthening the residence time of other gases, such as methane, which contribute to rising global temperatures [11, 12]. Isoprene also contributes to local pollution. It reacts with free radicals and ozone, the products of which contribute to the formation of secondary organic aerosol (SOA), ozone, and carbon monoxide. There have been many studies of the atmospheric chemistry of isoprene. The structure of isoprene contains conjugated double bonds and a methyl (-CH_3_) group, which are reactive groups and easily oxidized by the oxidants in the atmosphere, such as hydroxyl radicals (·OH), chlorine radicals (·Cl), ozone (O_3_), and nitrogen oxide free radicals (·NO_x_). H-atom abstraction and addition reactions are the primary reaction routes for isoprene and oxidants. The main removal route of isoprene was reacted with ·OH, up to ~ 85% of the total amount of isoprene reaction [13]. The ·OH radicals were mainly added to the double-bond unsaturated backbone of isoprene. Isoprene is asymmetric and multiple isomers can be generated. The initial addition of ·OH can occur on the four conjugated carbon chains of isoprene, forming multiple isoprene hydroxyl hydroperoxides (ISOPOOH) isomers [14–16]. These peroxide isomers are highly reactive and may enhance the complexity of the peroxidation reaction [17, 18]. ISOPOOH isomers can further react with nitric oxide (NO) or peroxy radicals (RO_2_·), leading to the formation of activated products, such as methyl vinyl ketone (MVK)[19], methacrolein (MACR)[20], isoprene hydroxy nitrate (IHN)[21, 22]. In the presence of high concentrations of nitric oxides (typically from industrial activities), it also reacts to generate tropospheric ozone [4]. In addition, ISOPOOH can be oxidized by ·OH to form isoprene epoxydiols (IEPOX) [17]. The main activities of IEPOX include vapor phase oxidation and particulate growth, which can contribute significantly to the formation of secondary organic aerosols (SOA). Statistics show that the increase in aerosols has a serious impact on human health, including increased mortality, cardiovascular and respiratory diseases and allergic diseases. Toxicological studies in vivo and in vitro have demonstrated the significant pulmonary toxicity of environmental aerosol particles [23]. As a result, there is a growing debate about the beneficial effects of isoprene in general, with questions about the benefits as a necessary basic feedstock versus the risks as an environmentally contaminated material.

Isoprene is highly volatile, flammable, and toxic, and its vapors can form explosive mixtures with air. Many industrial accidents involving isoprene have occurred worldwide [24]. For example, in 1974, there was an isoprene explosion accident occurred in the United States caused by a technical fault, resulting in multiple casualties. In 1983, an explosion of isoprene vapor from welding occurred in Japan. In 2003, a leak of isoprene at a Russia oil refinery resulted in 28 casualties and 45 poisonings. In 2011, an alumina container bag in Taiwan caught fire due to the release of unpurified isoprene from a heated alumina molecular sieve. Liu et al. [25] studied that the thermal polymerization and exothermic properties of isoprene mixed with pollutants were used to analyze by differential scanning calorimetry (DSC). The results showed that isoprene was unstable, the exothermic onset temperature was 473.15K, and the maximum decomposition heat was 1130J·g^−1^. In the process of storage, transportation and manufacturing, if isoprene is subjected external heat source, serious accidents such as combustion and explosion will occur. Moreover, oxygen is a highly reactive substance and tends to be inseparable from isoprene in production. The presence of oxygen increases the potential for an explosion accident. It is therefore necessary to investigate the oxidation reaction properties of isoprene. However, the existing research on the oxidation reaction of isoprene has focused on the field of atmospheric oxidation and less on its autoxidative reactivity. For safety in production, transport, and use, the thermal stability and oxidation reaction hazards of isoprene are essential for research.

This study investigated the oxidation characteristic and thermal runaway behavior of isoprene via a custom mini closed pressure vessel testing device (MCPVT). The thermal hazard of the reaction intermediates was evaluated by differential scanning calorimetry (DSC). Furthermore, the chemical compositions of the products were also analyzed by gas chromatography-mass spectrometry (GC–MS), and the reaction mechanism of isoprene was proposed based on the experimental results of thermal characteristics and reaction products. Understanding the thermal stability characteristics and thermal runaway of isoprene by means of thermal analysis is essential to prevent future accidents.

## Materials and experiments

### Materials

Isoprene (mass purity > 99.00%, molecular weight 68.11 g·mol^–1^) was obtained from Aladdin Industrial Corporation, China. The N_2_ and O_2_ gases (mass purity > 99.99%) were obtained from Guangxi Guoxin Gas Research Co., Ltd., China. KI (99.50%, Aladdin Industrial Corporation, China) and Na_2_S_2_O_3_ (99.95%, Aladdin Industrial Corporation, China) were used in the experiments.

### Thermal stability of isoprene by a mini closed pressure vessel test

**Fig. 1:**
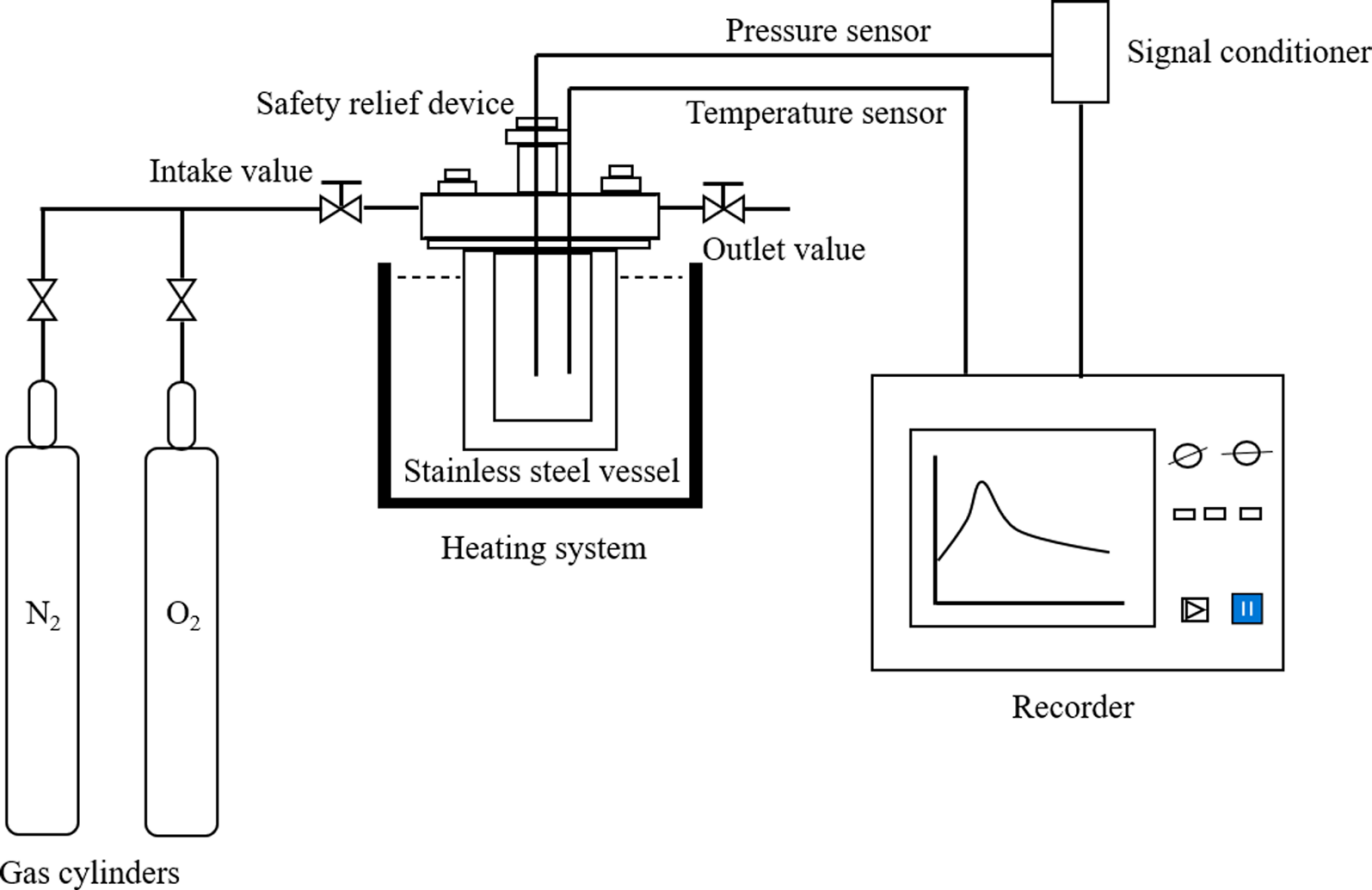
The experimental device of isoprene

A self-designed mini closed pressure vessel test (MCPVT) was employed to monitor the thermal reaction process of isoprene. MCPVT device consists primarily of five parts: (1) a mini closed pressure vessel with a safety relief device (made in China); (2) a pressure sensor, made by KYOWA Electronic Instruments Co. Ltd., Japan; the accuracy was 0.00001 MPa; (3) temperature sensor, made by HAKKO Development Co. Ltd., Japan; the accuracy was 0.001 K; (4) signal recorder, made by HIOKI Electric Co. Ltd., Japan; and (5) heating system, as shown in Fig. 1. The rate of change in pressure and temperature over time was then obtained to explore the severity of the material reaction.

A glass tube (volume: 5 mL) containing approximately 0.21 g isoprene sample was placed in a reaction vessel. Oxygen or nitrogen was injected into the reactor at an initial pressure of 0.31 MPa. The heating system was turned on and a signal recorder immediately monitored temperature and pressure changes. The experiment was performed to trace the possible thermal runaway and the explosion of isoprene in the condition of program raising temperature. Experiments at each reaction temperature were conducted in duplicate to ensure reproducibility and to determine the experimental errors.

### Iodimetry analysis of isoprene peroxide

Organic peroxide contains a -O–O- bond structure that is chemically reactive and unstable. There are many methods for detecting organic peroxides. The most commonly used method is iodimetry. Iodimetry was used to measure the peroxide concentration at different temperatures to determine the formation of isoprene peroxide. In principle, the potassium iodide aqueous solution was oxidized by the strong oxidant peroxide to form free iodine, which is coloured using starch as an indicator (Eq. 1). Subsequently, the solution was titrated with sodium thiosulfate standard solution, and the free iodine was reduced to iodide ions (Eq. 2). The complete discolouration of the solution from blue to colourless is considered as the endpoint of the reaction. Briefly, 0.2 g of the sample was dissolved in a chloroform solution. Then, 5 mL of potassium iodide solution and a new 1% starch solution were added. Peroxide values were calculated from the iodine release from potassium iodide. The peroxide value was expressed as milligrams per kilogram (mmol·kg^−1^).

1 $$ {\text{2KI}} + {\text{ ROOH }} + {\text{ H}}_{{2}} {\text{O }} \to {\text{ I}}_{{2}} + {\text{ 2KOH }} + {\text{ ROH}} $$

2 $$ {\text{I}}_{{2}} + {\text{ 2Na}}_{{2}} {\text{S}}_{{2}} {\text{O}}_{{3}} \to {\text{ Na}}_{{2}} {\text{S}}_{{4}} {\text{O}}_{{6}} + {\text{ 2NaI}} $$

### Thin layer chromatography analysis

The formation of isoprene peroxide was monitored by thin-layer chromatography (TLC). Briefly, the oxidation products of isoprene oxidized at different temperatures (323.15K, 328.15K, 333.15K, 338.15K, and 343.15K) for 8 h were prepared by MCPVT. The products were analyzed using a TLC plate performed on a Merck TLC-plate aluminum silica gel 60 F_254_. The developing solvent was a mixture of petroleum ether/ethyl acetate (1:1, v/v). After carefully drying and completely removing the solvent, the TLC plates were immersed in a solution of potassium iodide-containing starch. Due to the strong oxidation properties of peroxides, blue spots appear on the TLC silicone plates.

### Differential scanning calorimeter analysis

Differential scanning calorimetry (DSC) is a technology to study the relationship between the physical quantity of a substance (heat of reaction and enthalpy of reaction) and temperature change, as well as the relationship between power difference (heat flow rate) of a substance and reference substance and temperature change under programmed temperature control [26]. The thermal decomposition properties of isoprene peroxide were measured using DSC. The analysis steps were as follows. The peroxide was accurately weighed into a stainless-steel pressure dish. It was manually sealed and placed on a sample holder in the DSC furnace. The other holder placed the sample tray in the air as a blank reference. Under a nitrogen atmosphere (35.0 mL·min^−1^), the initial equilibrium temperature was 303.15K, then the temperature rose to 473.15K at 10 K·min^−1^. The thermal curves were analyzed using STARe software [27]. The instrument detection sensitivity was 0.04 μW.

### Thermal runaway of isoprene

Thermal runaway experiments with isoprene were conducted in MCPVT at programmed temperatures. Temperatures were set from 298.15 K to 373.15 K. The mass of isoprene is 0.21 g, and different relative molar ratios of isoprene/oxygen (1:1.3, 1:1.6, and 1:2.0) were added to the reactor. Contrast tests were performed for different amounts of isoprene. The amounts of isoprene and oxygen were 0.42 g and 0.62 MPa (Rev. molar ratio = 1:1).

### Gas chromatography–mass spectrometry analysis

The reaction products of isoprene were qualitatively analyzed by gas chromatography-mass spectrometry (GC–MS, GC/MS-QP2010 Ultra, SHIMADZU, Japan). GC–MS analyses were performed on a GC-2010 plus gas chromatograph coupled with a Rxi-5SilMS fused silica capillary column (30 m × 0.25 mm × 0.25 μm, J&W Scientific Inc.). The injection volume was 1 μL at a carrier gas flow of 4 mL·min^−1^ helium with a split ratio of 1:30. The initial oven temperature of 333.15 K was maintained for 1 min and then raised to 373.15K at 3K·min^−1^ and kept for 3 min. The other conditions were set: interface temperature 473.15 K, ion source temperature 493.15 K, and electron impact ionization (EI) 70 eV. Mass spectra were analyzed in the range of 20–650 atom mass units (AMU) at a rate of 90 scans·min^−1^ for a run time of 15 min. The detected peaks were identified based on a mass spectrometer's National Institute of Standards and Technology 2011 library.

## Results and discussion

### Pressure behavior of isoprene under a nitrogen atmosphere

A self-designed MCPVT device was used to assess the thermal stability of isoprene under a nitrogen atmosphere and raising temperature conditions. The temperature condition was set from 303.15 K to 443.15 K.

**Fig. 2:**
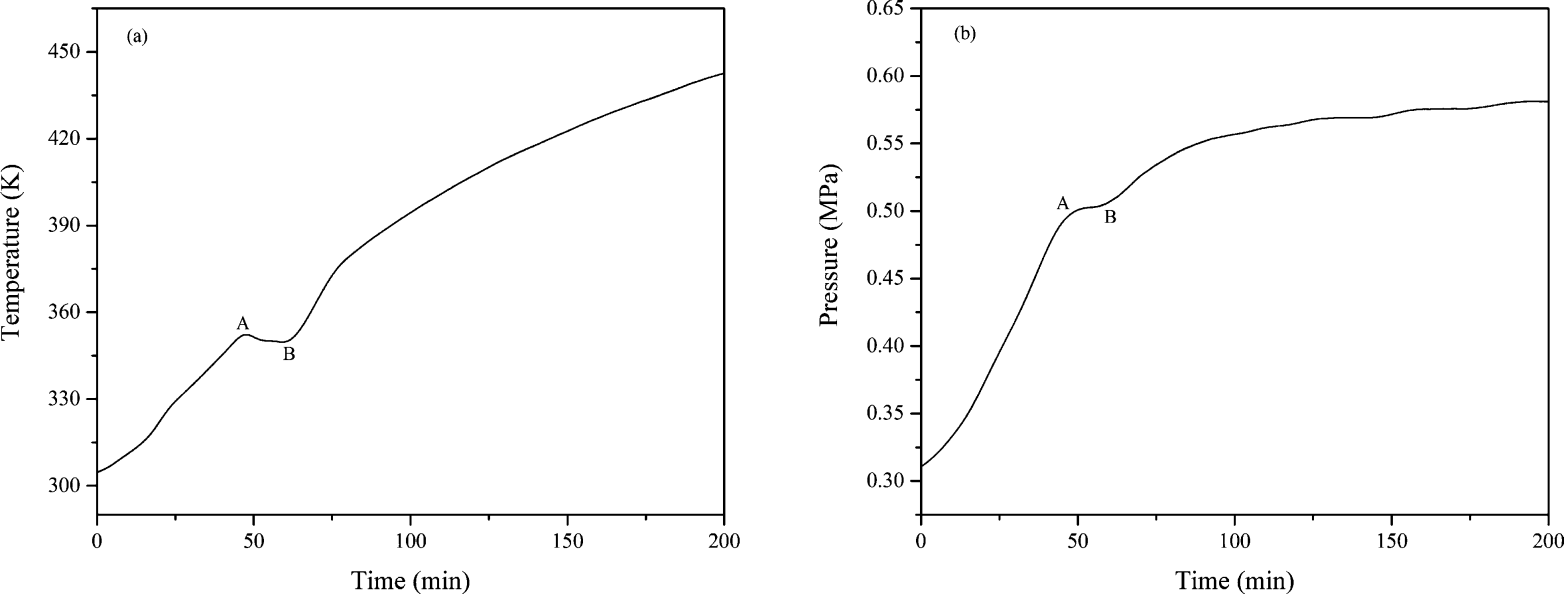
The temperature and pressure behavior of isoprene under a nitrogen atmosphere, **a** T-t; **b** P–t

The results are shown in Fig. 2. The temperature vs. time (T-t) curve under a nitrogen atmosphere shows that the temperature increased with time (Fig. 2a), and there was a significant endothermic peak when the temperature reached approximately 352.40 K (**Point A**). Then, the temperature drops to a minimum value of 349.65 K (**Point B**) and rises again because of the constant heat from the heating system. Similar results are shown in the pressure vs. time (P–t) curve (Fig. 2b). The pressure increased with time and reached a plateau when the temperature reached at **Point A**. Since the external heat source maintained constant heating, the pressure rose again after **Point B**.

According to the Antoine equation [28, 29], the boiling point of isoprene is 344.15 K when the vapor pressure is 0.31 MPa. This temperature value was similar to the temperature at **Point A** in Fig. 2a. It was inferred that the temperature at **Point A** is the boiling point of isoprene at high pressure. Near the boiling point, significant vaporization of the liquid occurs. Since the liquid vaporization process is a heat-absorbing, it leads to a temperature drop in the confined system.

**Fig. 3:**
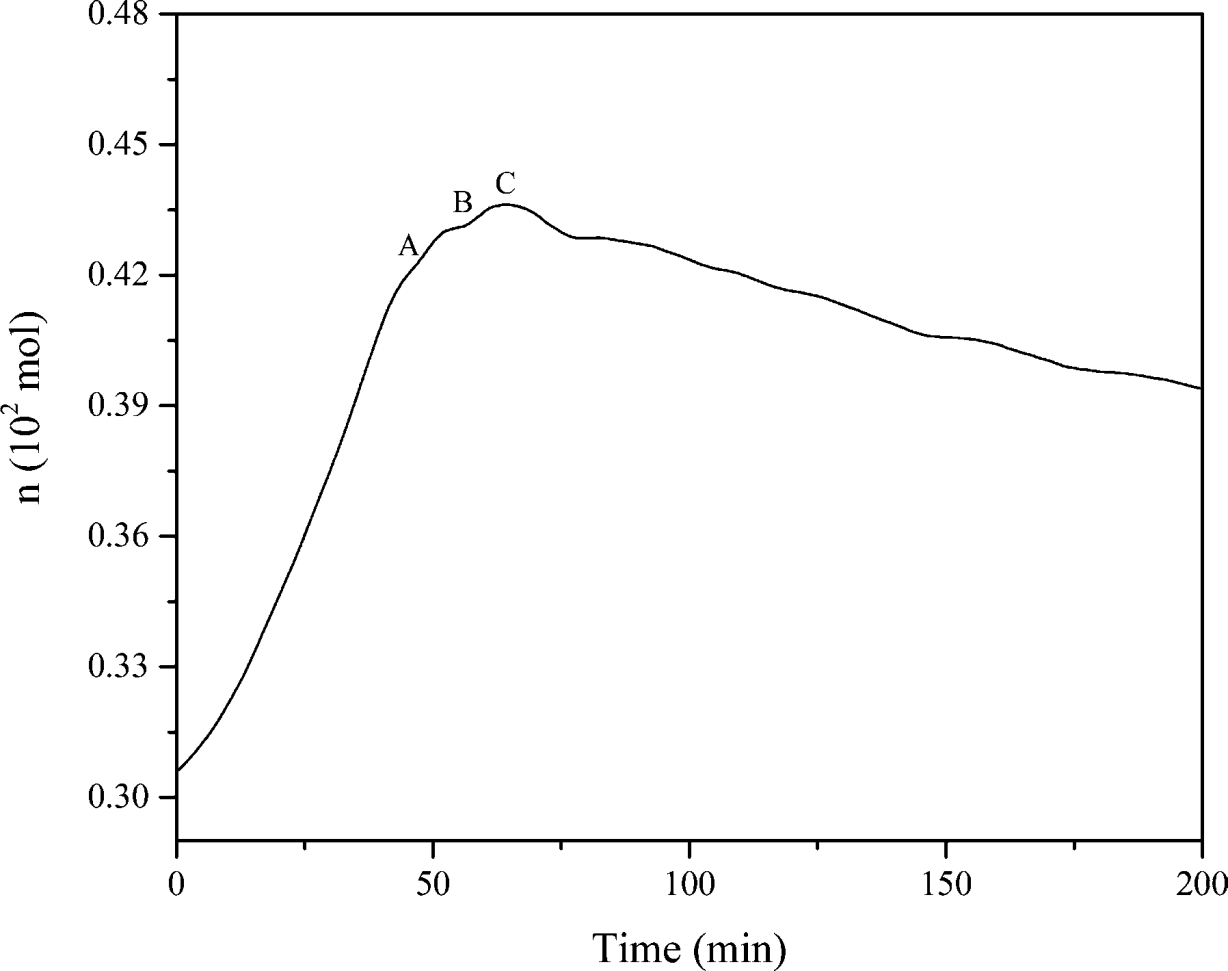
Plots of n-t of isoprene under a nitrogen atmosphere

Assuming that both isoprene and nitrogen are ideal gases, the amount of gas can be treated quantitatively according to the ideal gas equation of state (PV = nRT, where P is the pressure, V is the reactor volume, n is the moles of gas in the reactor, R is the ideal gas constant, and T is the temperature). The variation of the amount of gaseous substance with time (n-t) in the reaction process is shown in Fig. 3, and the n-t curve displays an increasing line at the beginning period of the reaction. After that, the gas reached its maximum at 352.28K (**Point C**) and then decreased. It was shown that a chemical reaction of isoprene began, which consumed the amount of isoprene in the reaction kettle, reducing the total amount of the gas. Therefore, experiments show that isoprene appears to s vaporize significantly and undergoes chemical reactions at 303.15–443.15K under a nitrogen atmosphere.

### Thermal oxidation of isoprene under an oxygen atmosphere

The pressure and temperature behavior under an oxygen atmosphere were monitored to determine the thermal stability of isoprene. Experiments were carried out at 300.15 K to 375.15 K under an oxygen atmosphere on thermal analysis.

**Fig. 4:**
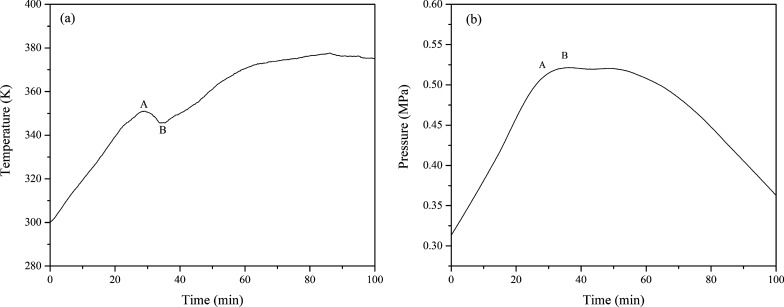
Plots of *P* and *T* vs. reaction time t of isoprene oxidation

**Fig. 5:**
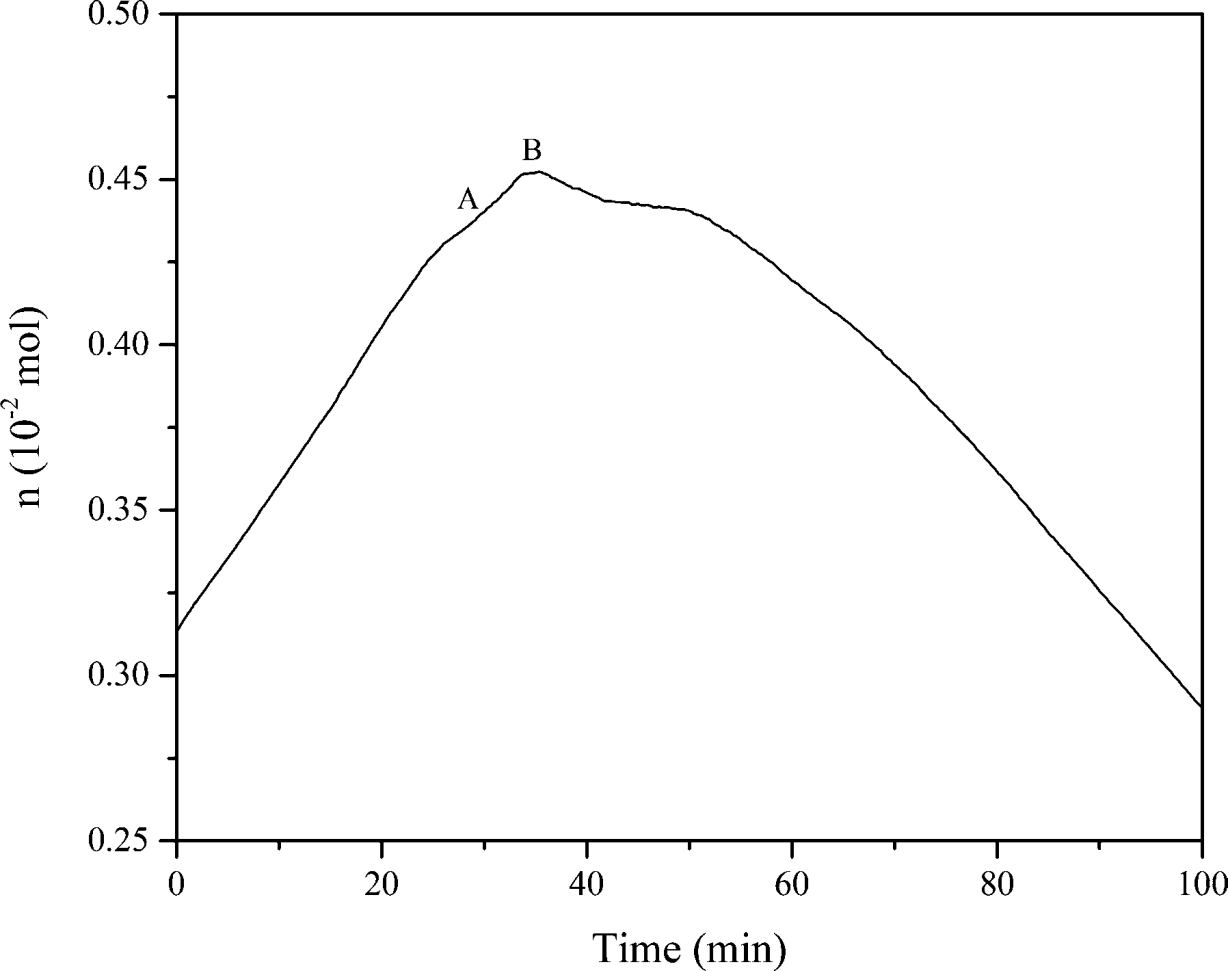
The curve of n-t of isoprene oxidation

The temperature vs time (T-t) and pressure vs time (P–t) curves are shown in Fig. 4. The T-t curve shows that the temperature increased with time and exhibited an endothermic peak at a temperature of 351.03 K. Simultaneously, the pressure shows a downward trend. According to the ideal gas equation of state, the gas moles vs time (n-t) curve of isoprene is shown in Fig. 5. The n-t curve exhibited a significant decrease at approximately 35 min, indicating that many reactants have been consumed, and it can be inferred that a chemical reaction occurred.

**Fig. 6:**
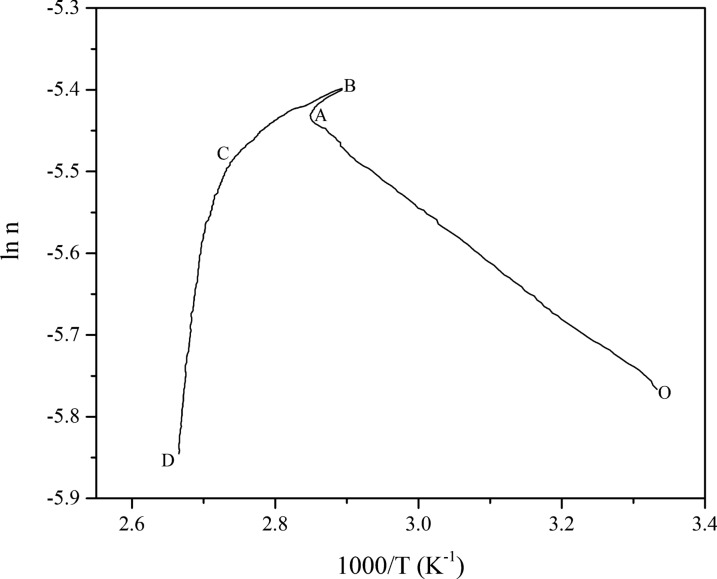
Plots of ln n vs. 1000/T of isoprene oxidation

In addition, the natural logarithm of the amount of gas as a function of 1000/T was plotted in Fig. 6. The curve is divided into four segments. (1) Segment OA: The gas moles increased with increasing temperature, indicating the volatilization of isoprene; (2) Segment AB: Gasification of isoprene was an endothermic process, and the gas moles increased; (3) Segment BC: The oxygen consumption process occurred at 345.65 K after gasification, indicating the significant initial oxidation reaction of isoprene. Isoprene reacted with oxygen to give rise to hydrogen peroxide as the initial product; (4) Segment CD: When the temperature reached 362.53 K, the pressure and gas moles dropped rapidly. It can be inferred that the rapid oxidation reaction lowered the pressure. Hence, studying the oxidation behavior of isoprene in MCPVT can provide detailed insight into how temperature affects its thermal stability and autoxidation properties in closed conditions.

### Oxidation kinetic of isoprene

There are two parallel reactions of isoprene in an oxygen atmosphere, including dimerization and oxygenation. Dimerization of isoprene via the Diels–Alder reaction yields a mixture of cyclic monoterpenes, which is a second-order reaction [30, 31]. A relatively simple reaction rate model can approximate the dimerization and oxidation reactions of isoprene. The kinetic model for the isoprene reaction considers the reaction rate constants for the dimerization and oxidation reactions to predict the reactant and product molarities as a function of time. Correspondingly, the general reaction responsible for the dimerization and oxidation of isoprene is stated as follows:

$$2isoprene \stackrel{ {k}_{d} }{\to } products$$

$$isoprene + {O}_{2}\stackrel{{ k}_{o} }{\to } products$$

The reaction rate of isoprene is given by the following equation:

3 $$-\frac{d{n}_{A}}{dt}={k}_{d}{\left({n}_{A}-x\right)}^{2}+{k}_{o}{\left({n}_{A}-x\right)}^{\alpha }{({n}_{B}-x)}^{\beta }$$

where *k*_d_ and *k*_o_ are the apparent rate constants for dimerization and oxidation, *n*_A_ and *n*_B_ are the initial mole of isoprene and oxygen at time *t*, respectively; *α* and *β* are the reaction order of isoprene and oxygen, respectively.

Oxygen is highly reactive, so in contrast to dimerization of two isoprene molecules, direct isoprene oxidation with oxygen should be the dominant reaction mechanism. At high oxygen pressures, the isoprene dimerization can be neglected. Furthermore, for the present evaluation of the experimental data, the reaction rate constant can be calculated by assuming that the oxidation reaction is a second order reaction (first order relative to the isoprene and first order relative to oxygen). That is, α = β = 1. Then, rearranging Eq. (3), we have

4 $$-\frac{d{n}_{A}}{dt}={k}_{o}\left({n}_{A}-x\right)({n}_{B}-x)$$

In this study, two kinetic models are discussed in depth to analyze the kinetic parameters of isoprene oxidation.

Model 1: Because of its relatively low boiling point, it is assumed that isoprene can be completely dispersed from the liquid phase into the gas phase at the experimental temperature. For convenience, oxygen is considered an ideal gas, and the molar number can be calculated using the ideal gas equation (*n* = *PV*/*RT*). The initial addition of isoprene is assumed to be equal to that of oxygen, that is *n*_A_ = *n*_B_.

Simplified Eq. (4)

5 $$-\frac{d{n}_{A}}{dt}={k}_{o}{\left({n}_{A}-x\right)}^{2}$$

Integrating Eq. (3)

6 $$\frac{1}{{n}_{A}-x}={k}_{o}t+C$$

where *C* is a constant.

Model 2: According to the Antoine equation, the isoprene is not sufficiently vaporized at the experimental temperature. The initial amounts of isoprene and oxygen are unequal, i.e., *n*_A_ ≠ *n*_B_.

Then, integrating Eq. (4)

7 $$\frac{1}{{n}_{A}-{n}_{B}}\mathrm{ln}\frac{{n}_{A}-x}{{n}_{B}-x}={k}_{o}t+C$$

In this way, the calculated equations for each model can be reduced to linear equations. For Eqs. (6) and (7), the left side of each equation is a *y* variable, and *t* is an *x* variable. There is a linear relationship between the *y* and *x* variables with a slope equal to the apparent rate constant *k*.

In the study of the effect of temperature on the rate constant of a chemical reaction, it is well known that the rate constant of the reaction grows exponentially with temperature. This is demonstrated in the following classical Arrhenius equation.

8 $$k=A{e}^{-{E}_{a}/RT}$$

where *k* is the rate constant of the reaction at temperature *T*, *A* is the frequency factor, *E*_a_ is the activation energy of the reaction, and *R* is the gas constant.

Logarithm Eq. (8)

9 $$lnk=-\frac{{E}_{a}}{R}(\frac{1}{T})+lnA$$

The plot of ln *k* vs 1/*T* is expected to be straight, provided the reaction order is chosen correctly. The Arrhenius kinetic parameters, *E*_a_ and *A*, can be calculated accordingly from the plots.

**Fig. 7:**
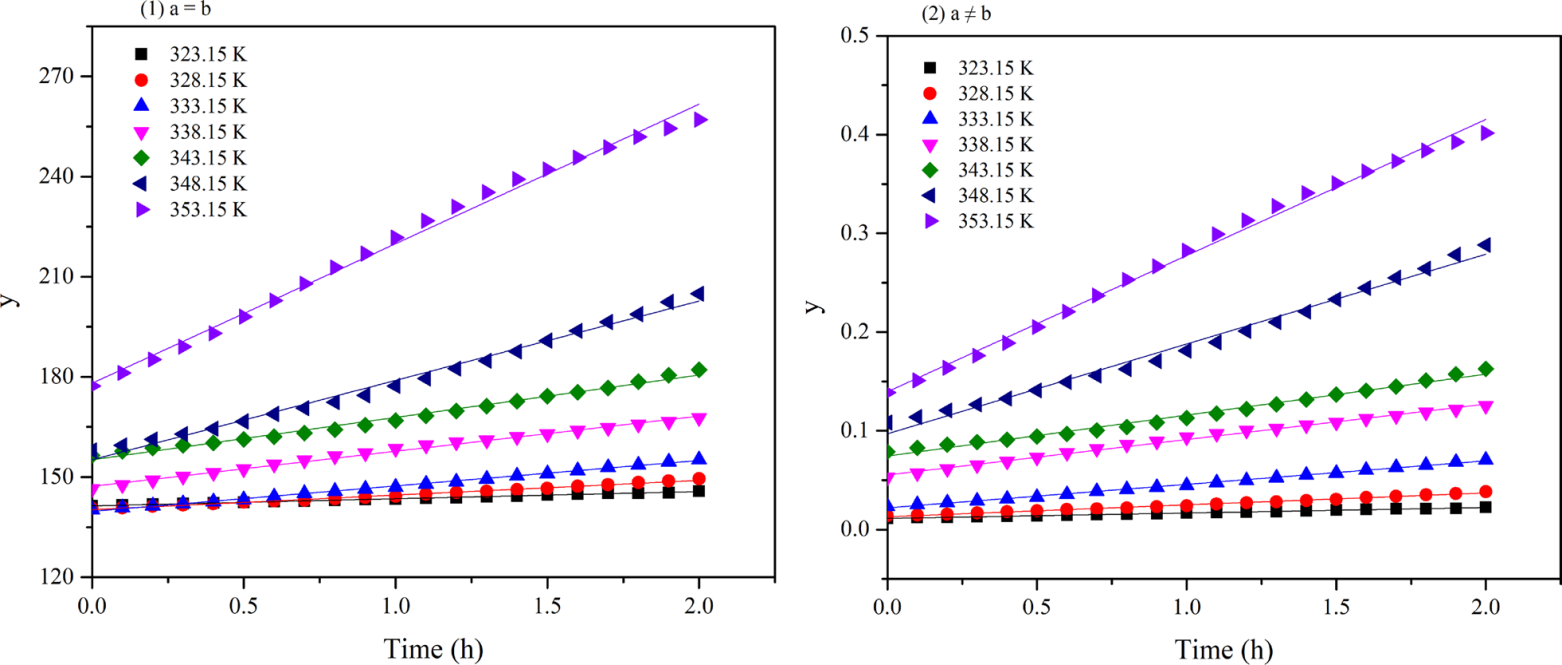
The plot of molar number versus time for the two models

The isoprene reaction kinetics were investigated at seven temperatures (323.15 K, 328.15 K, 333.15 K, 338.15 K, 343.15 K, 348.15 K, and 353.15 K). The kinetic calculations began with the decrease of molar number, which was recorded as 0 h. The rate constants of the two models were shown in Fig. 7 and Table 1. It was already evident that the apparent rate constant, *k*, increased with temperature.

**Table 1:** Kinetic parameters for oxidation of isoprene

T/K	a = b		a ≠ b	
	*k* (mol·h^−1^)	R^2^	*k* (mol·h^−1^)	R^2^
323.15	2.14 ± 0.027	0.9969	0.0055 ± 7.01E−5	0.9969
328.15	4.39 ± 0.084	0.9932	0.012 ± 2.32E−4	0.9931
333.15	7.63 ± 0.088	0.9975	0.024 ± 2.82E−4	0.9974
338.15	10.53 ± 0.17	0.9949	0.036 ± 5.72E−4	0.9952
343.15	12.69 ± 0.32	0.9882	0.041 ± 0.0011	0.9876
348.15	23.77 ± 0.55	0.9901	0.091 ± 0.0022	0.9886
353.15	41.81 ± 0.75	0.9940	0.14 ± 0.0022	0.9952

**Fig. 8:**
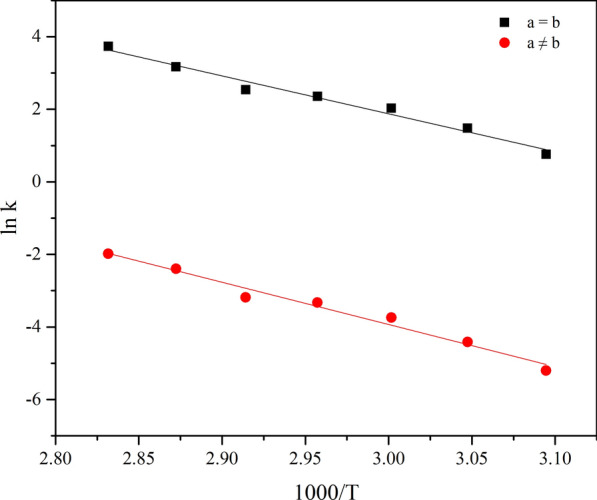
Plots of ln k vs 1000/T

The plot of ln *k* vs 1000/*T* was presented in Fig. 8. From the regression line, the linear equations were ln*k* = − 10.45 × 1000/*T* + 33.22 (R^2^ = 0.9796) and ln *k* = − 11.64 × 1000/*T* + 30.99 (R^2^ = 0.9796), respectively. The apparent activation energies for the two models are calculated to be 86.88 kJ·mol^−1^ and 96.79 kJ·mol^−1^, respectively. By comparing the correlation coefficient of the rate constant with the standard deviation of the activation energy, the isoprene oxidation reaction was more closely consistent with kinetic Model 1. That is, isoprene was completely vaporized before the reaction began, and the initial concentrations of isoprene and oxygen were the same. Therefore, the activation energy of isoprene oxidation reaction was 86.88 kJ·mol^−1^.

This result differs from previous reports. Pedersen et al. [32] studied the rate constants and activation energies of ozone reaction with isoprene in an aqueous solution using the stopped-flow technique. The result showed that the rate constants at 298 K and the activation energies were 4.1 (± 0.2) × 10^5^ M^−1^·s^−1^ and 19.9 (± 0.5) kJ·mol^−1^. Khamaganov et al. [33] investigated that the rate constants for the gas-phase ozone reaction with isoprene were measured using the relative rate technique over the temperature range 242–363 K and at 760 torr total pressure. The Arrhenius expression was determined: *k* = (10.9–2.17 + 2.70) × 10^–15^ exp[-(1998 ± 63)/*T*] (in units of cm^3^·mol^−1^·s^−1^) for the reactions. Chuong et al. [34] reported that the rate constants for the OH + isoprene reactions had been measured in He and over the temperature range 300 ~ 423 K using a discharge-flow system coupled to laser-induced fluorescence. An Arrhenius expression of *k*_0_ = (9.3 ± 5.4) × 10^–29^ exp[(1560 ± 230)/*T*] cm^6^·mol^−2^·s^−1^ was obtained from a weighted linear least-squares fit of the *k*_0_ data vs. temperature. It can be concluded that the kinetic parameters were different due to the different reaction process and determination methods.

### Peroxide formation and thermal decomposition

**Fig. 9:**
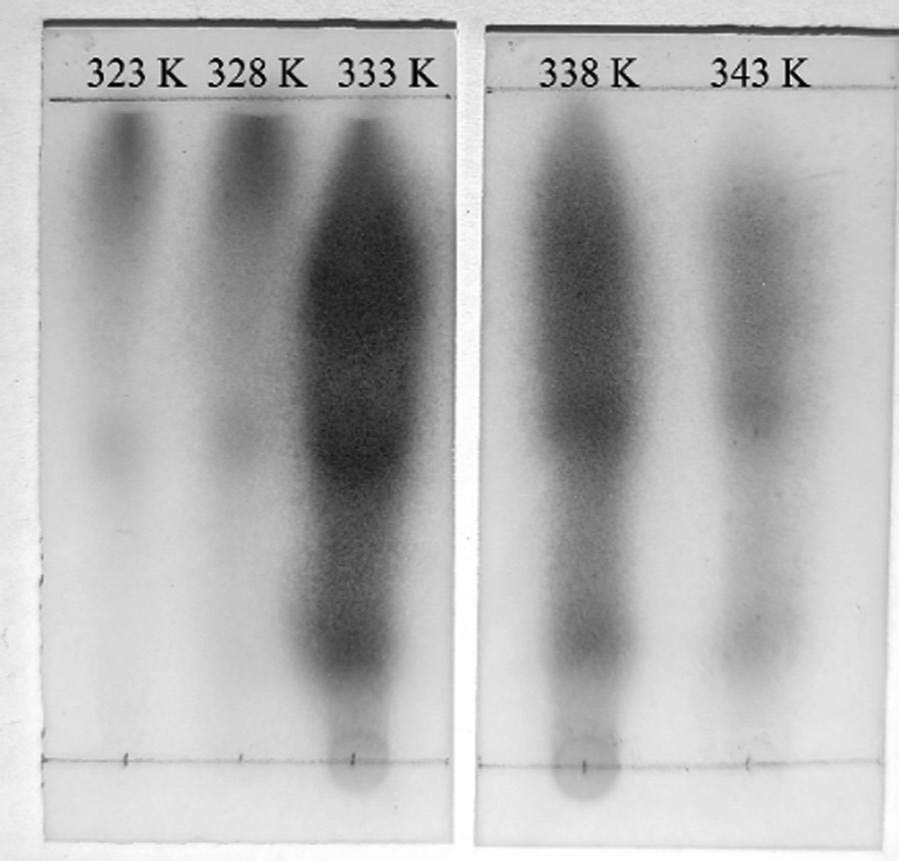
The TLC analysis of isoprene peroxides under different temperatures

In order to determine the formation of peroxide during the reaction of isoprene and oxygen, the thin layer chromatography (TLC) method was used to analyze the reaction products, as depicted in Fig. 9. The results of the TLC analysis have demonstrated the formation of the complex products for each reaction that could not be easily isolated by simple filtration. The polarities are similar, resulting in the appearance of continuous bands on the TLC plate. As the temperature was increased, the species of peroxides first increased and then decreased, reaching a maximum value at 333 K. This result further demonstrated that the reaction of isoprene with oxygen first formed peroxides, and that the subsequent deep oxidation was induced by pyrolysis of the peroxides.

**Fig. 10:**
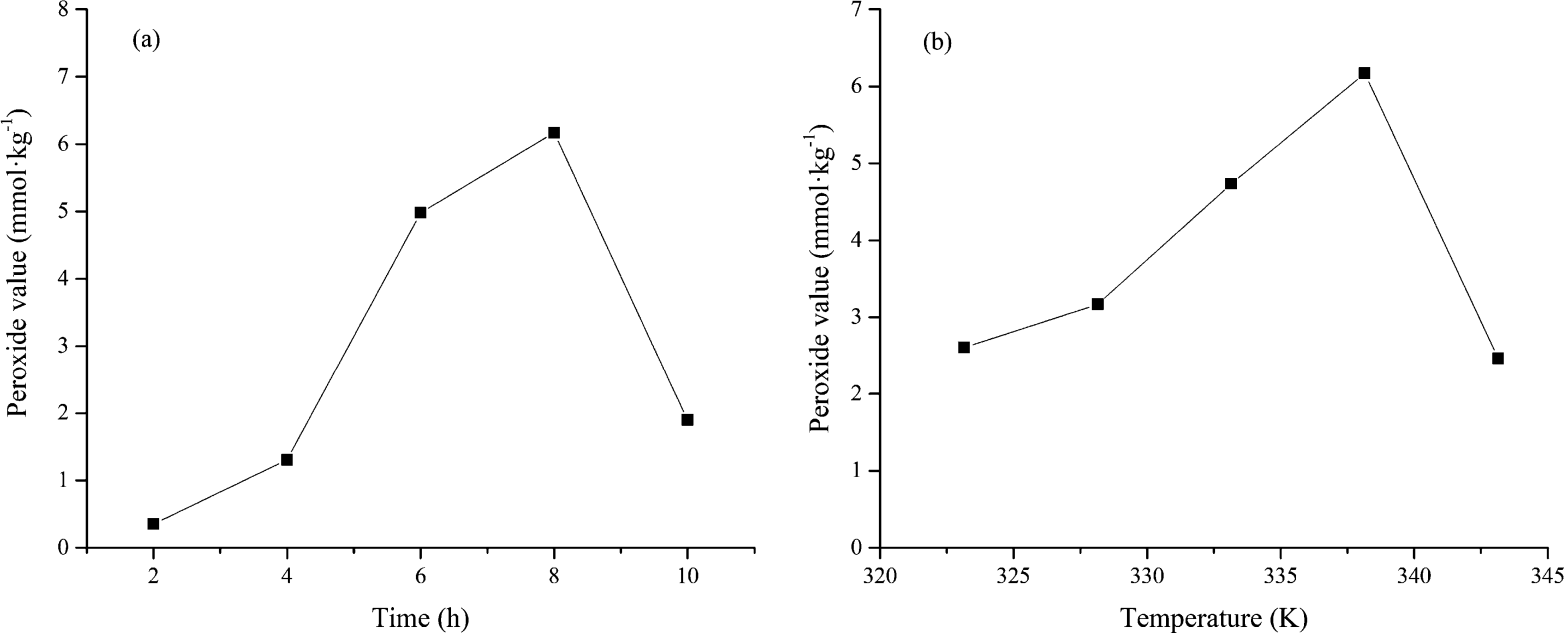
The effect of reaction conditions on peroxide value, **a** Reaction time; **b** temperature

Further investigations were performed to study the effect of the main reaction parameters (reaction time and temperature) on the peroxide value. The peroxide values of the reaction products were analyzed by iodimetry. As shown in Fig. 10, the iodimetry results showed that isoprene yielded different peroxide contents for different oxidation processes. Changes in the peroxide value with increasing reaction time at 338.15 K are shown in Fig. 10a. The initial reaction time of 2 h gave a low peroxide value of 0.36 mmol·kg^−1^. When it reacted for 8 h, the peroxide value was markedly enhanced to 6.17 mmol·kg^−1^. However, when the time was further extended to 10 h, the peroxide value decreased obviously. A similar trend is observed between the peroxide values and the temperature. The relationship is shown in Fig. 10b. The peroxide value increased continuously and reached a maximum of 6.17 mmol·kg^−1^ at 338.15K, indicating that more peroxides were produced. The peroxide value rapidly decreased when the temperature was further increased to 343.15K.

The main reason for this reduction can be explained as follows. In the initial reaction (10) stage, the initial step of the reaction of isoprene with oxygen is mainly peroxide formation, and the peroxide concentration increases with time. However, peroxides are unstable and prone to thermal decomposition at high temperatures [35]. Free radicals are active and can trigger many reactions to form complex products. Due to the interaction of reactions (10), (11), and (12), the peroxide concentration will reach the maximum value of 6.17 mmol·kg^−1^ (at 8 h and 338.15K). The peroxide concentration cannot maintain a stable maximum at a specific temperature. In order to reduce the thermal decomposition of the peroxide, the reaction temperature can be reduced.

10 $$\text{Oxidation: Isoprene + }{\text{O}}_{2}\stackrel \, {\to }{\text{Peroxides}}$$

11 $$\text{Decomposition: Peroxides}\stackrel \, {\to }\text{Free radical}$$

12 $$\text{Complex reaction: Peroxides (or isoprene) + Free radical}\stackrel \, {\to }\text{Complex products}$$

**Fig. 11:**
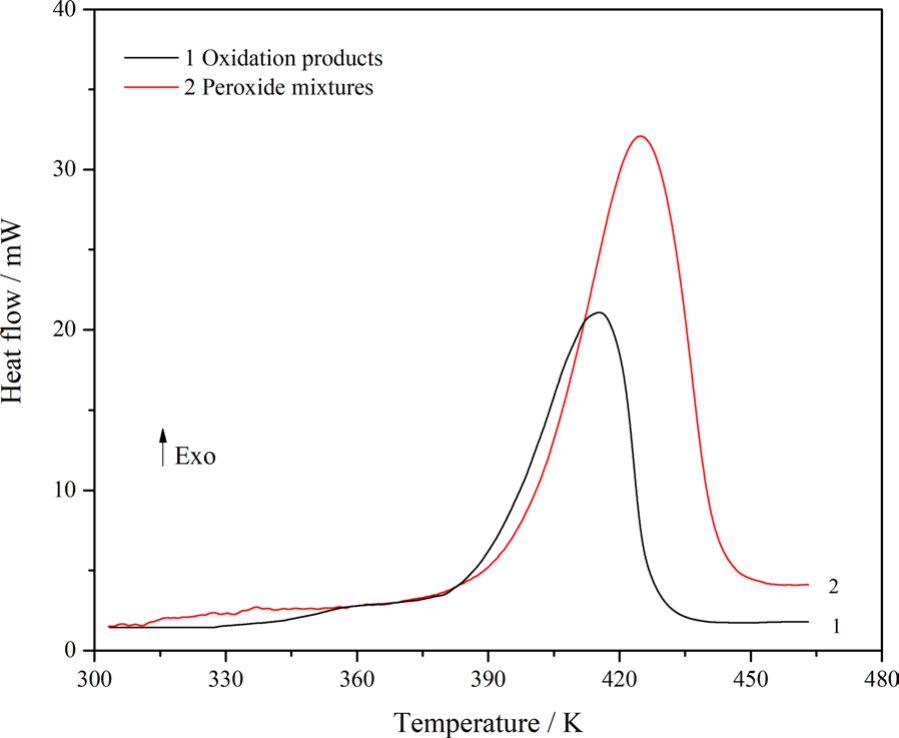
Heat flow vs. temperature of isoprene oxidation products

The thermal stability of the oxidation products of isoprene was measured by DSC. A thorough observation of the exothermic behaviour in nitrogen gas was performed in the temperature range of 303.15 to 463.15 K, and the results are shown in Fig. 11.

There are two curves representing the total oxidation products after the reaction finished (**Curve 1**) and for the peroxide mixtures purified by column chromatography (**Curve 2**). The DSC curves exhibited narrow and sharp exothermic peaks. The characteristic parameters are listed in Table 2, including the onset temperature (*T*_on_), accelerating exothermic temperature (*T*_a_), the maximum heat release (*T*_M_), the exothermic offset temperature (*T*_off_), and the heat of decomposition. The heats of decomposition (*Q*_DSC_) for the total oxidation products and purified peroxide mixtures were 816.33 J·g^−1^ and 991.08 J·g^−1^, almost four times larger than the standard for hazardous substances (250 J·g^−1^) [36]. The heat of decomposition for the purified peroxide mixtures is higher than that for the total oxidation products, indicating that the peroxides dominated the exothermic decomposition process. The abundant heat released by thermal decomposition reactions cannot be sufficiently removed, leading to heat accumulation and an enhanced possibility of thermal runaway.

**Table 2:** The characteristic safety parameters of isoprene peroxide

Curve	Sample (mg)	*T*_on_ (K)	*T*_a_ (K)	*T*_M_ (K)	*T*_off_ (K)	*T*_*d*_ (K)	*r*_d_ (mW·s^−1^)	*Q*_DSC_ (J·g^−1^)
1	3.49	371.17	387.66	415.61	439.21	404.19	0.15	816.33
2	4.96	365.84	394.38	424.97	442.70	414.45	0.22	991.08

**Fig. 12:**
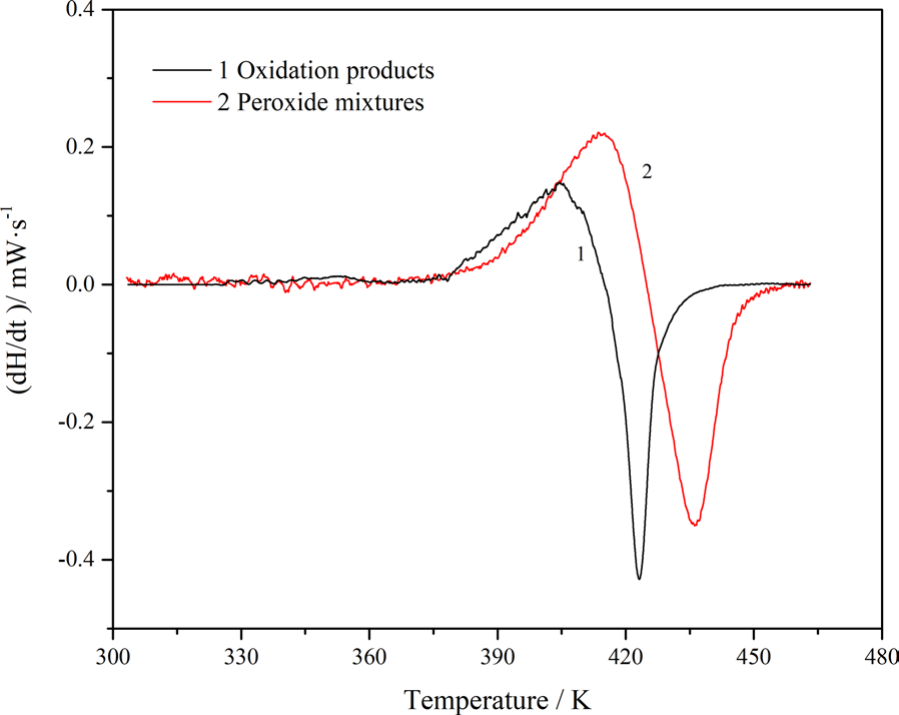
First derivative of heat flow as a function of temperature

In addition, the first derivative of the heat flow is plotted as a function of temperature, as depicted in Fig. 12. The heat release rate (*r*_d_) and the corresponding maximum temperature (*T*_d_) are listed in Table 2. It can be inferred that the faster the heat is released, the easier it is for the reaction to get out of control. Furthermore, high concentrations of isoprene peroxide present a high risk of thermal runaway. It was indicated that the isoprene peroxides are dangerous. Therefore, for the safety of production, storage, transportation, and usage, oxidation of isoprene should be avoided to generate peroxides with potential thermal hazards.

### Thermal runaway of isoprene

**Fig. 13:**
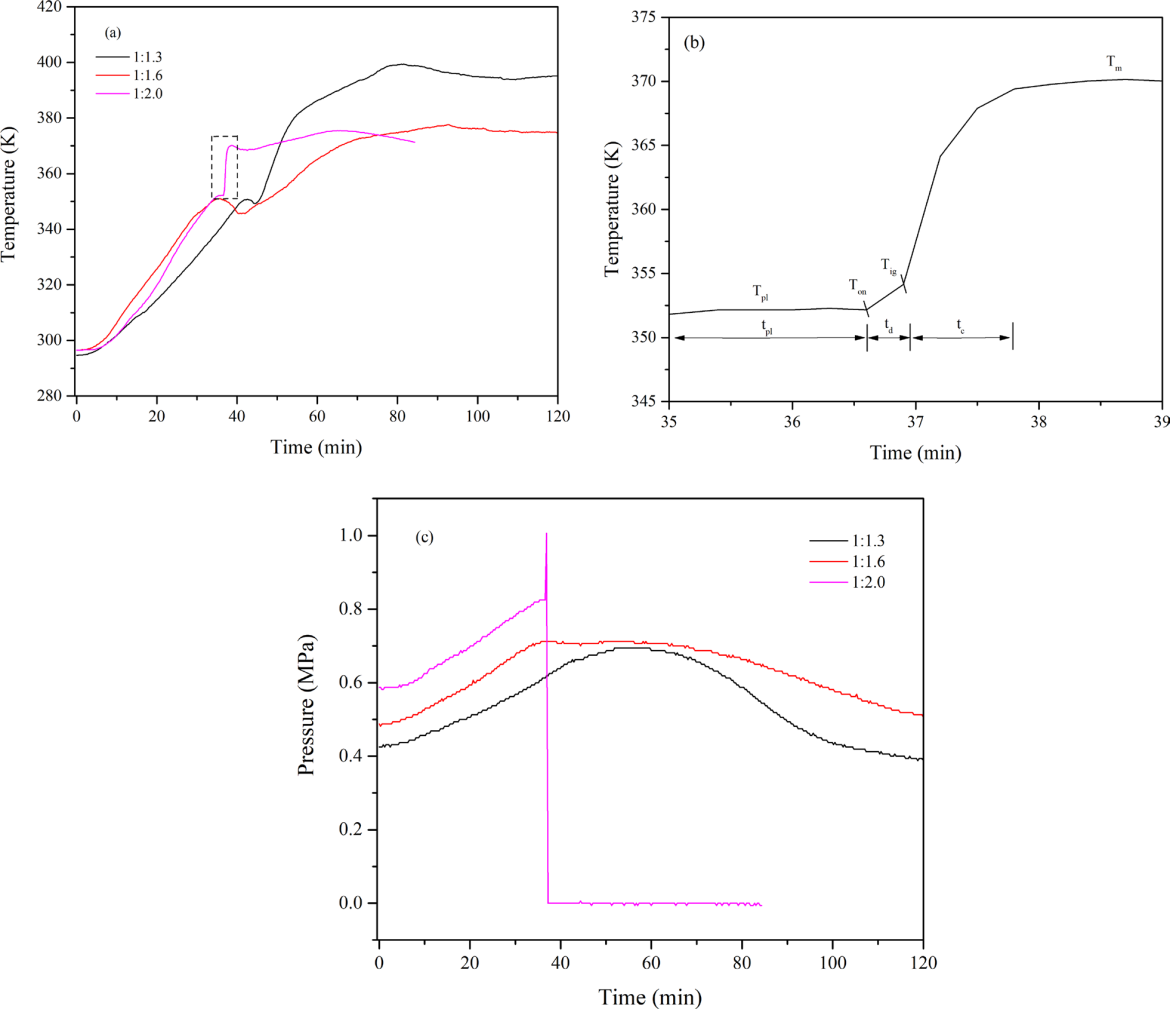
Thermal runaway for different molar ratios of isoprene, **a** *T*-*t*; **b** The expansion of near-ignition region of 1:2.0; **c** *P*–*t*

**Fig. 14:**
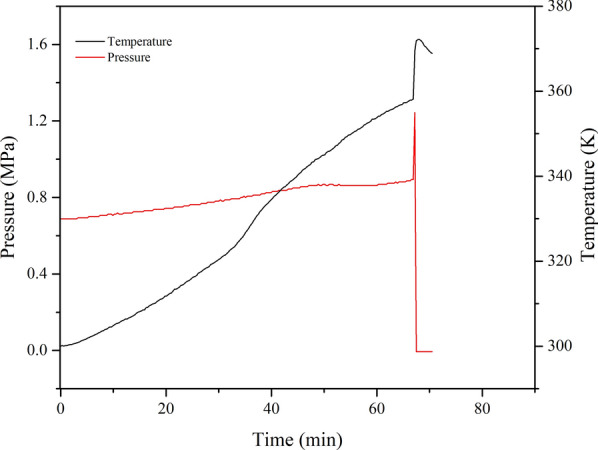
Thermal runaway with the isoprene amount of 0.42 g

The thermal runaway behaviour of isoprene in MCPVT is shown in Fig. 13. As shown in Fig. 13a, there were no significant thermal runaway behaviours in the C_5_H_8_/O_2_ ratio of 1:1.3 and 1:1.6. In contrast, a thermal runaway phenomenon occurred in the isoprene reaction process when the ratio of C_5_H_8_/O_2_ was 1:2.0. The *T*-*t* curve indicates a violent exothermic reaction at temperatures up to approximately 352.15 K. The system temperature rapidly increased from 354.15 K to 370.15 K with a rate of 16.2 K·min^−1^, accompanied by a sharp increase in system pressure (Fig. 13b). Large amounts of peroxide intermediates were formed from the oxidation of isoprene. *Peroxides* are compounds containing the -O-O- group that is unstable and prone to cleavage at high temperatures, releasing large amounts of heat [37]. Similar results occurred in the C_5_H_8_/O_2_ ratio of 1:1 (0.42 g isoprene and 0.62 MPa oxygen), as shown in Fig. 14. It has been shown that thermal runaway reactions are prone to occur as the amount of reactants increases.

According to the obtained *T*-*t* curve of 1:2.0, more detailed thermal runaway characteristic parameters of isoprene can be discussed (Fig. 13). The plateau temperature (*T*_pl_) is observed at 352.15 K before ignition, which is consistent with the gasification temperature. This result indicates that isoprene was vaporized endothermically, and the duration time (*t*_pl_) is 1.6 min. Then, the chemical reaction contributed significantly to the heating process when the temperature began to deviate from the platform temperature. The initial exothermic onset temperature (*T*_on_) is equal to *T*_pl_. The ignition temperature (*T*_ig_) represents the temperature at which the sample begins to burn. Before ignition, a period between *T*_on_ and *T*_ig_ could be considered the ignition delay time (*t*_d_). The thermal runaway reaction reached a maximum temperature (*T*_m_) of 370.15 K, and the combustion time (*t*_c_) is defined as the period between *T*_ig_ and *T*_m_. The thermal runaway parameters are summarized in Table 3.

**Table 3:** Thermal runaway characteristic parameters of isoprene

*m*_s_ (g)	*t*_pl_ (min)	*T*_on_ (K)	*t*_d_ (min)	*T*_ig_ (K)	*t*_c_ (min)	*T*_m_ (K)	(d*T*/d*t*)_max_ (K·min^−1^)	*T*_tem_ (K)	(d*P*/d*t*)_max_ (MPa·min^−1^)	*T*_pre_ (K)
0.21	1.6	352.15	0.3	354.15	1.8	370.15	22.91	364.15	0.3021	352.15
0.42	0.3	357.9	0.3	358.15	0.9	372.15	22.92	369.40	0.5833	358.15

**Fig. 15:**
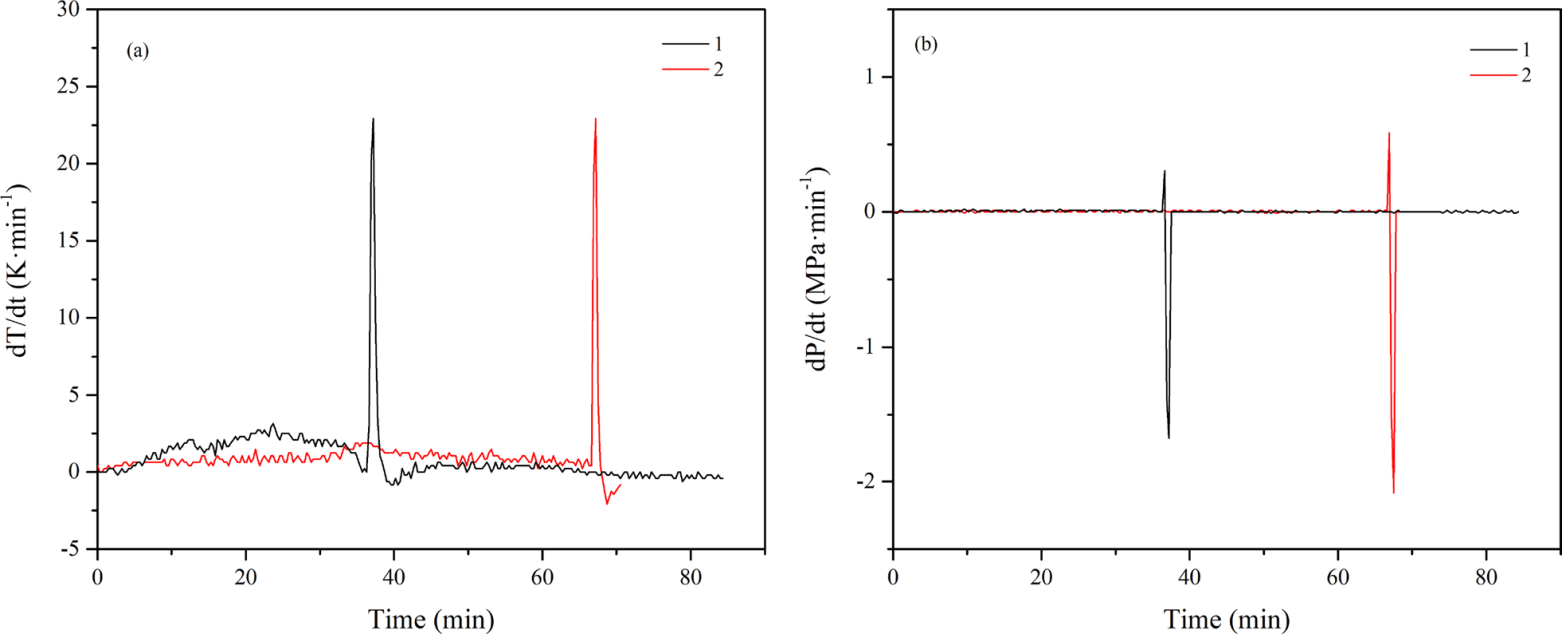
The first derivative of pressure and temperature as a function of time, 1–0.21 g isoprene; 2–0.42 g isoprene; **a** dT/dt-t; **b** dP/dt-t

Two parameters, the maximum rate of pressure rise (d*P*/d*t*)_max_ and the maximum rate of temperature rise (d*T*/d*t*)_max_, were introduced to discuss further the consequences and severity of the thermal runaway process. These are related to the rate of chemical reactions and can be used to assess explosive violence [38]. The self-temperature ramp rate (d*T*/d*t*) and self-pressure ramp rate (d*P*/d*t*) were obtained by calculating the first derivative of pressure and temperature. The parameters (d*T*/d*t*)_max_ and (d*P*/d*t*)_max_ were 22.91 K·min^−1^ and 0.3018 MPa·min^−1^, respectively. *T*_tem_ and *T*_pre_ represented the temperature at which the temperature and pressure ramp rates reach their maximum. The results are shown in Fig. 15, and the characteristic parameters of the MCPVT experiment are listed in Table 3. As a result, the accumulation and rapid pyrolysis of the isoprene peroxides released a large amount of the heat that caused the explosion.

**Fig. 16:**
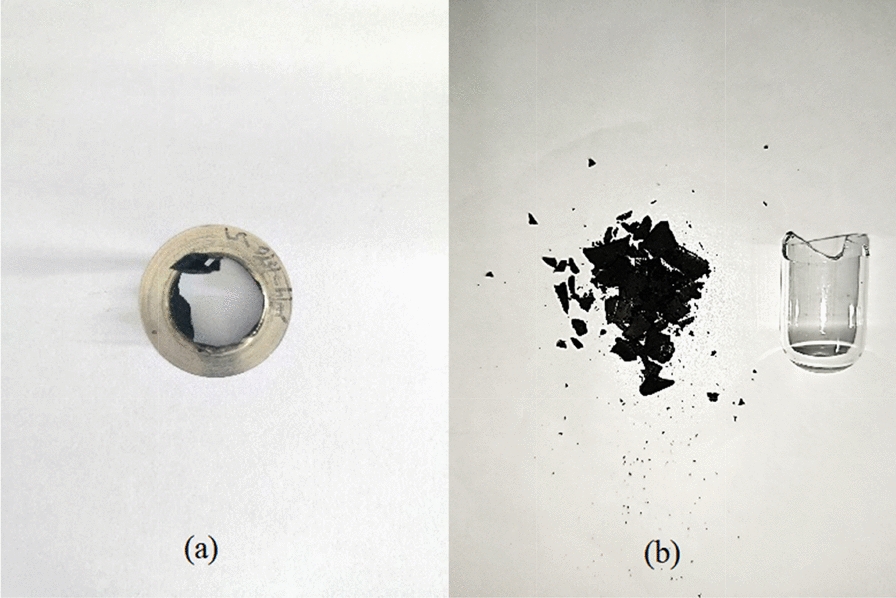
The residues after explosion of isoprene

In general, during a thermal runaway event, a sudden increase in the temperature of the reaction system is accompanied by a rapid increase in pressure, which may result in the rupture or explosion of the closed container and the loss of the container [39]. As a result, the safety relief device, which had a maximum pressure of 5 MPa, was instantly blown into pieces (Fig. 16a). The incredible power of the explosion destroyed the small beaker into fragments (Fig. 16b), indicating the magnitude of the explosion. In summary, the thermal pyrolysis of isoprene peroxide can lead to runaway phenomena and thermal explosions, significantly challenging the storage and transport of isoprene. Measures should be taken to avoid significant economic losses and injuries to people in the production and use of the isoprene process.

### Products of isoprene

The reaction products of isoprene polymerization and oxidation were determined by GC–MS. Under a nitrogen atmosphere, two isomeric dimers, including 1-methyl-4-(1-methylvinyl)cyclohexene (commonly called limonene or dipentene) and 1-methyl-5-(1-methylvinyl)cyclohexene (called sylvestrene or diprene), were generated. It indicated that isoprene could form different dimers via a [4π + 2π] cycloaddition [40].

Under an oxygen atmosphere, the oxidation products of isoprene were more complex. The results were shown in Table 4, Table 5, and presented in Additional file 1: Figs. S1–37. As shown in Table 4, the main gas products were methacrolein (MACR), methyl vinyl ketone (MVK), and 3-methylfuran, with a low isoprene conversion rate of 1.39%. This result indicated the possibility of pyrolysis of isoprene and the production of volatile products. MVK and MACR are the major initial generation products of isoprene [20, 41]. MVK is highly toxic and pose a serious risk to human health [42].

**Table 4:** Identified the gas products of isoprene

No	Products	Formula	Content/%	Similarity/%
1	Water	H_2_O	1.08	100
2	Ethanol	C_2_H_6_O	0.99	96
3	Isoprene	C_5_H_8_	96.55	97
4	Methacrolein	C_4_H_6_O	0.27	96
5	Methyl vinyl ketone	C_4_H_6_O	0.35	94
6	3-Methylfuran	C_5_H_6_O	0.77	95

**Table 5:** The liquid products of isoprene oxidation

No	Products	Molecular formula	Content/%	Similarity/%
1	Isoprene	C_5_H_8_	86.22	96
2	Methyl vinyl ketone	C_4_H_6_O	4.97	94
3	3-Methylfuran	C_5_H_6_O	1.84	95
4	3,4-Dihydro-2*H*-pyran	C_5_H_8_O	0.35	82
5	2,3-Butanedione	C_4_H_6_O_2_	0.14	91
6	2-Methyl-2-butenal	C_5_H_8_O	0.06	89
7	3-Methyl-2-buten-1-ol	C_5_H_10_O	0.13	88
8	1-Cyclopropyl-1-pentanol	C_8_H_16_O	0.46	83
9	Allyl carbonate	C_7_H_10_O_3_	0.09	86
10	2-Methyl-1-butene	C_5_H_10_	0.30	85
11	Tetrahydrofurfuryl alcohol	C_5_H_10_O_2_	0.80	90
12	2,5-Dihydrofuran	C_4_H_6_O	0.17	85
13	2,3-Dihydrofuran	C_4_H_6_O	0.70	86
14	2-Butenoic acid	C_5_H_8_O_2_	1.14	81
15	(1R,3S)-4-cyclopentene-1,3-diol	C_5_H_8_O_2_	0.15	84
16	4,5-Dimethyl-2-hepten-3-ol	C_9_H_18_O	0.15	92
17	3-Methyl-2(5*H*)-furanone	C_5_H_6_O_2_	0.15	92
18	Tricyclo[5.2.1.0^2,6^]dec-8-ene	C_10_H_14_	0.02	94
19	Sylvestrene	C_10_H_16_	0.22	93
20	Limonene	C_10_H_16_	0.07	92
21	3-Methyl-1-pentanol	C_6_H_14_O	0.19	87
22	(2*Z*)-2-Methyl-2-penten-1-ol	C_6_H_12_O	0.72	83
23	1-Bicyclo[2.2.1]hept-5-en-2-ylethanone	C_9_H_12_O	0.02	95
24	1-(1,2-dimethyl-2-cyclopenten-1-yl)ethanone	C_9_H_14_O	0.29	92
25	4-Acetyl-1-methylcyclohexene	C_9_H_14_O	0.65	97

The liquid products are shown in Table 5. The products are complex and can give rise to pyrolysis, oxidation, and polymerization products. These components are classified into five fractions: alkenes, aldehydes, ketones, alcohols, and other oxygen-containing compounds (O-compounds). Compared to 1,3-butadiene, a methyl in the isoprene structure makes its reactivity more varied, reflected in its conjugated double bond being easier to react with electrophiles and Diels–Alder dienophiles [43]. Isoprene possesses four types of hydrogen atoms that can be involved in many reactions, including substitution, addition, ring formation, complexation, and telomerization [44].

The main products were methyl vinyl ketone (MVK), 3-methylfuran, 2-butenoic acid, 1-(1,2-dimethyl-2-cyclopenten-1-yl)ethenone, 4-acetyl-1-methylcyclohexene, etc. These were different from previous reports. Kuznetsova et al.[45] reported that the carbonyl compounds formed during autoxidation of isoprene were HCHO, methacrolein (MACR), MVK, meiso-prketone, and polyfunctional CO compounds. Karakozova et al.[46] studied that the oxidation of isoprene by oxygen in the presence of a system consisting of TPPMn^III^Cl and sodium borohydride, giving only 3-methyl-2-butenol. Wennberg et al.[17] researched that the products of isoprene with ·OH were MVK, MACR, ISOPOOH, and IEPOX. Ma et al.[47] explored the reaction between isoprene and chlorine radical (·Cl) and can produced MVK, MACR, and highly oxidized organic mols. (HOMs).

The composition of the explosion products was also determined, as shown in Tables 6 and 7 (Additional file 1: Figs. S37–58). The data in Table 6 displayed that the primary gas products were propene and 2-butene, yielding 15.30% and 20.47%, respectively. This result indicated that isoprene undergoes a relatively violent pyrolysis reaction, resulting in the formation of small molecule products.

**Table 6:** The gas products of the thermal runaway of isoprene

No	Products	Formula	Content/%	Similarity/%
1	Propene	C_3_H_6_	15.30	88
2	2-Butene	C_4_H_8_	20.47	94
3	3-Methyl-1-butene	C_5_H_10_	5.29	94
4	Isopentane	C_5_H_12_	0.34	96
5	Isoprene	C_5_H_8_	50.67	97
6	2-Methyl-1-butene	C_5_H_10_	4.40	96
7	Cyclopentene	C_5_H_8_	0.44	97
8	3-Methyl-2-pentene	C_6_H_12_	0.49	89
9	Methacrolein	C_4_H_6_O	0.65	96
10	2-Methyl-1-pentene	C_6_H_12_	0.23	88
11	Isobutyraldehyde	C_4_H_8_O	0.11	86
12	2,3-Dimethyl-2-butene	C_6_H_12_	0.26	93
13	3-Methylfuran	C_5_H_6_O	0.91	95
14	Benzene	C_6_H_6_	0.47	81

**Table 7:** The liquid products of the thermal runaway of isoprene

No	Products	Formula	Content/%	Similarity/%
1	Water	H_2_O	18.58	100
2	Ethanol	C_2_H_6_O	3.67	93
3	Dimethoxymethane	C_3_H_8_O_2_	11.85	95
4	2,3-Pentanedione	C_5_H_8_O_2_	57.45	84
5	Butyl acetate	C_6_H_12_O_2_	2.68	93
6	Naphthalene	C_10_H_8_	2.62	93
7	Acenaphthylene	C_12_H_8_	1.01	92
8	9-Methylene-9*H*-fluorene	C_14_H_10_	2.15	91

The data in Table 7 revealed that dimethoxymethane and 2,3-pentanedione are the major species of liquid products. It inferred from the explosion products that all the fragments were challenging to produce by direct fragmentation of the isoprene molecules. It was previously assumed that a large number of isoprene peroxides were accelerated in the system and further decomposed to release a significant amount of heat, leading to explosive reactions. The explosive reaction was so violent that complex products were formed, such as naphthalene, acenaphthylene, and 9-methylene-9H-fluorene. The products before and after the explosion were vastly different. The reason is that isoprene peroxides were broken down to different degrees. The rapid decomposition of isoprene peroxide released a large amount of heat, and the rapidly rising temperature could induce pyrolysis of the substances in the system. At moderate temperatures, the less and slower decomposition of isoprene peroxide does not lead to temperature changes. Decomposable peroxides occurred in the deactivation reactions to form linear and cyclic products.

### Possible pathways of isoprene

**Scheme 1:**
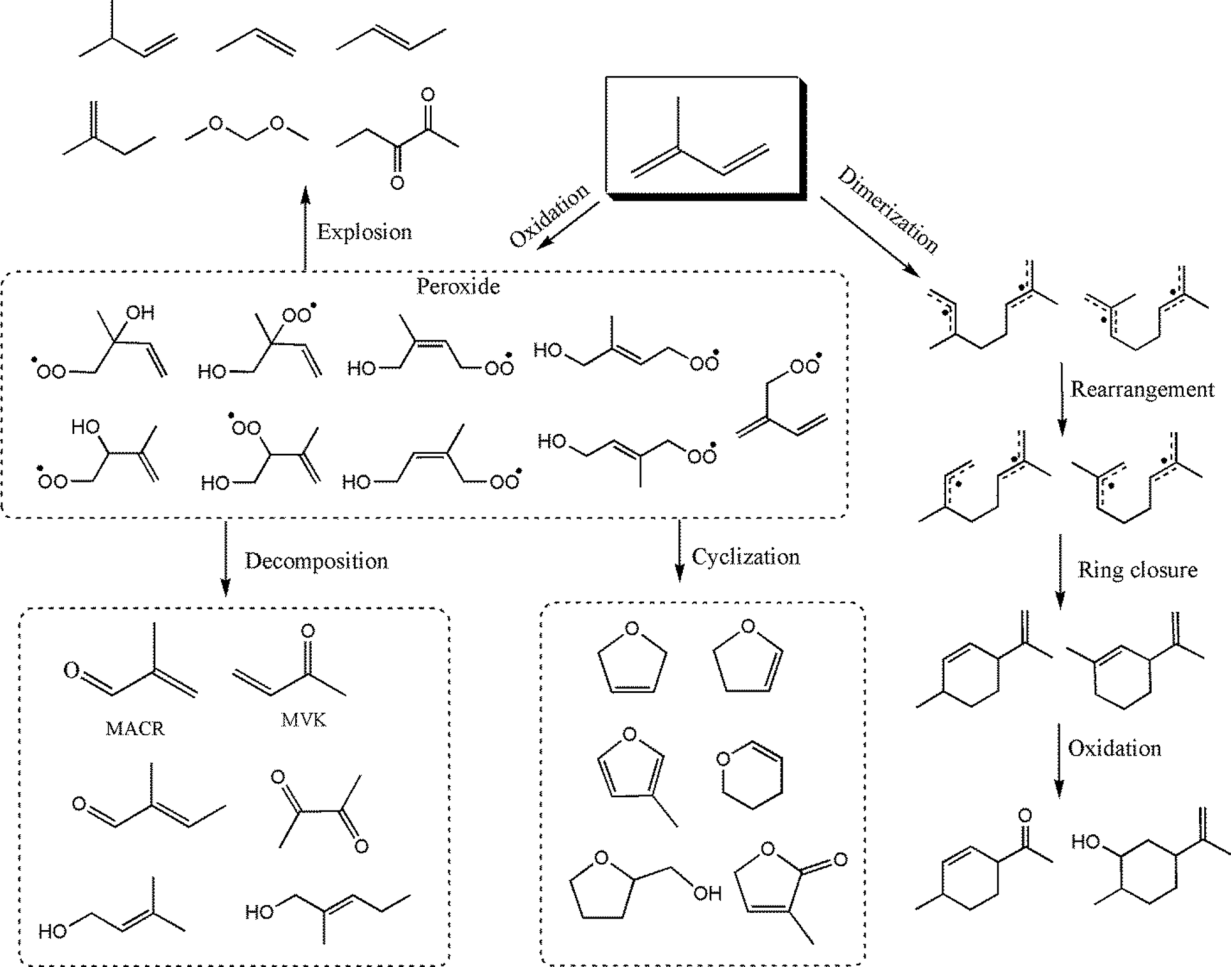
Probable reaction pathways of isoprene with oxygen

A possible pathway for isoprene oxidation and polymerization is suggested based on previous work predicting a reaction mechanism as shown in Scheme 1. The hydrogen atom from the methyl group of isoprene was abstracted to form allyl radicals (·C_5_H_7_). Guo et al.[10] proposed a successive cyclization-driven autoxidation mechanism of allylic radicals from isoprene calculated by density functional theory (DFT). Oxygen was added to the C5 atom of ·C_5_H_7_ to form a hydroxy peroxy intermediate. Additionally, the subsequent reaction of isoprene with ·OH and oxygen proceeds mainly via the addition of the conjugated double bonds to form the isomer of isoprene hydroxy peroxy radicals (ISOPOO) [17]. These radicals undergo H-shifts (via 1,3-, 1,5-, and 1,6-H shifts) from different − CH_x_ (x = 1,2) sites to the terminal O-atom to form hydroxy peroxy radicals. Pyrolysis of hydroxy peroxides was believed to occur, resulting in the formation of MVK, MACR, CH_2_O, ·OH, and unsaturated alcohols [14].

In addition, isoprene hydroxy peroxides are often used to form five- and six-membered rings by cyclization reaction. Gu et al. [48, 49] reported two possible reaction pathways for the formation of 3-methylfuran by initiating ·OH radical addition at the terminal = CH_2_ group. The first is that the alkoxy radicals continued to be oxidized and then occurred through ring closure and loss of water to generate 3-methylfuran. The other is that the alkoxy radical undergoes an intramolecular rearrangement and cyclization to form an epoxy radical. It would be oxidized and lose water to form 3-methylfuran [50]. In addition, the selective oxidation of isoprene to methylmaleic anhydride (citraconic anhydride) was discussed by Philipps et al.[51] Cabello et al. studied the oxidation of isoprene over vanadium oxide-based catalysts, and the ring products included 3-methylfuran, 2,5-dihydrofuran, and 3-methyl-2(5H)-furanone [52].

On the other hand, dimerization of isoprene was often accompanied by oxidation processes. The dimerization of isoprene represents a typical Diels–Alder condensation [30]. This reaction would occur through the formation of an open-chain diradical intermediate, which then undergoes a ring closure reaction. An open-chain diradical intermediate, consisting of two charges, both delocalized on tertiary carbon atoms, undergoes ring closure to form sylvestrene. From the steric point of view, the possible formation mechanism of limonene may be following a concerted mechanism [40]. It was found that the intermediate formation of limonene differs from that of sylvestrene. Thus, elucidating the structure of the diradical intermediate is key to a fundamental understanding of the dimerization product of isoprene.

## Conclusion

The thermal reaction of isoprene in nitrogen and oxygen atmospheres was measured with a custom-designed MCPVT device and the thermal runaway properties of isoprene were analyzed. The main conclusions drawn can be summarized as follows.

1. Under a nitrogen atmosphere, isoprene was first vaporized significantly and then polymerized. Isoprene gave rise to two different isomeric dimers via a [4π + 2π] cycloaddition.
2. The oxidation process of isoprene occurred in three steps under an oxygen atmosphere: initially, isoprene reacted with oxygen to form peroxides; second, peroxides further decompose to form free radicals; finally, free radicals are involved in complex pyrolysis and explosive reactions. Two kinetic models of isoprene were studied, and the activation energies are 86.88 kJ·mol^−1^ and 96.79 kJ·mol^−1^, respectively.
3. The formation of isoprene peroxide was identified, and its thermal decomposition characteristic was determined by DSC. The onset temperature *T*_on_ and decomposition heat *Q*_DSC_ were obtained for the total oxidation product and the purified peroxide mixture.
4. Thermal runaway experiments were discussed in different conditions. It was shown that thermal runaway reactions are prone to occur as the amount of reactants increases.
5. The chemical compositions of the reaction products and the residues after the thermal runaway were also analyzed. The oxidation and polymerization mechanisms of isoprene have been systematically analyzed based on thermal runaway and chemical analysis experiments.

This work will contribute to a better understanding of the thermal reaction properties and explosion hazards of isoprene and provide a valuable reference for determining safety limits in practice.

### Supplementary Information

**Additional file 1. **Additional figures.

## Data Availability

All data generated or analyzed during this study are included in this published article.
