# Supplementary material for: Oxidation characteristic and thermal runaway of isoprene
Source: BMC Chem. 2023 Sep 2;17(1):110. doi: 10.1186/s13065-023-01016-y (PMC10475201; doi:10.1186/s13065-023-01016-y)
Supplement: Supplementary file 1 — Additional file 1. Additional figures. [file 13065_2023_1016_MOESM1_ESM.docx]

##### Thermal stability and explosion hazard of isoprene

Min Liang, Suyi Dai, Haijun Cheng, Chang Yu, Weiguang Li, Fang Lai, Kang Yang, Li Ma*, Xiongmin Liu*

*School of Chemistry and Chemical Engineering, Guangxi University, Nanning 530004, China*

**SUPPORTING INFORMATION**

**Figure S1.** The Total ion chromatography of the gas products of isoprene pyrolysis.

**Figure S2-S5.** The mass spectra of the gas products of isoprene pyrolysis.

**Figure S6.** The Total ion chromatography of the liquid products of isoprene pyrolysis.

**Figure S7-S36.** The mass spectra of the liquid products of isoprene pyrolysis.

**Figure S37.** The Total ion chromatography of the gas products of isoprene after the explosion.

**Figure S38-S51.** The mass spectra of the gas products of isoprene after the explosion.

**Figure S52.** The Total ion chromatography of the liquid products of isoprene after the explosion.

**Figure S53-S58.** The mass spectra of the liquid products of isoprene after the explosion.

* Corresponding author. *E-mail addresses:* xmliu1@gxu.edu.cn (X.-M. Liu), gxumali@126.com (L. Ma)

**
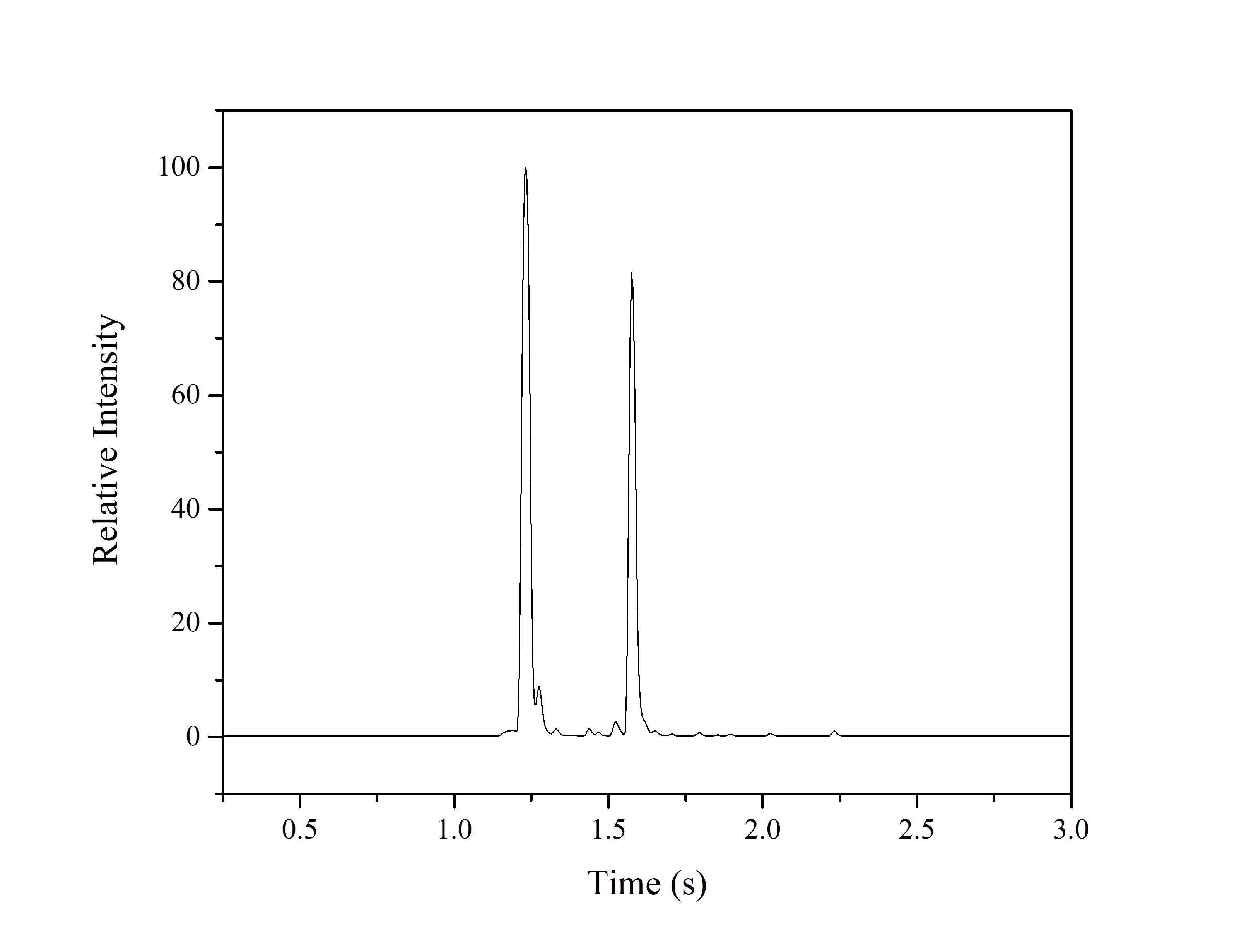
**

**Figure S1.** The Total ion chromatography of the gas products of isoprene pyrolysis.


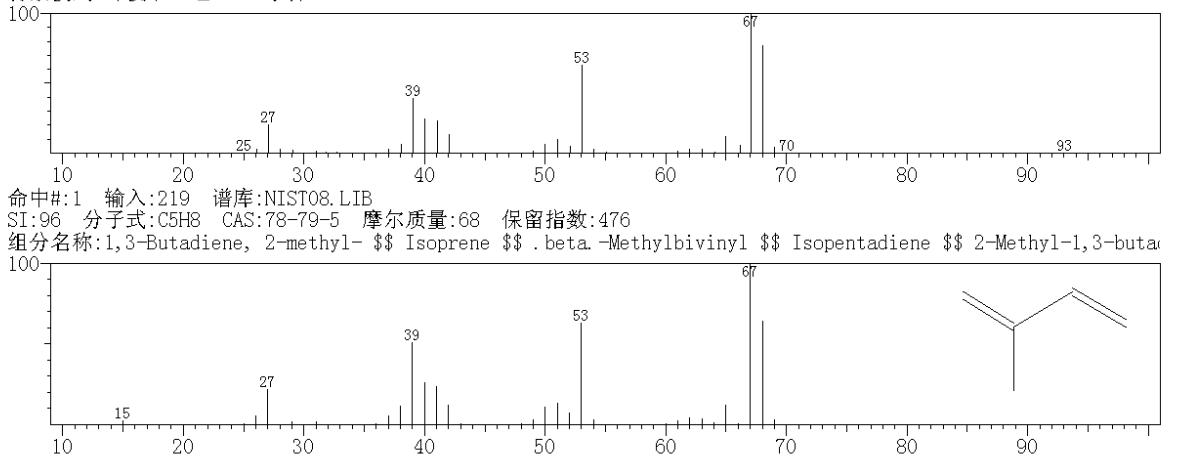


(a) MS spectra of the product


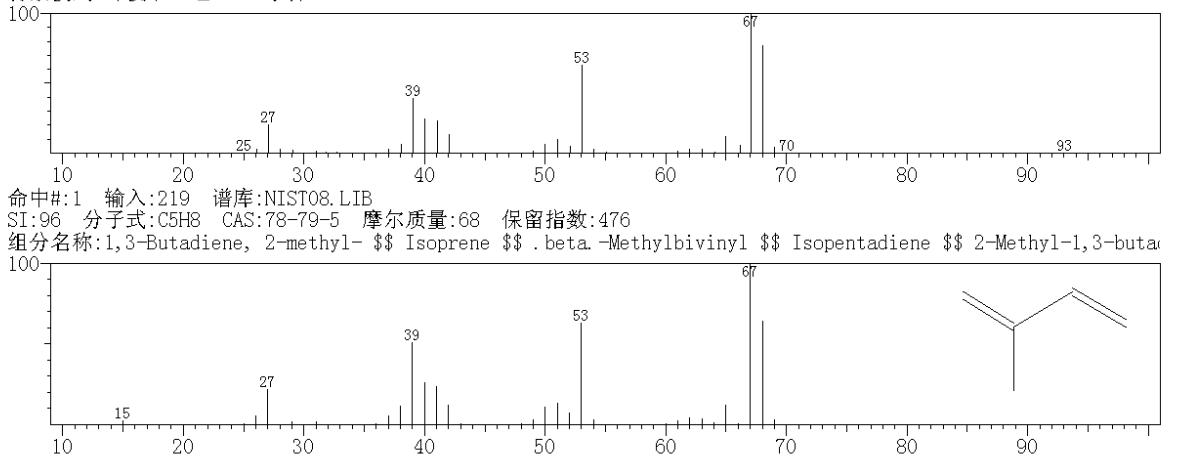


(b) MS spectra of the isoprene

**Figure S2.** The mass spectra of the gas products of isoprene pyrolysis. (Isoprene)


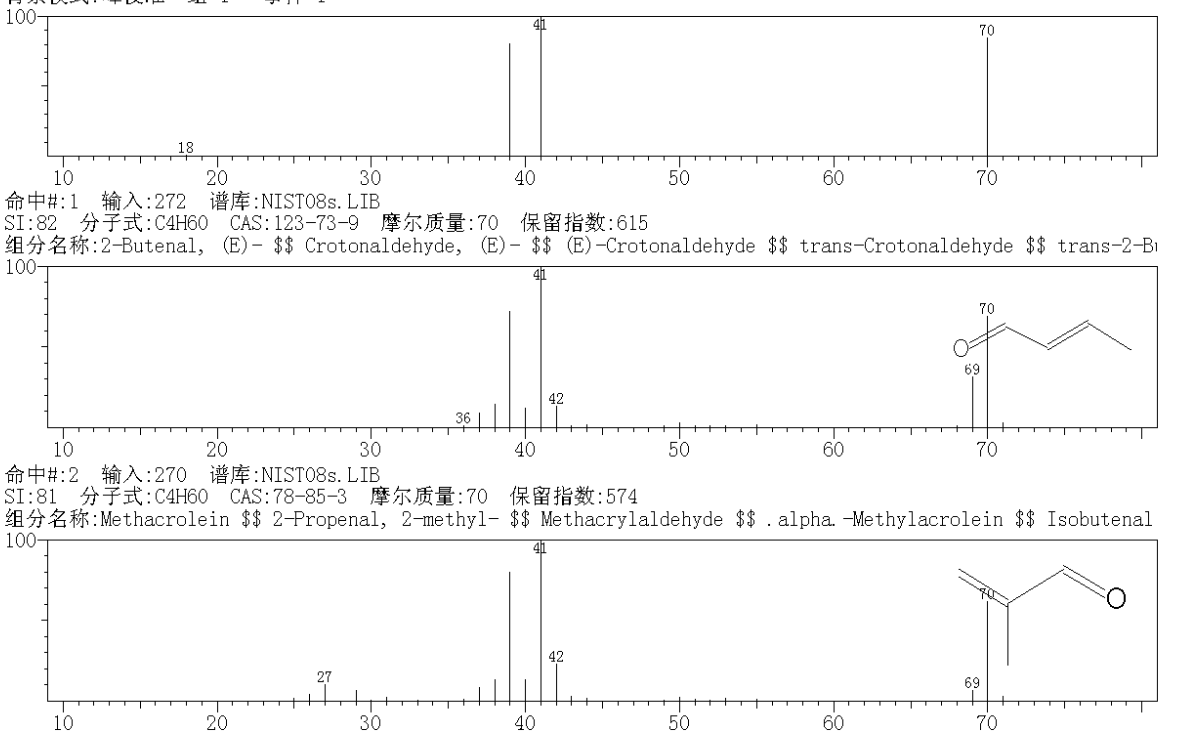


(a) MS spectra of the product


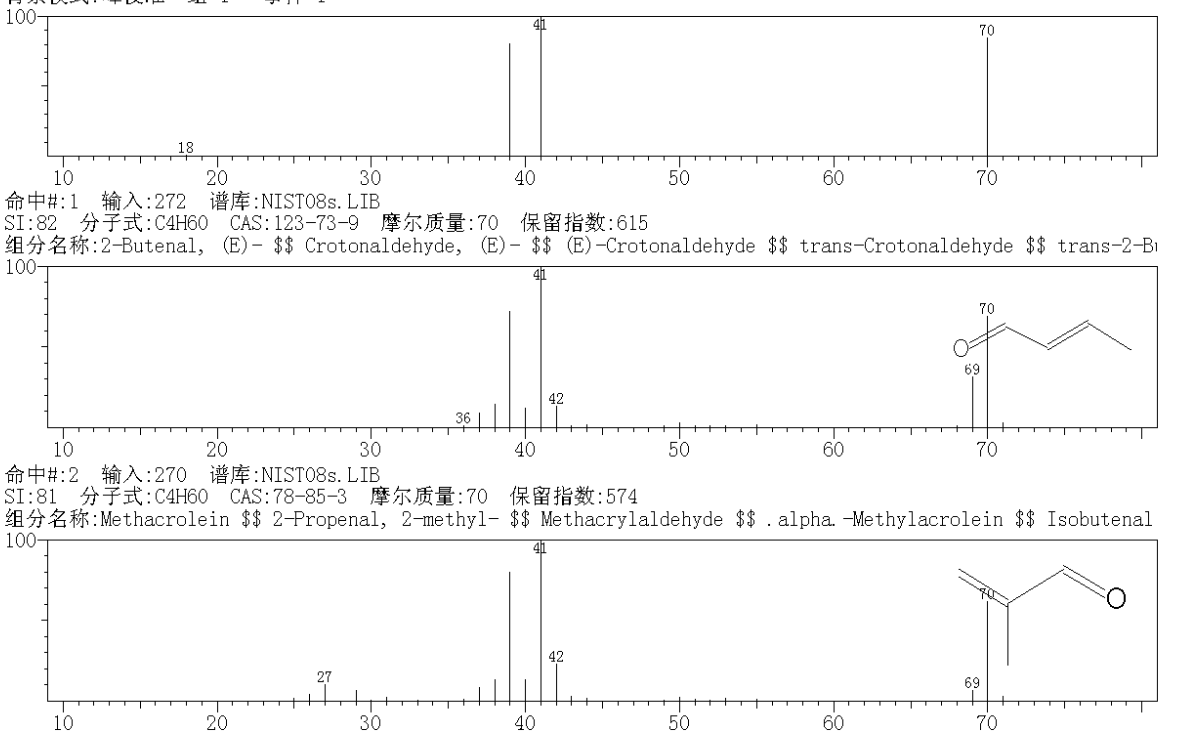


(b) MS spectra of the methacrolein

**Figure S3.** The mass spectra of the gas products of isoprene pyrolysis. (Methacrolein)


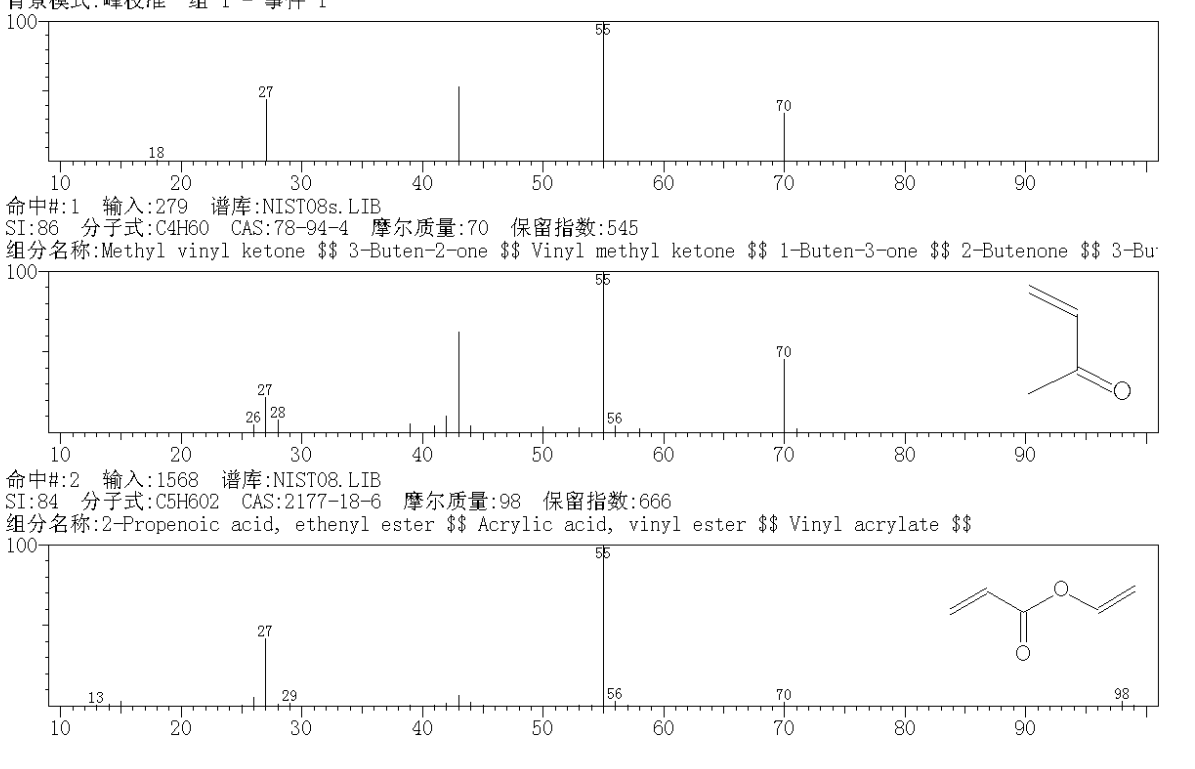


(a) MS spectra of the product


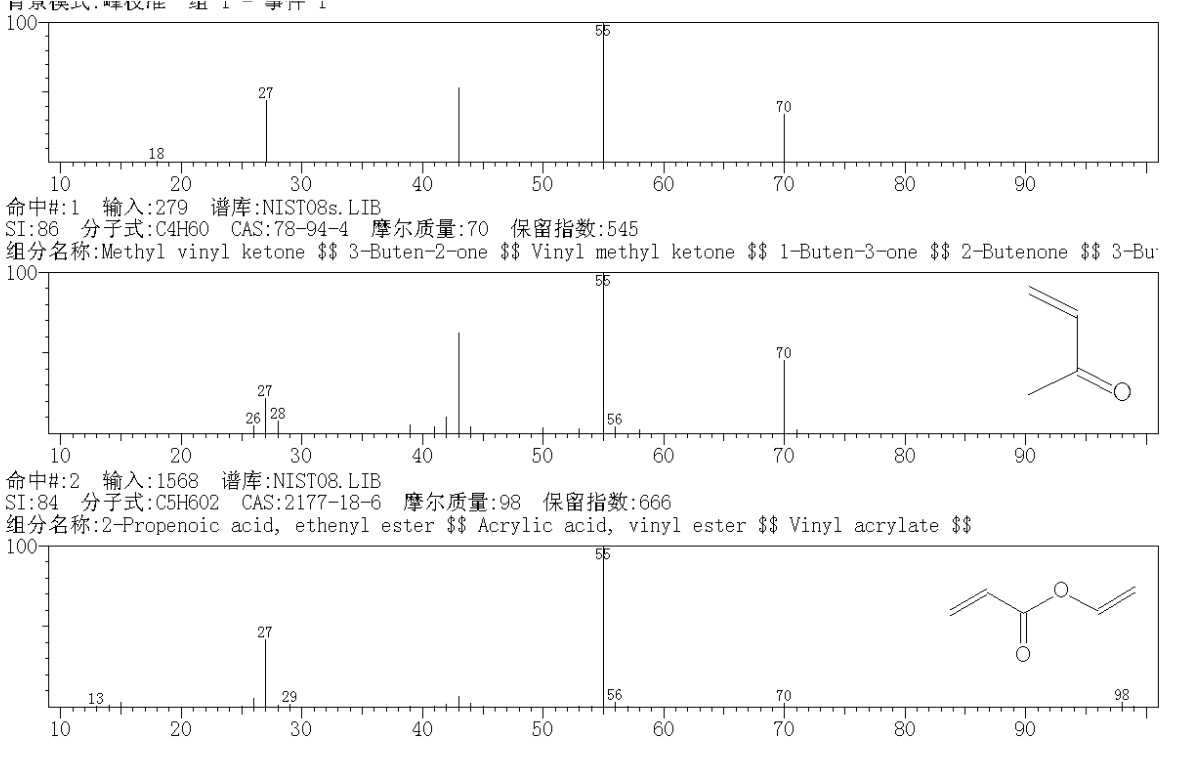


(b) MS spectra of the methyl vinyl ketone

**Figure S4.** The mass spectra of the gas products of isoprene pyrolysis. (Methyl vinyl ketone)


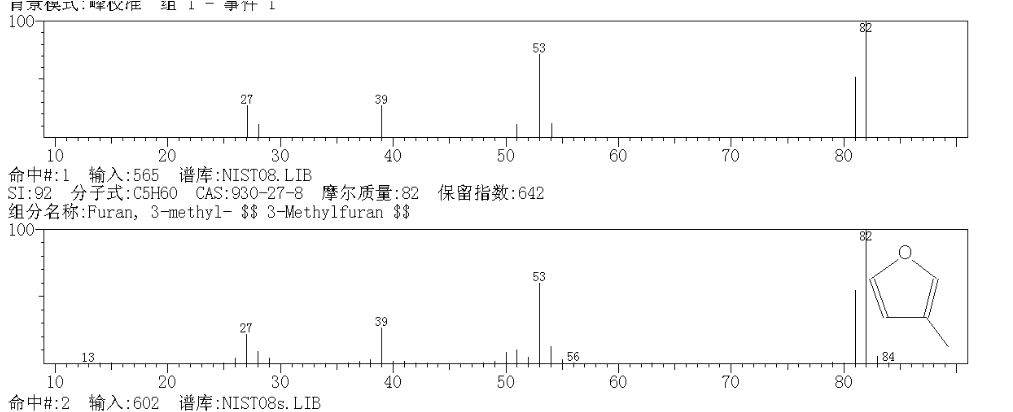


(a) MS spectra of the product


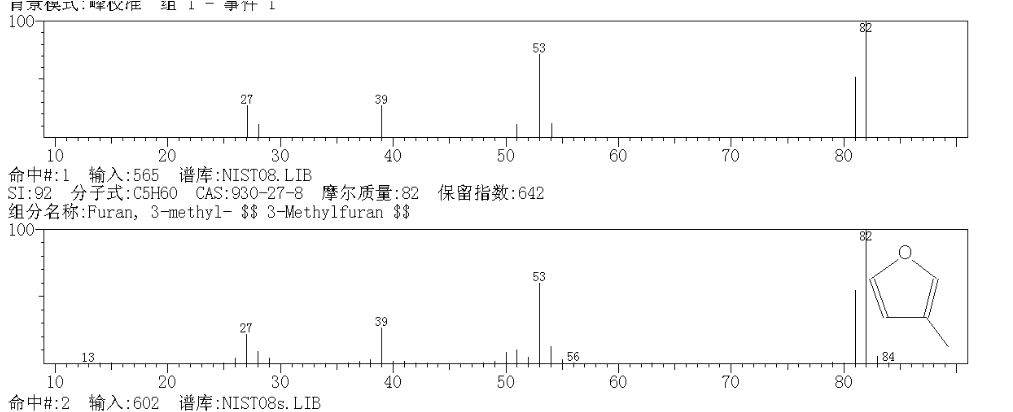


(b) MS spectra of the 3-methylfuran

**Figure S5.** The mass spectra of the gas products of isoprene pyrolysis. (3-methylfuran)

**
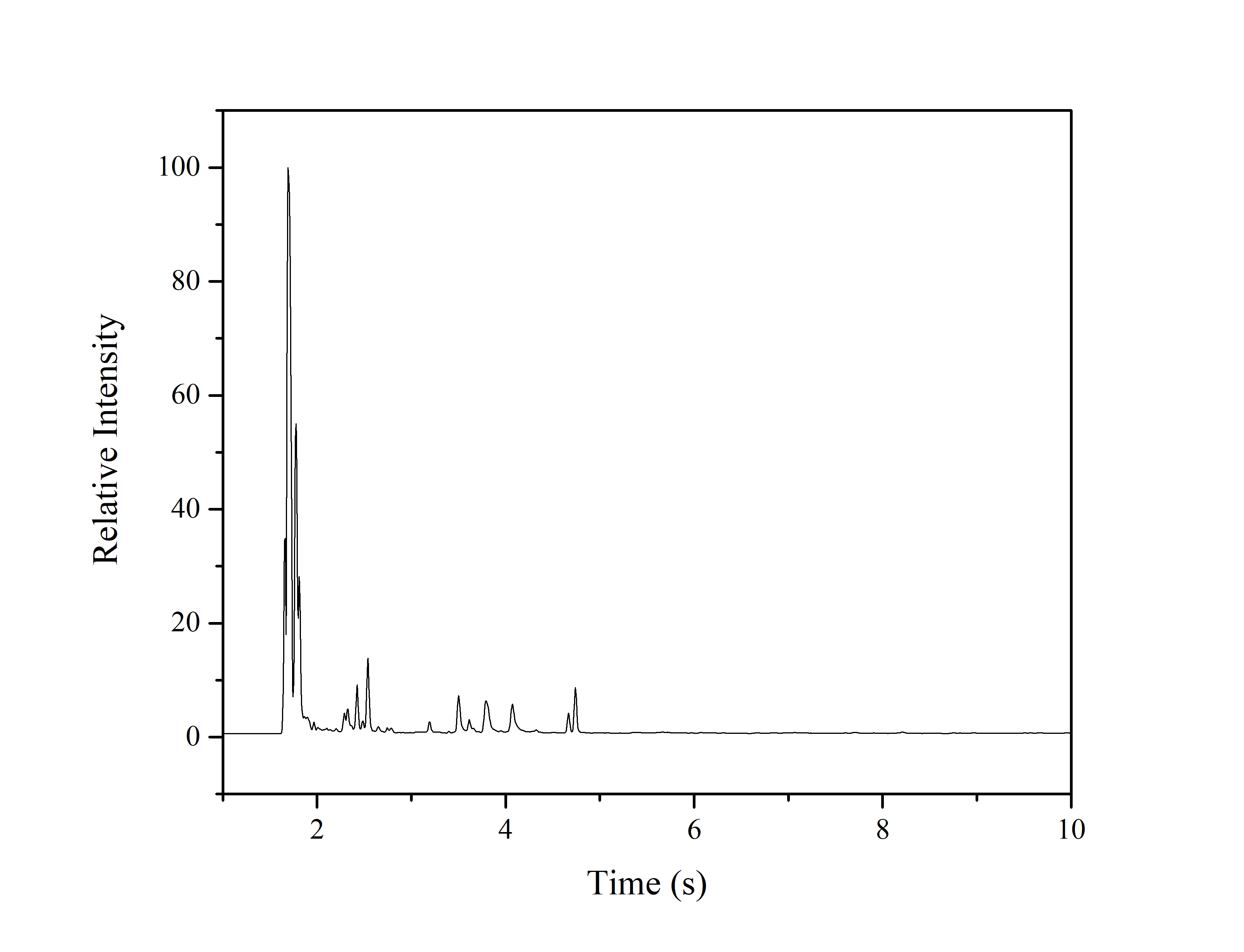
**

**Figure S6.** The Total ion chromatography of the liquid products of isoprene pyrolysis.


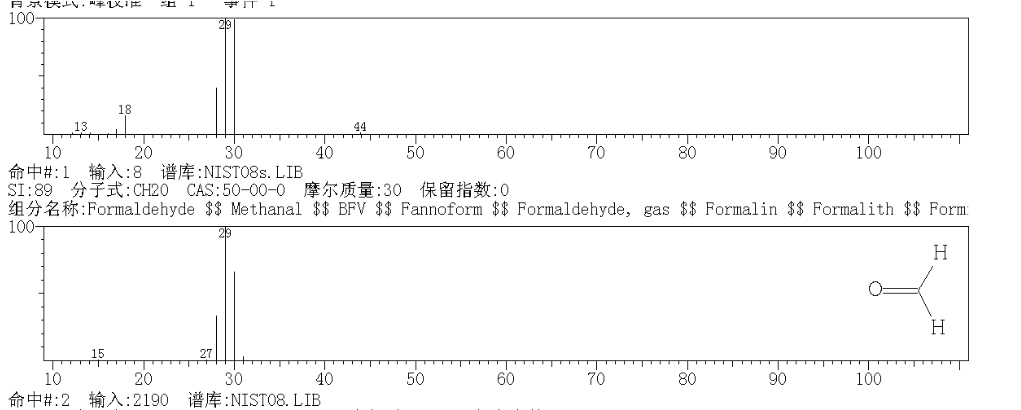


(a) MS spectra of the product


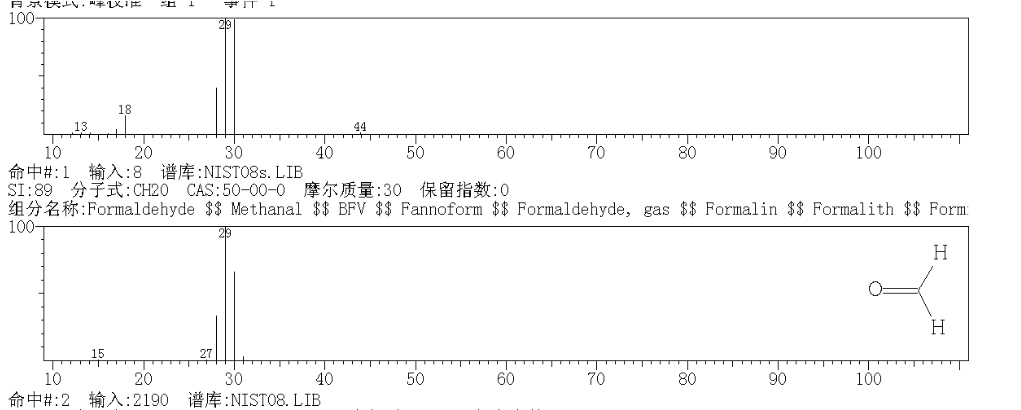


(b) MS spectra of the formaldehyde

**Figure S7.** The mass spectra of the liquid products of isoprene pyrolysis. (Formaldehyde)


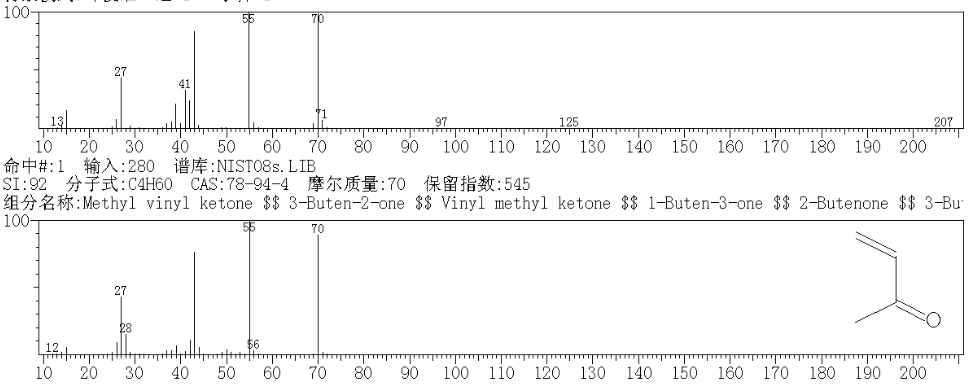


(a) MS spectra of the product


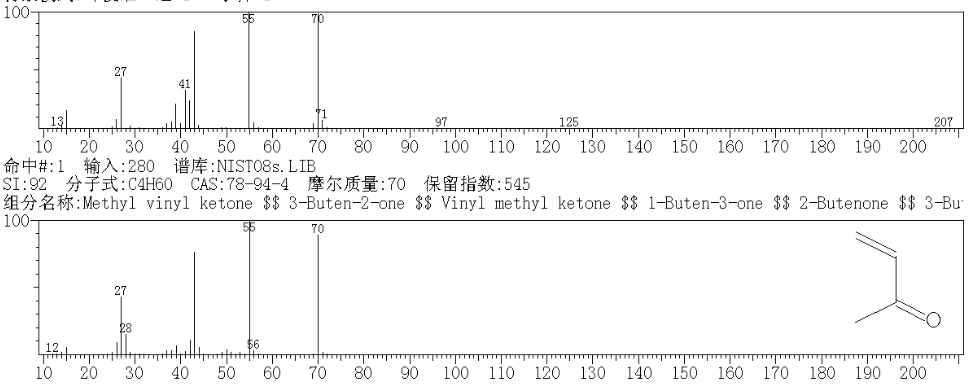


(b) MS spectra of the methyl vinyl ketone

**Figure S8.** The mass spectra of the liquid products of isoprene pyrolysis. (Methyl vinyl ketone)


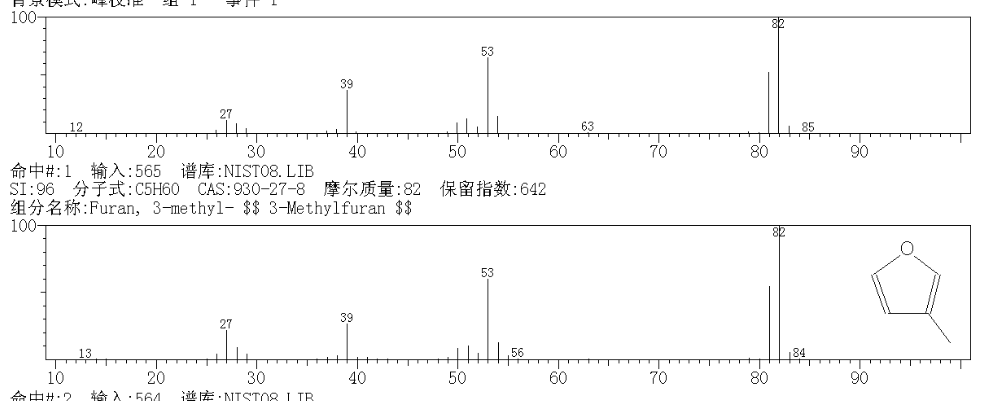


(a) MS spectra of the product


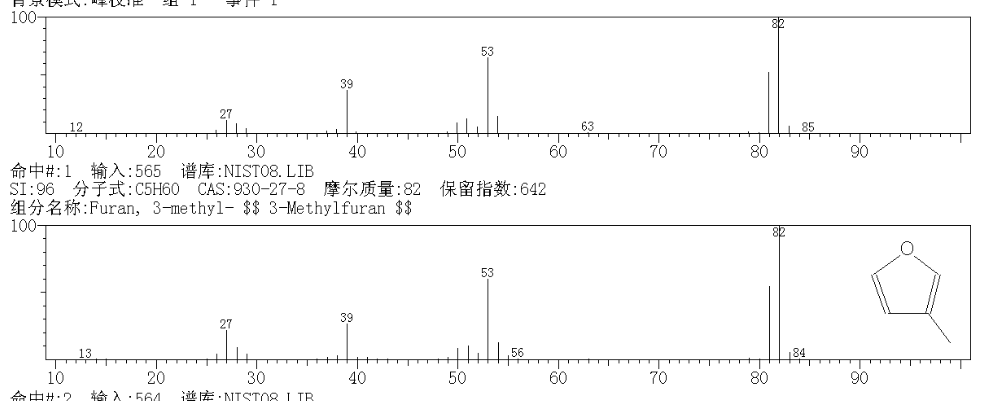


(b) MS spectra of the 3-methylfuran

**Figure S9.** The mass spectra of the liquid products of isoprene pyrolysis. (3-Methylfuran)


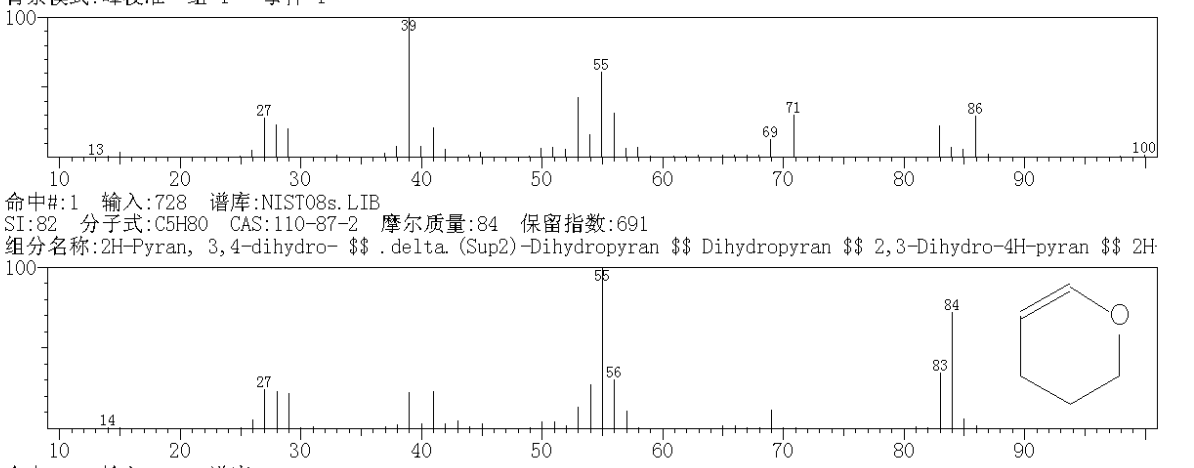


(a) MS spectra of the product


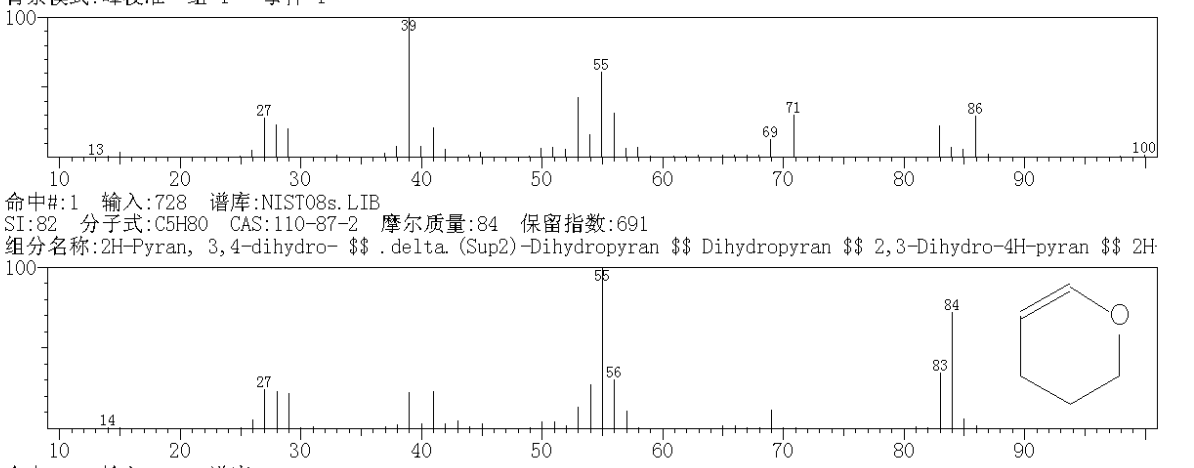


(b) MS spectra of the 3,4-dihydro-2*H*-pyran

**Figure S10.** The mass spectra of the liquid products of isoprene pyrolysis. (3,4-Dihydro-2*H*-pyran)


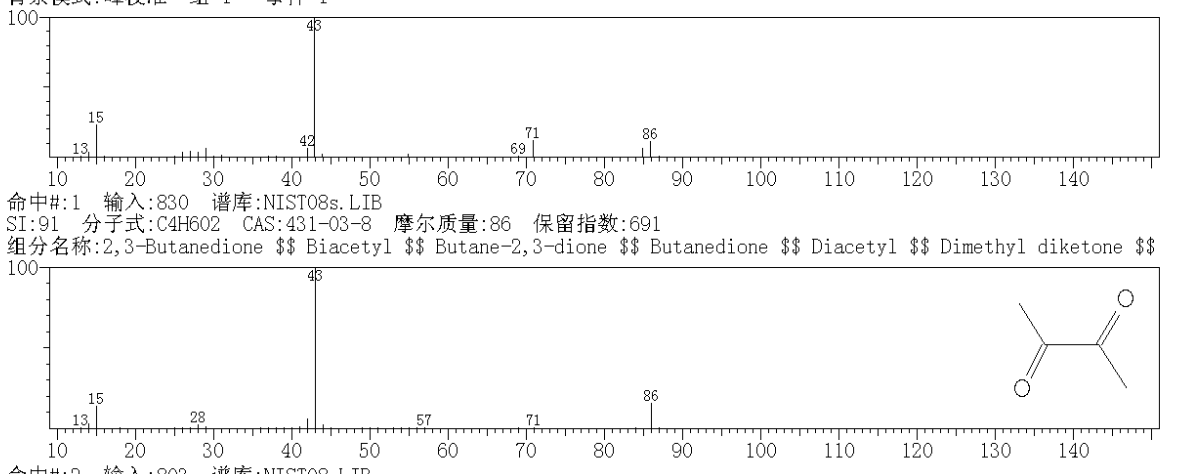


(a) MS spectra of the product


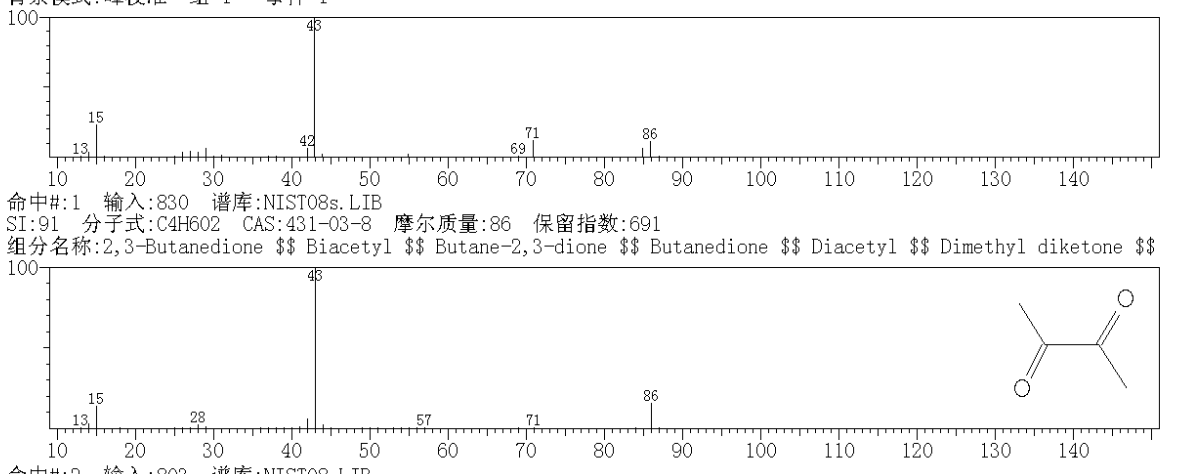


(b) MS spectra of the 2,3-butanedione

**Figure S11.** The mass spectra of the liquid products of isoprene pyrolysis. (2,3-Butanedione)


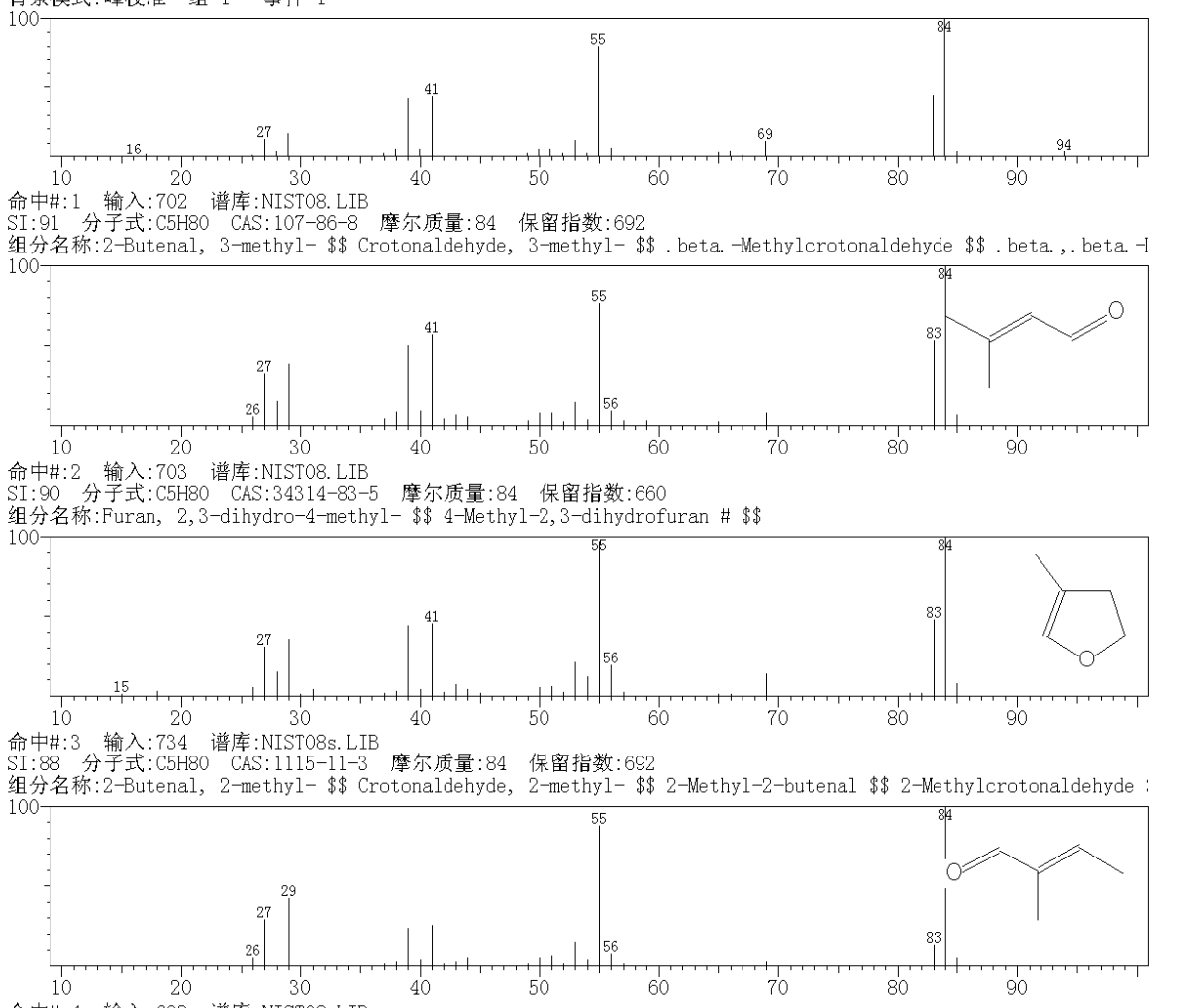


(a) MS spectra of the product


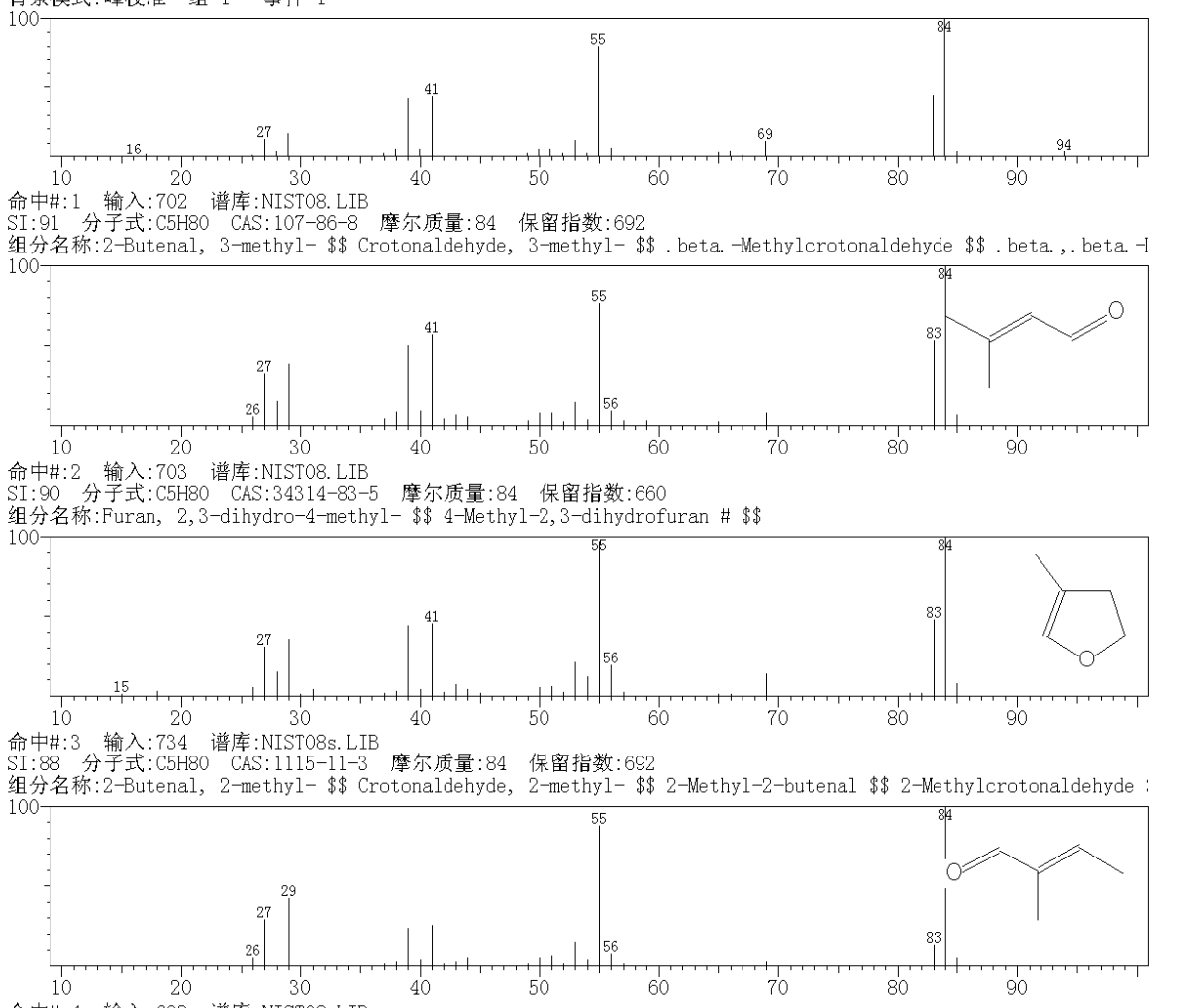


(b) MS spectra of the 2-methyl-2-butenal

**Figure S12.** The mass spectra of the liquid products of isoprene pyrolysis. (2-Methyl-2-butenal)


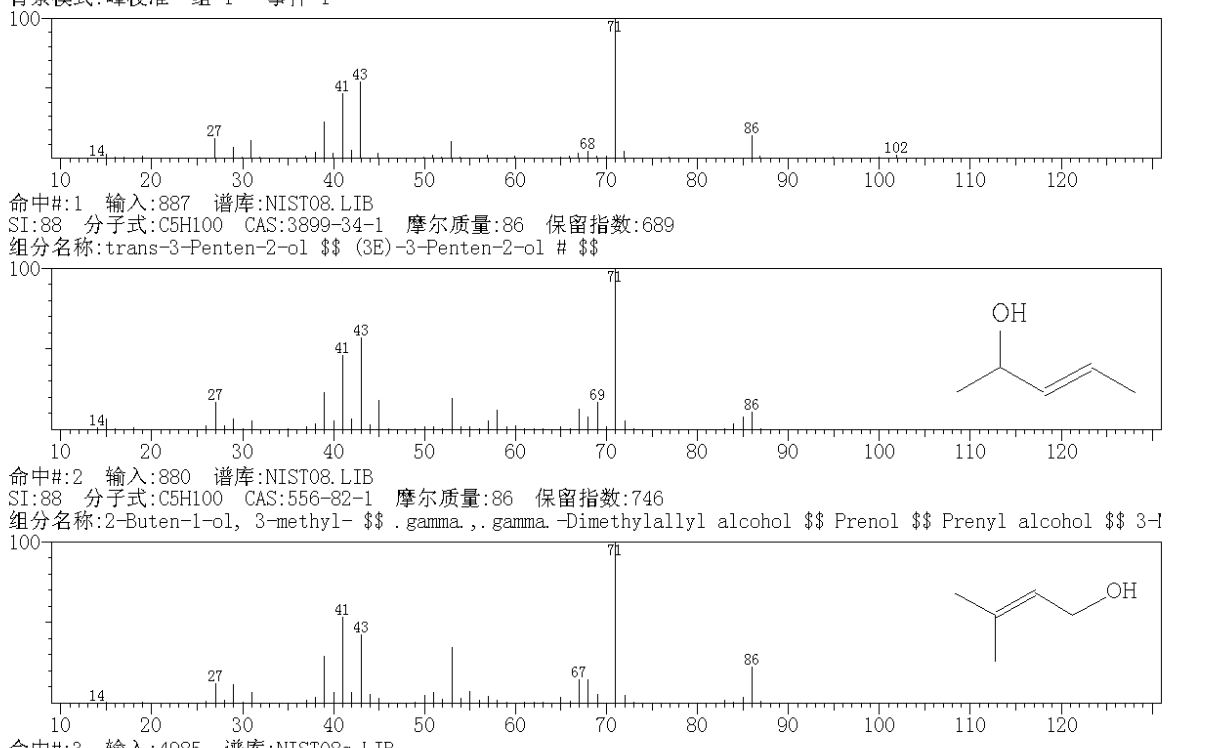


(a) MS spectra of the product


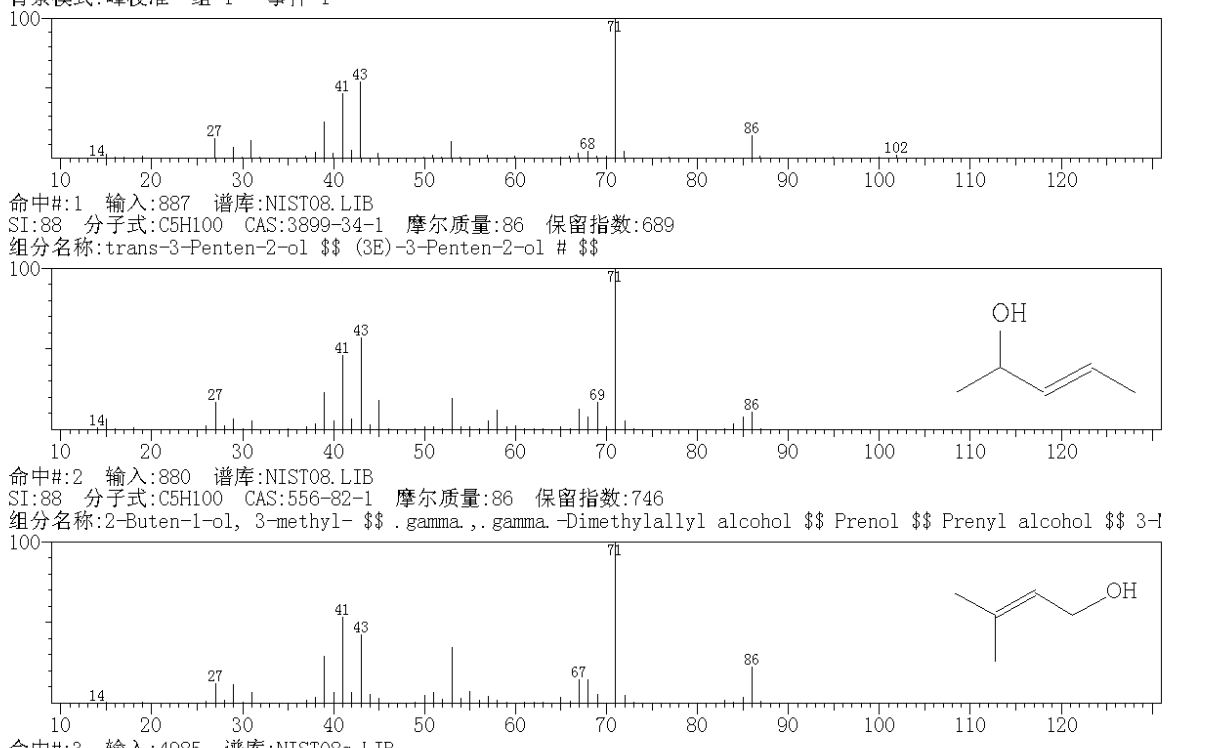


(b) MS spectra of the 3-methyl-2-buten-1-ol

**Figure S13.** The mass spectra of the liquid products of isoprene pyrolysis. (3-Methyl-2-buten-1-ol)


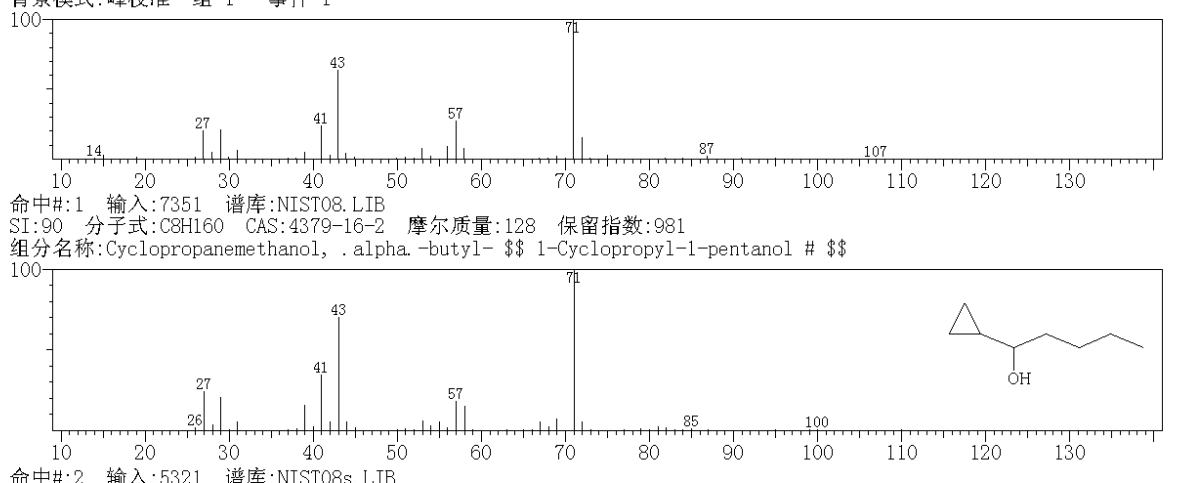


(a) MS spectra of the product


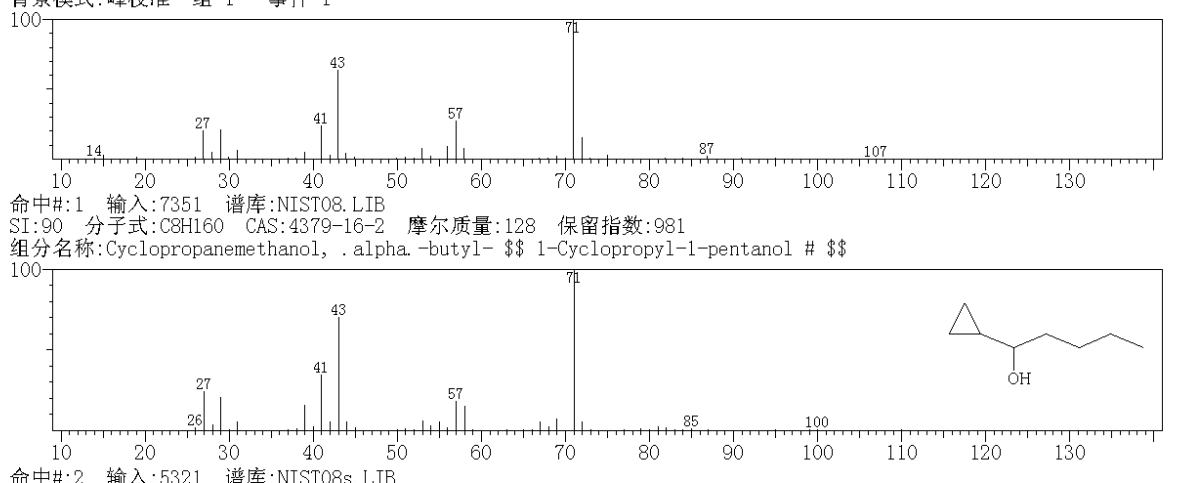


(b) MS spectra of the 1-cyclopropyl-1-pentanol

**Figure S14.** The mass spectra of the liquid products of isoprene pyrolysis. (1-Cyclopropyl-1-pentanol)


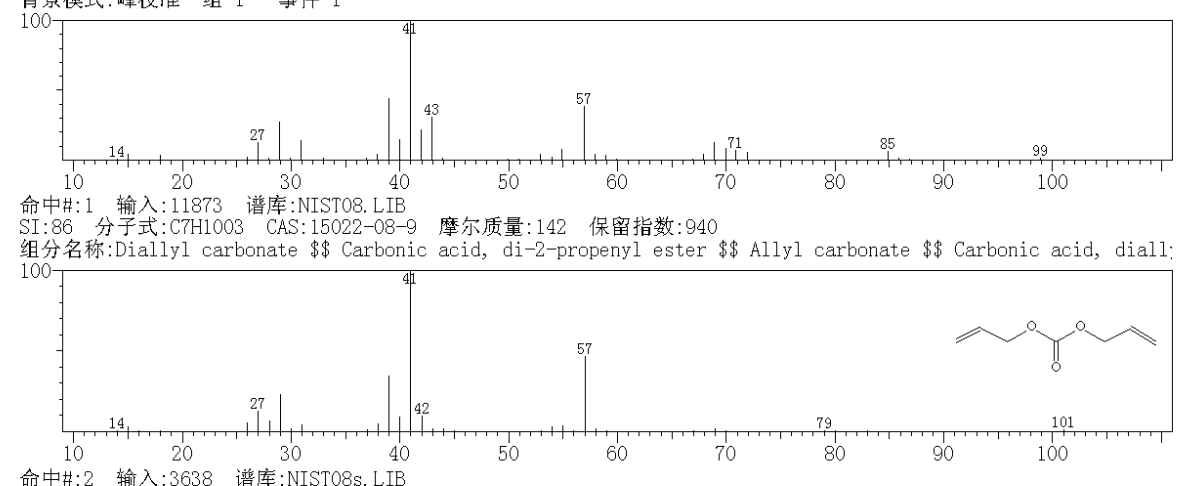


(a) MS spectra of the product


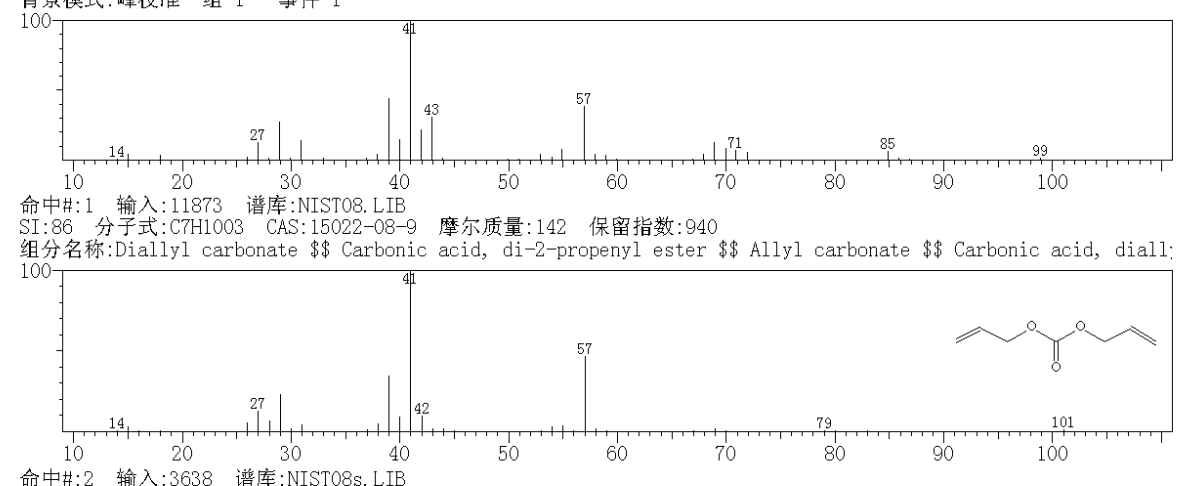


(b) MS spectra of the allyl carbonate

**Figure S15.** The mass spectra of the liquid products of isoprene pyrolysis. (Allyl carbonate)


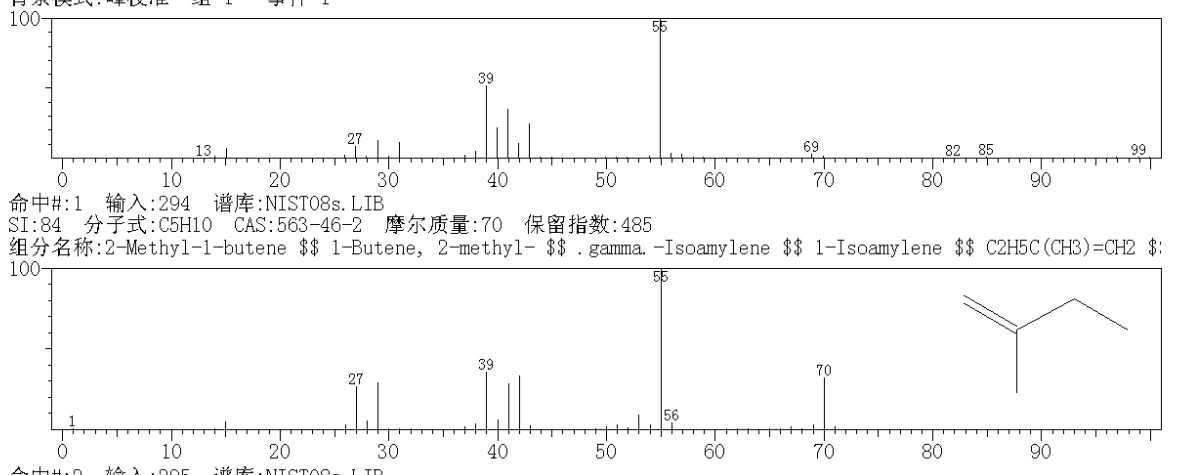


(a) MS spectra of the product


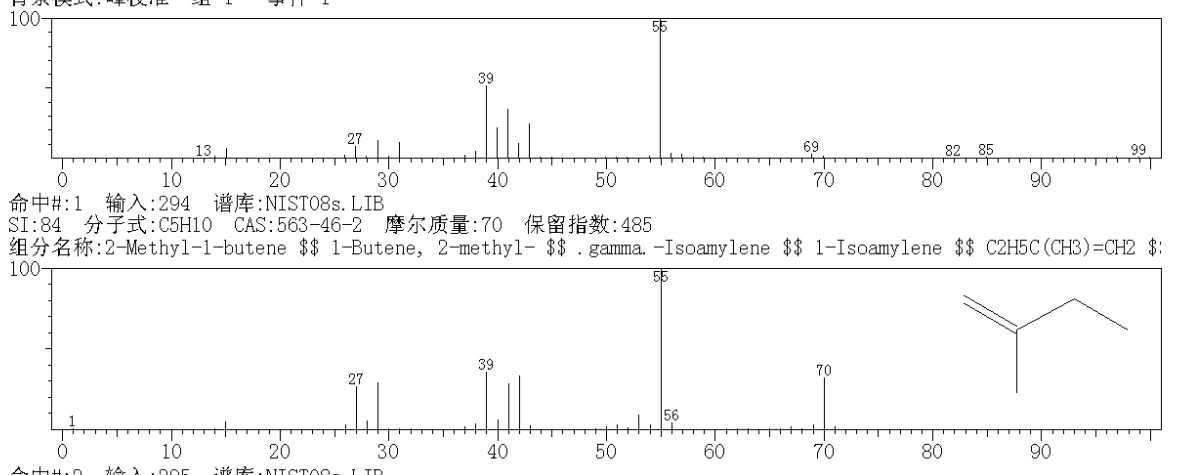


(b) MS spectra of the 2-methyl-1-butene

**Figure S16.** The mass spectra of the liquid products of isoprene pyrolysis. (2-Methyl-1-butene)


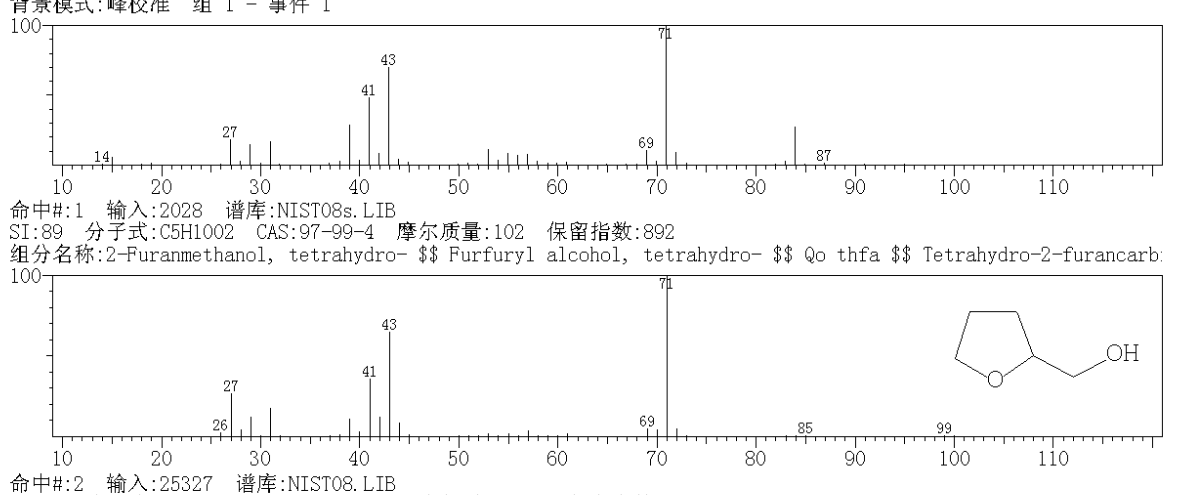


(a) MS spectra of the product


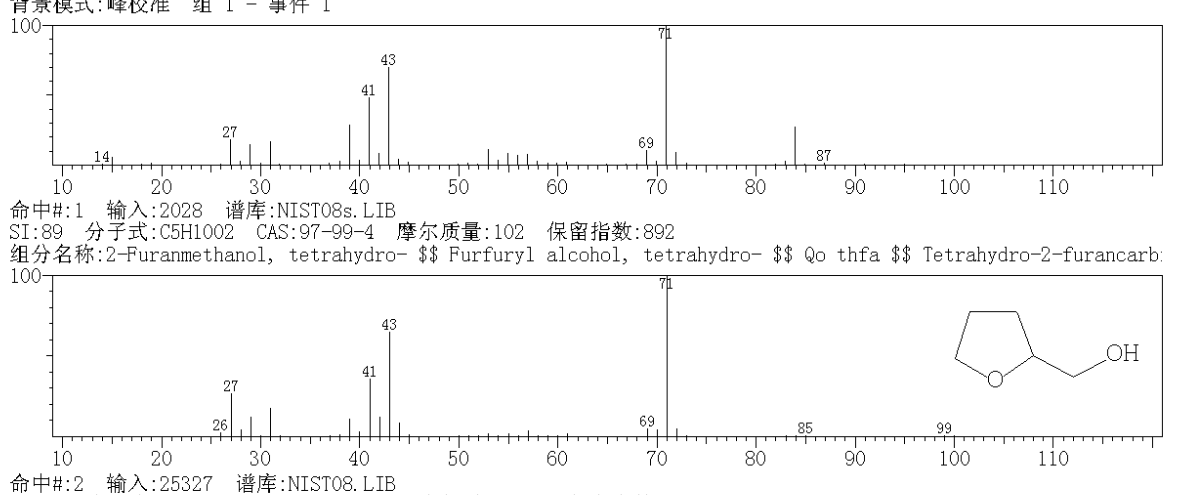


(b) MS spectra of the tetrahydrofurfuryl alcohol

**Figure S17.** The mass spectra of the liquid products of isoprene pyrolysis. (Tetrahydrofurfuryl alcohol)


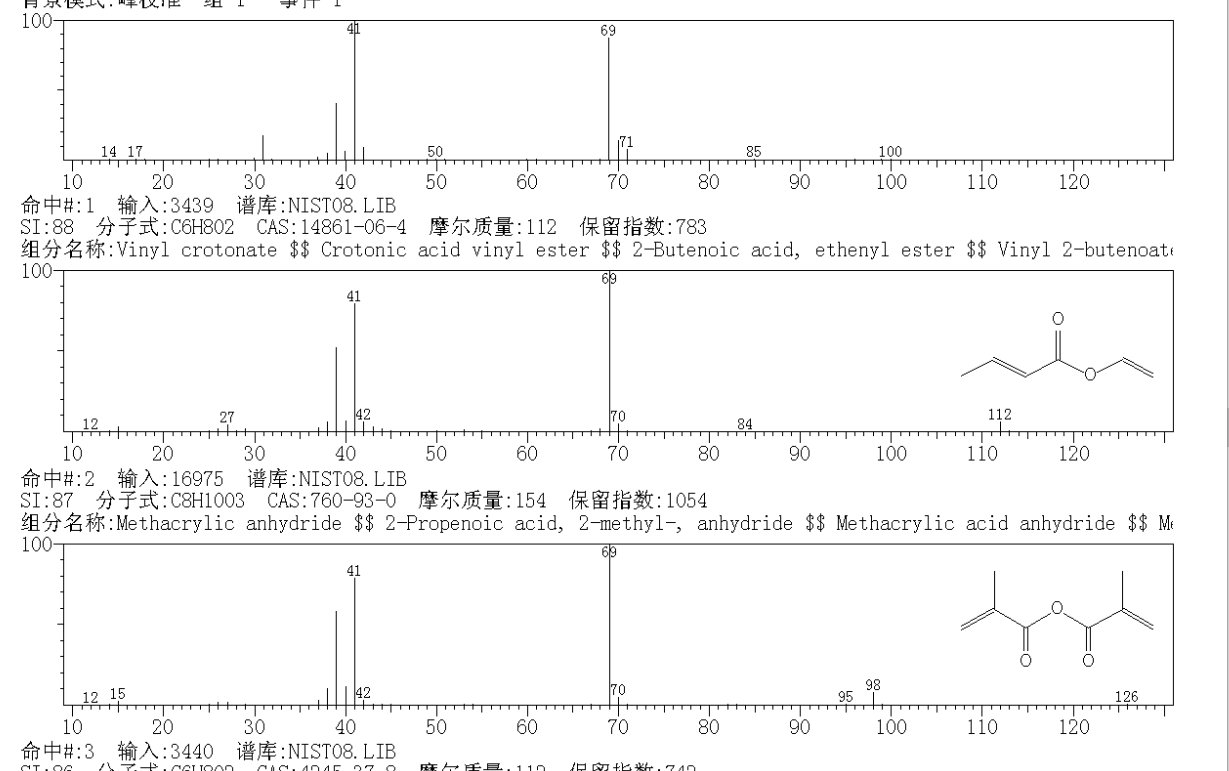


(a) MS spectra of the product


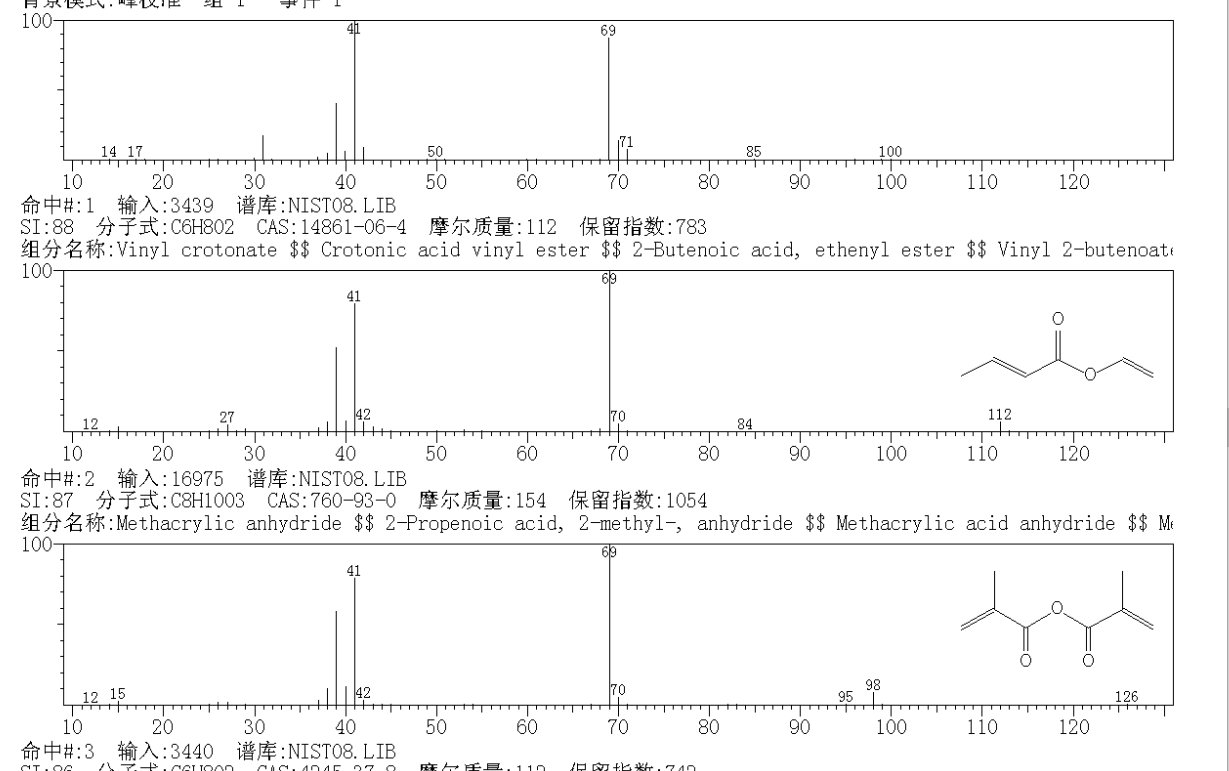


(b) MS spectra of the methacrylic anhydride

**Figure S18.** The mass spectra of the liquid products of isoprene pyrolysis. (Methacrylic anhydride)


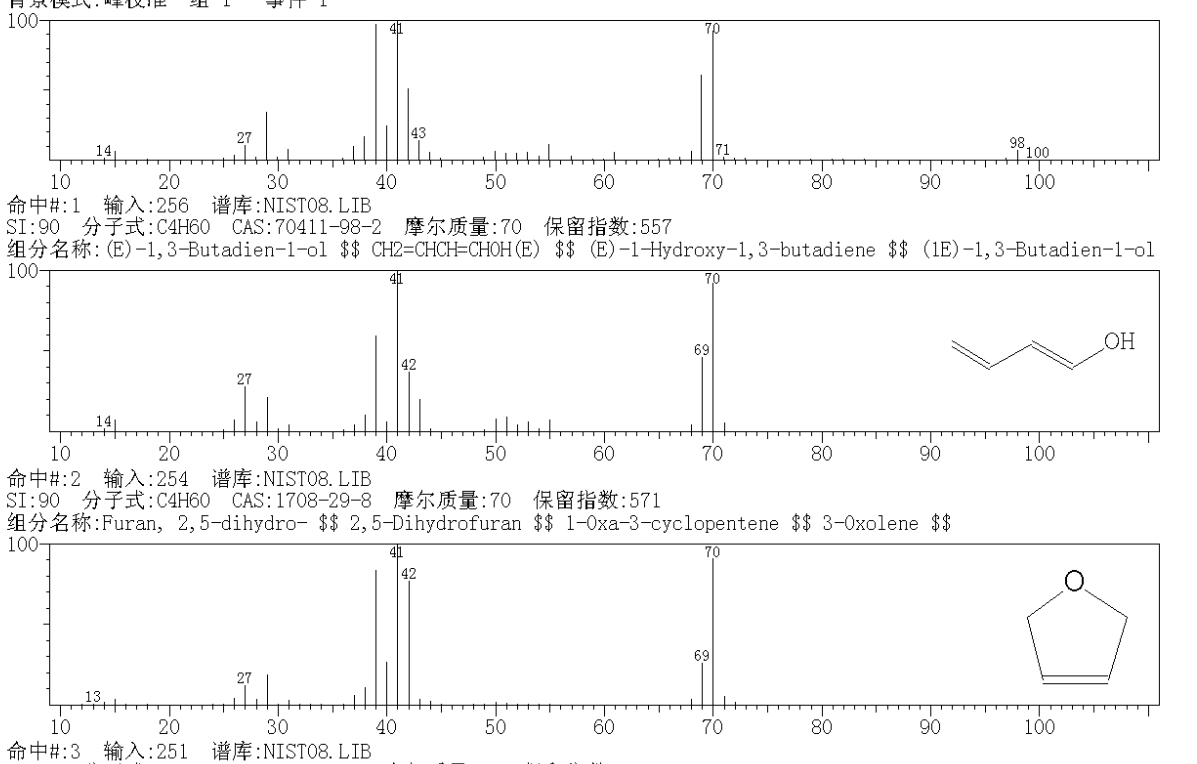


(a) MS spectra of the product


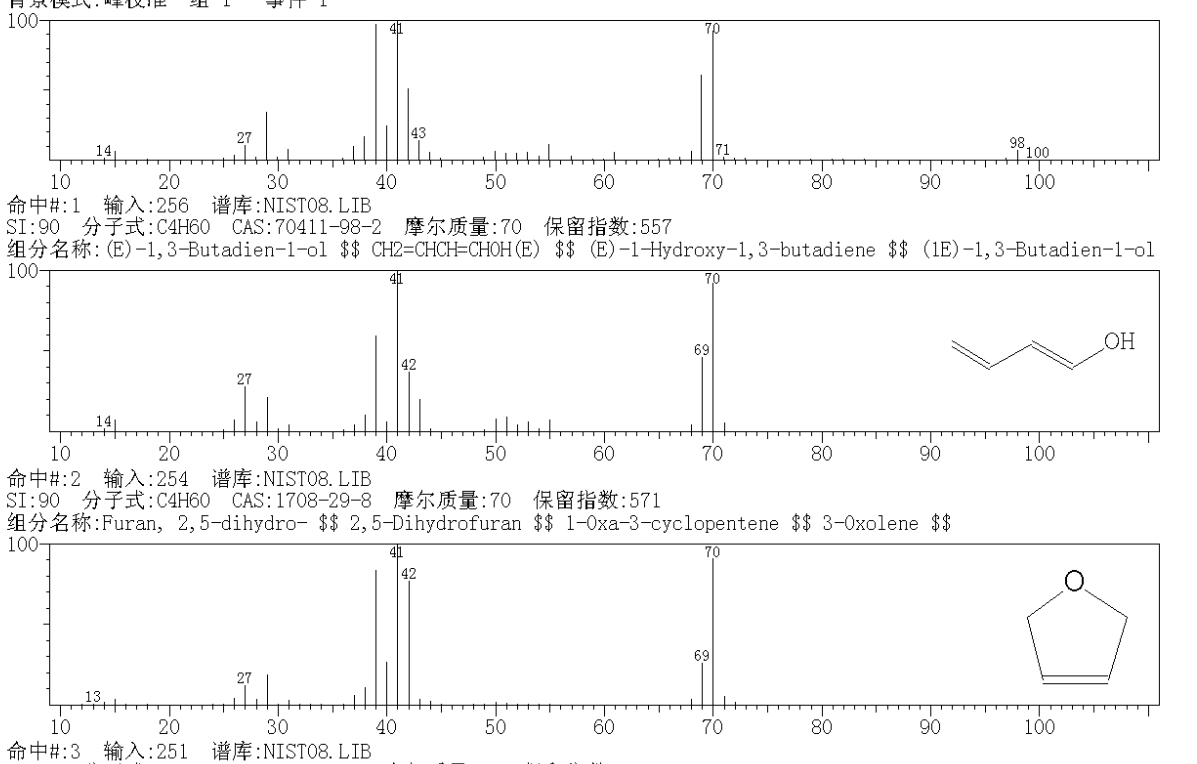


(b) MS spectra of the 2,5-dihydrofuran

**Figure S19.** The mass spectra of the liquid products of isoprene pyrolysis. (2,5-Dihydrofuran)


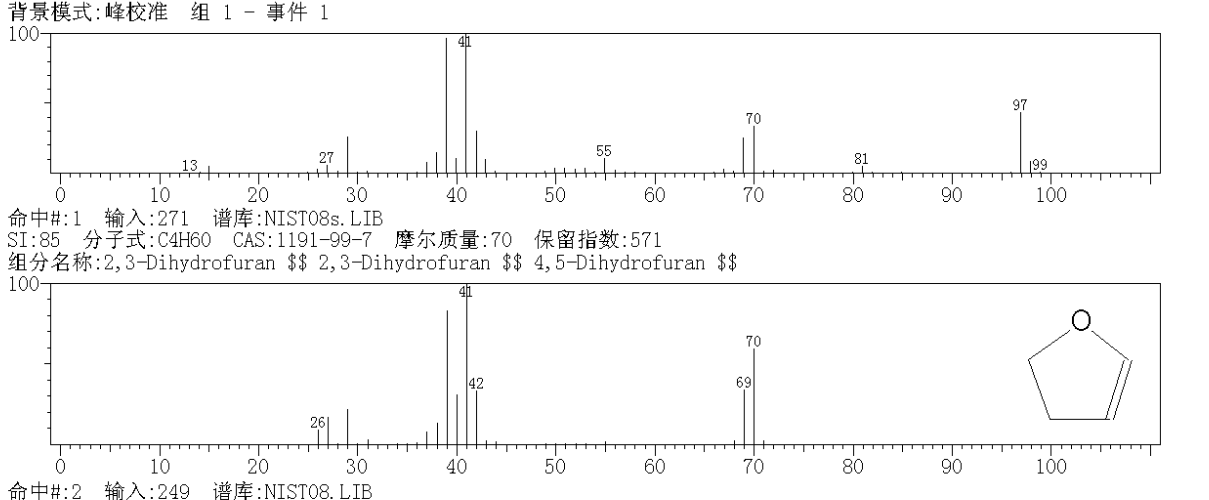


(a) MS spectra of the product


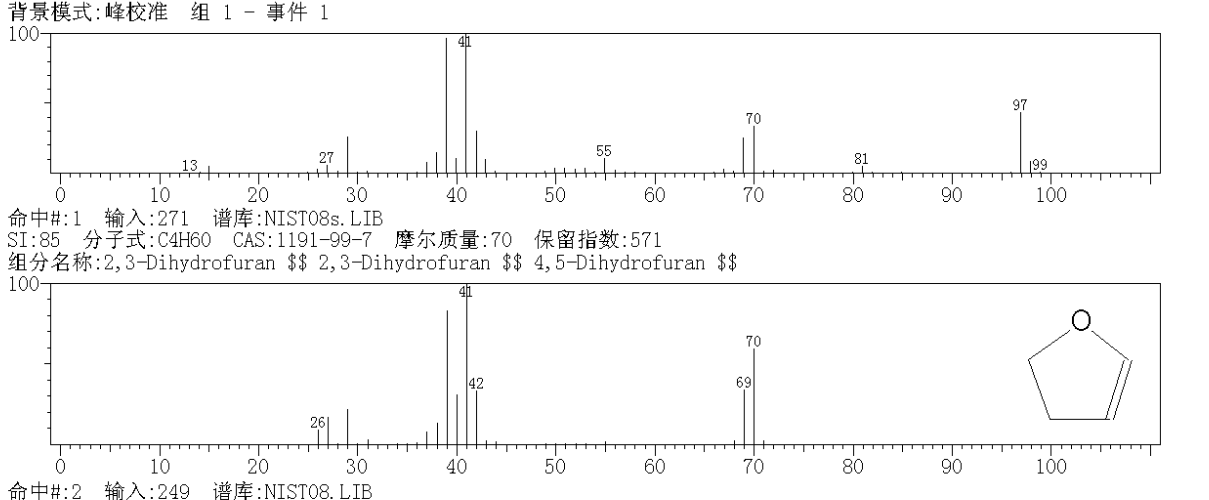


(b) MS spectra of the 2,3-dihydrofuran

**Figure S20.** The mass spectra of the liquid products of isoprene pyrolysis. (2,3-Dihydrofuran)


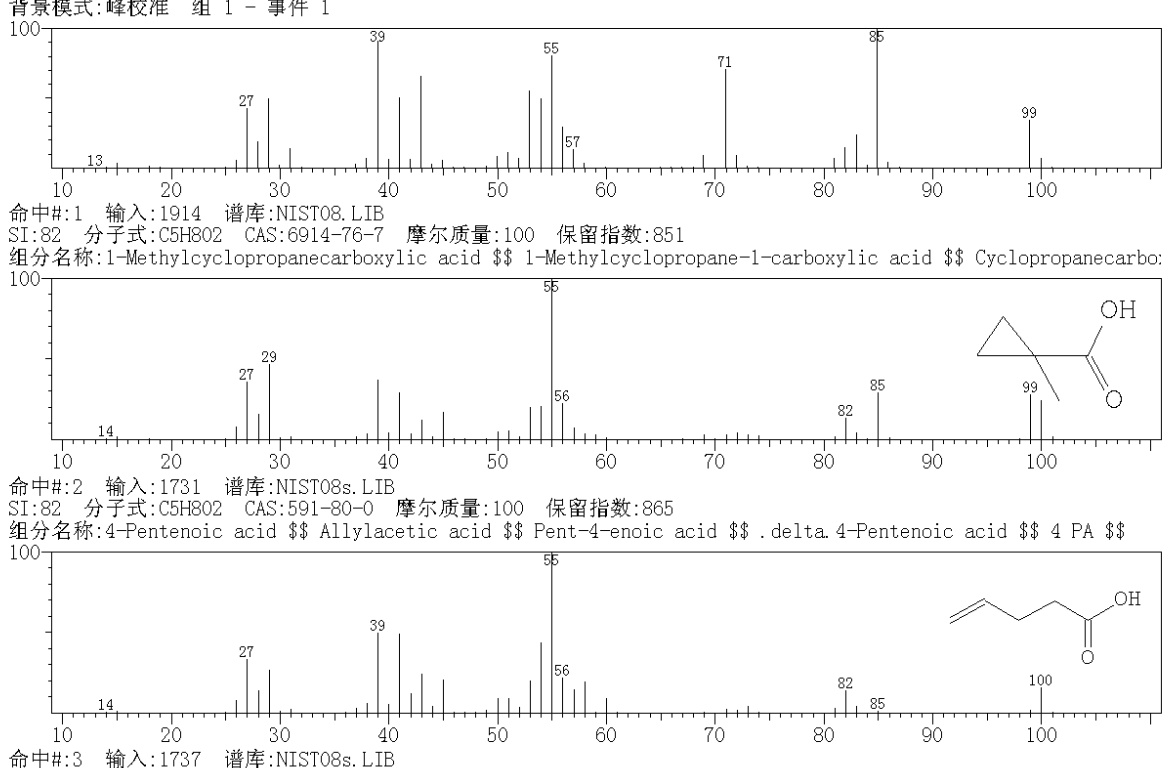


(a) MS spectra of the product


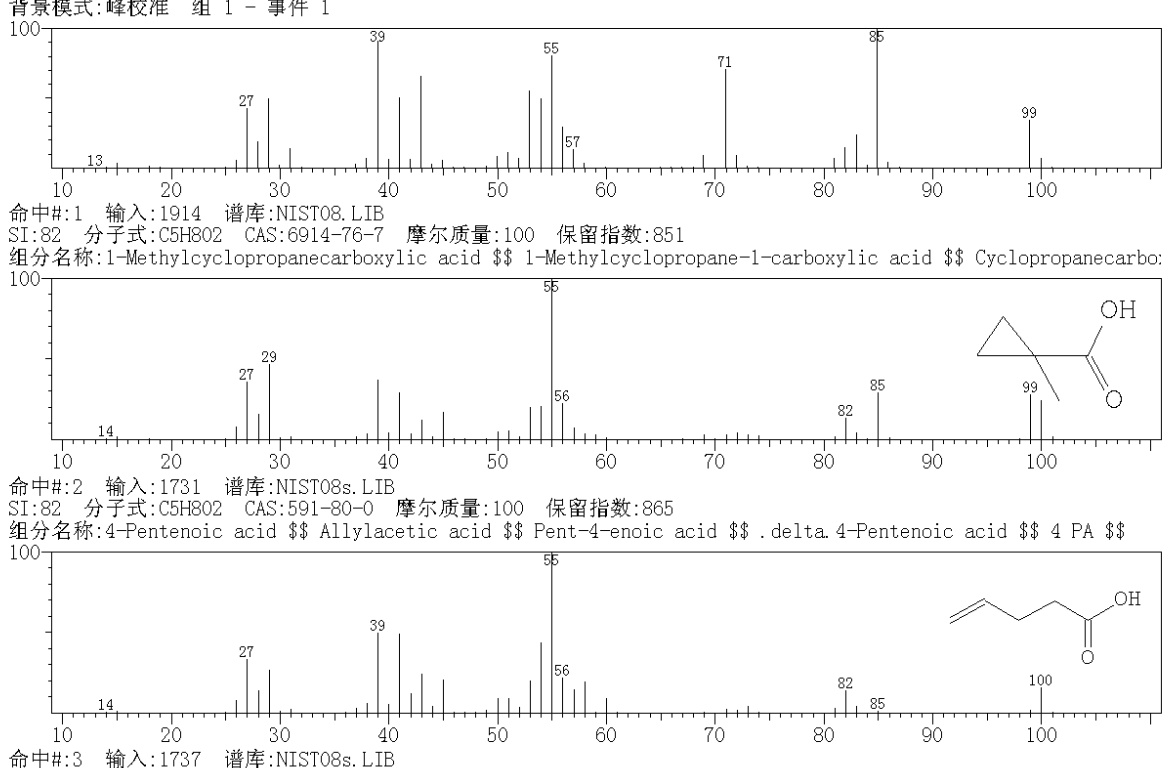


(b) MS spectra of the 2-butenoic acid

**Figure S21.** The mass spectra of the liquid products of isoprene pyrolysis. (2-Butenoic acid)


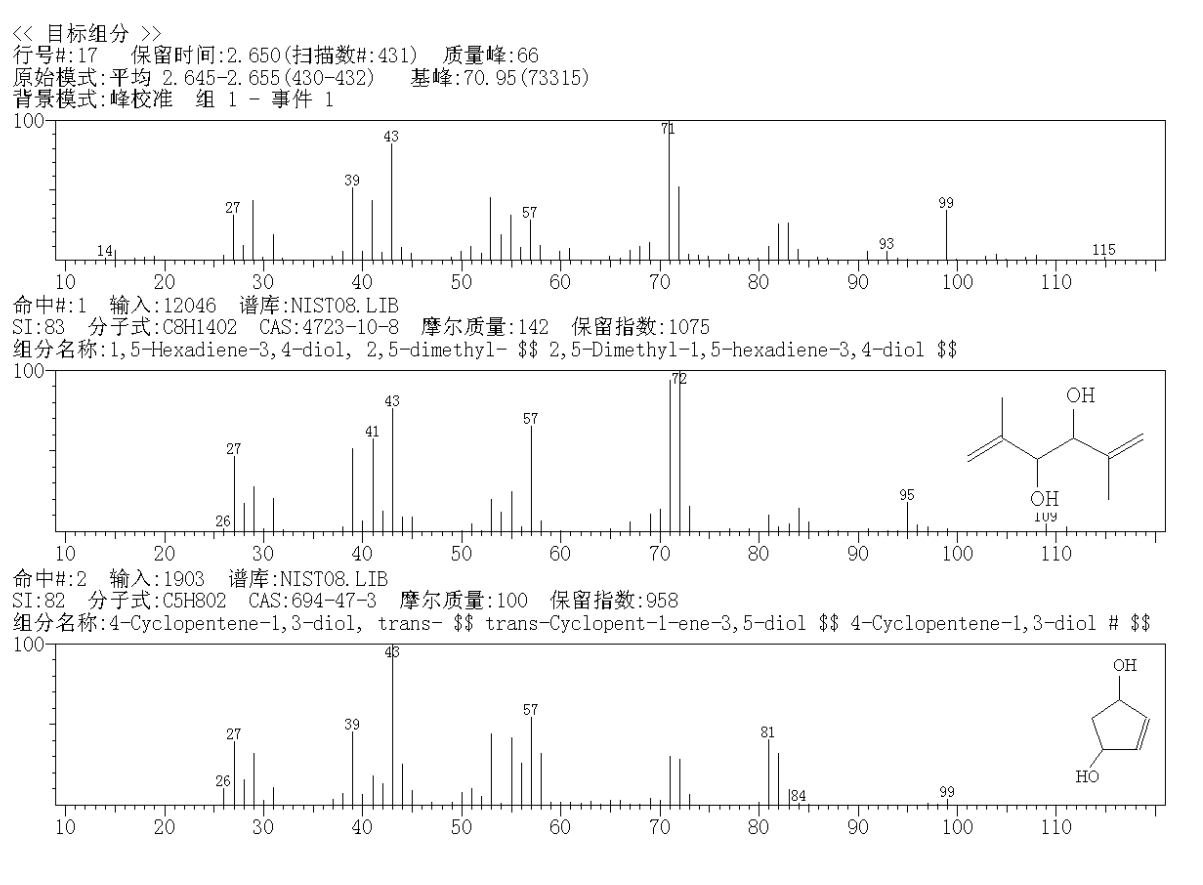


(a) MS spectra of the product


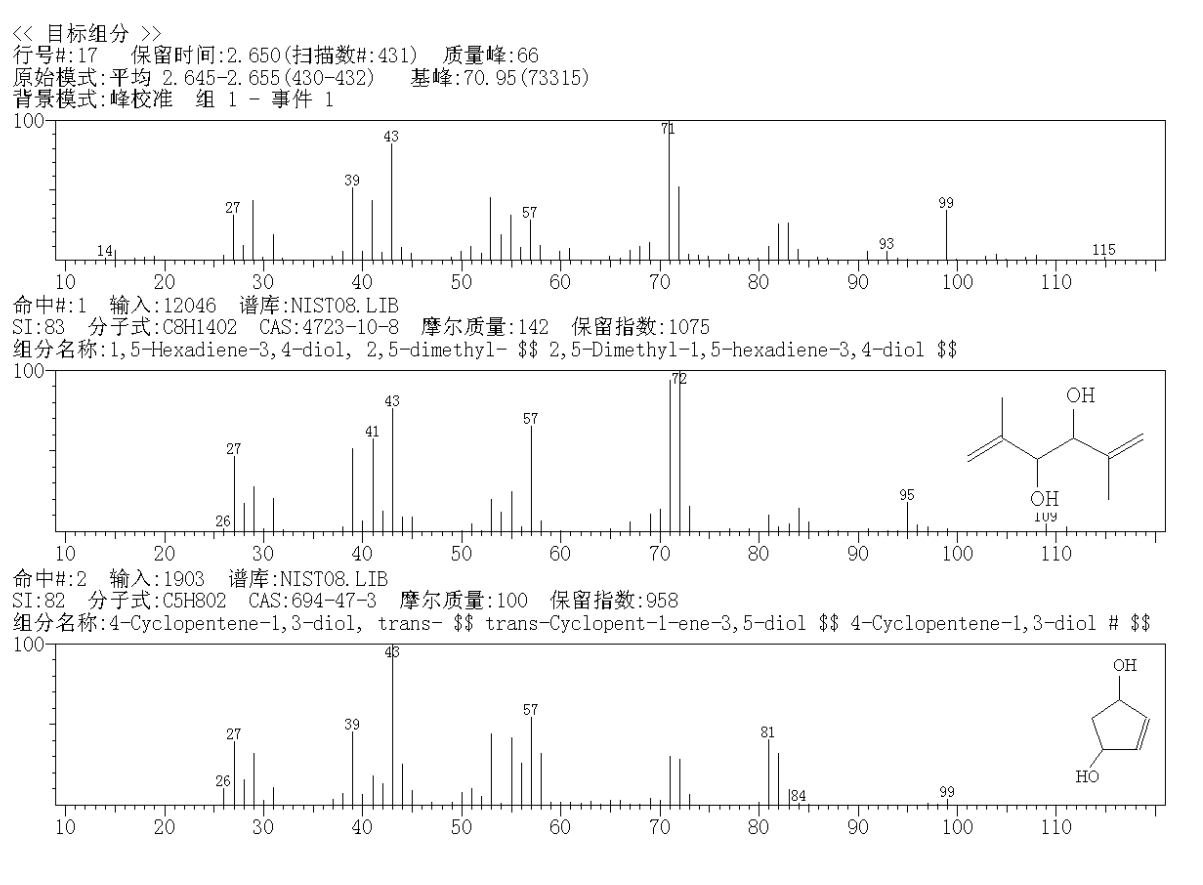


(b) MS spectra of the (1R,3S)-4-cyclopentene-1,3-diol

**Figure S22.** The mass spectra of the liquid products of isoprene pyrolysis. ((1R,3S)-4-cyclopentene-1,3-diol)


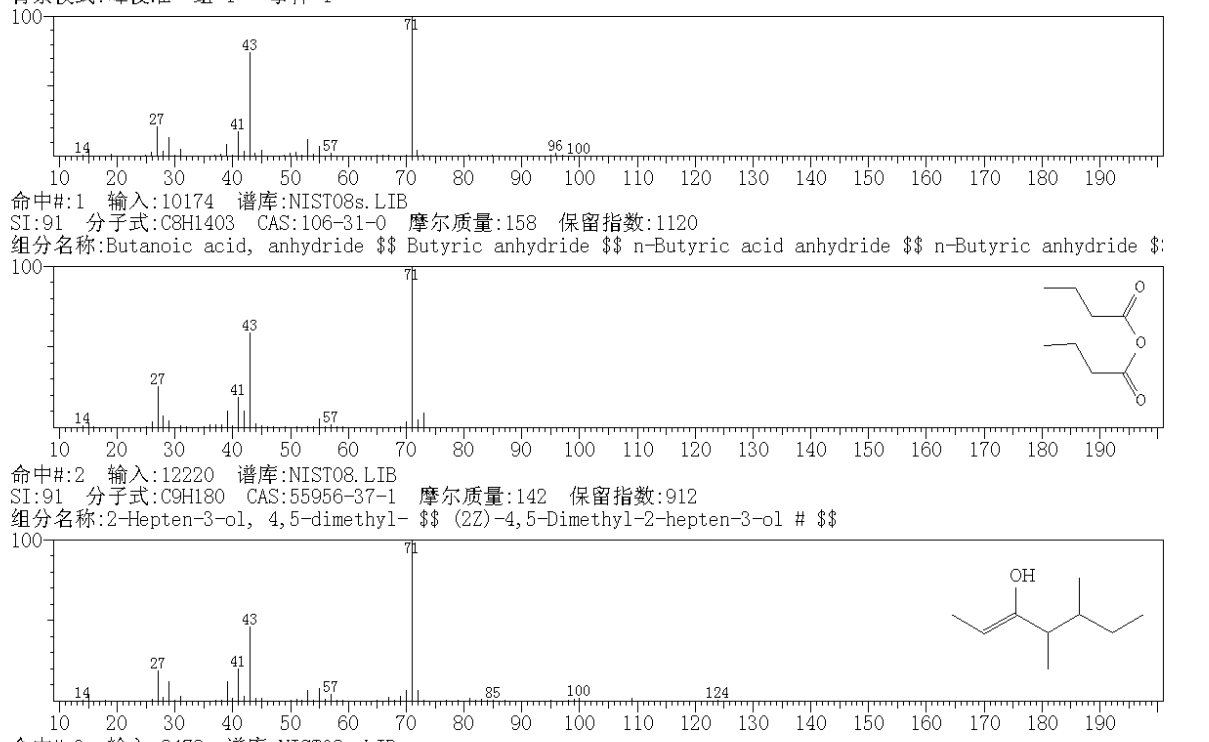


(a) MS spectra of the product


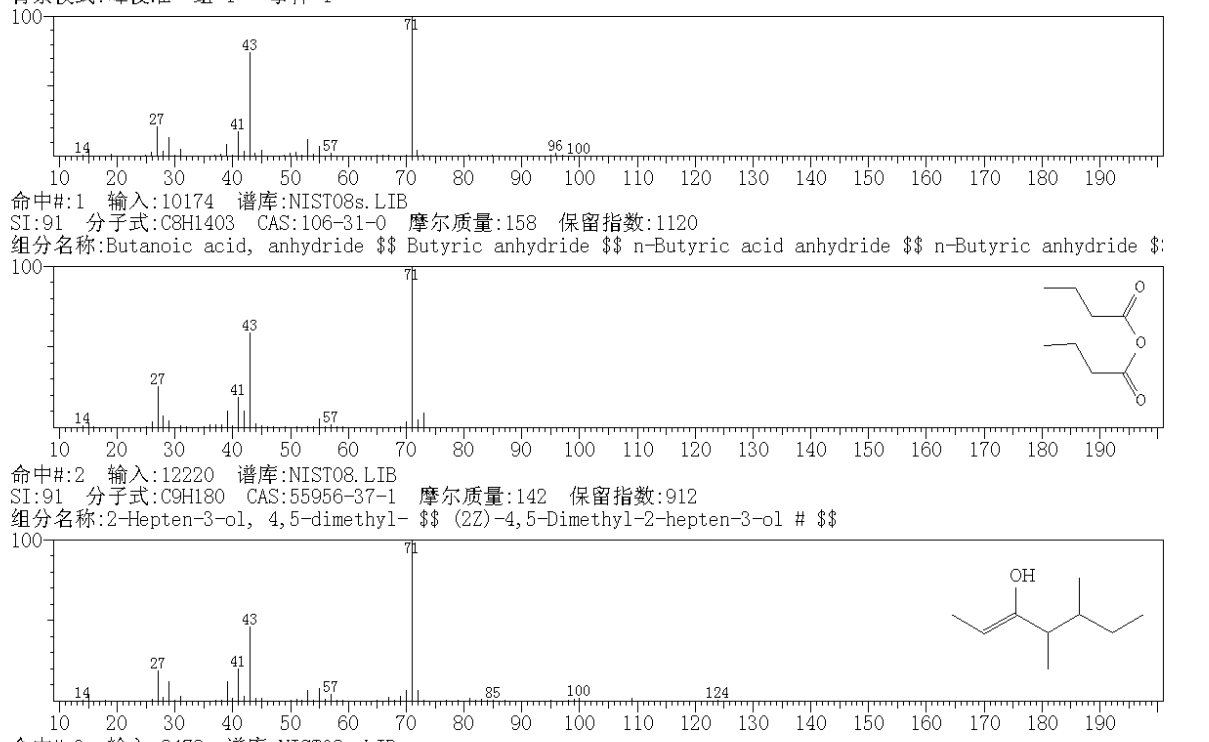


(b) MS spectra of the 4,5-dimethyl-2-hepten-3-ol

**Figure S23.** The mass spectra of the liquid products of isoprene pyrolysis. (4,5-Dimethyl-2-hepten-3-ol)


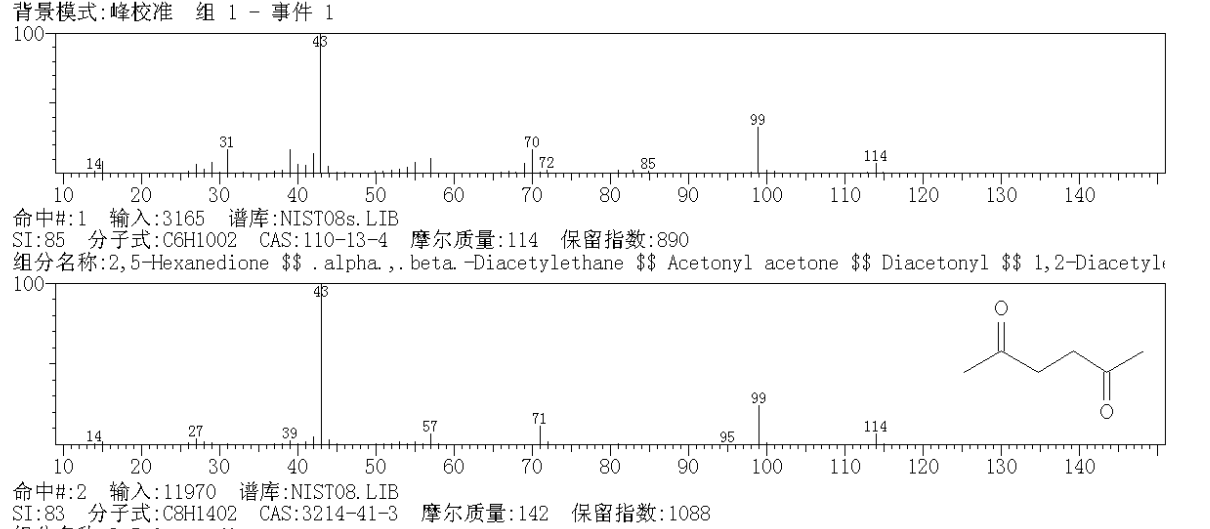


(a) MS spectra of the product


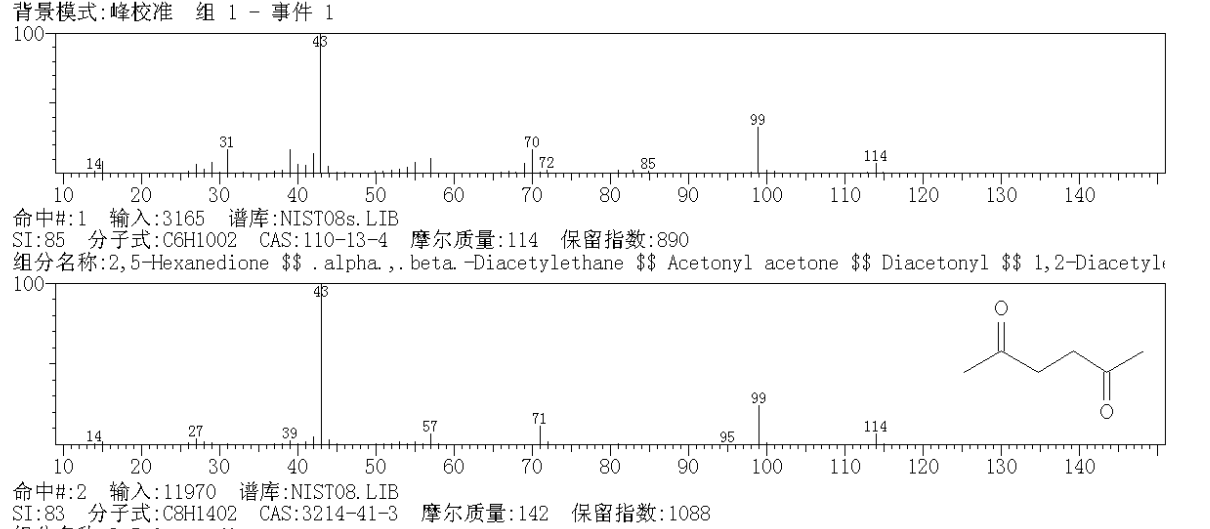


(b) MS spectra of the 2,5-hexanedione

**Figure S24.** The mass spectra of the liquid products of isoprene pyrolysis. (2,5-Hexanedione)


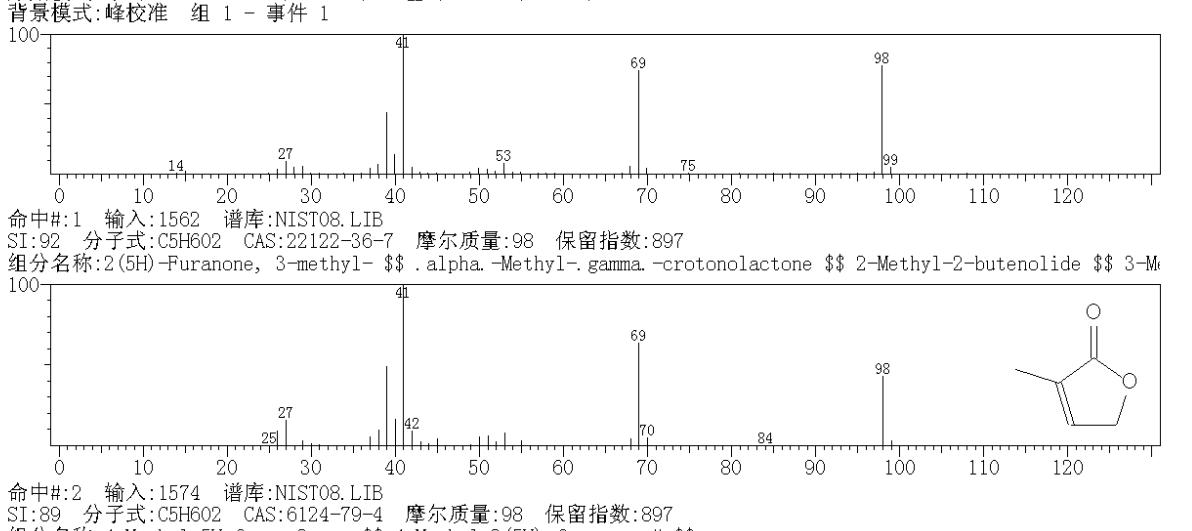


(a) MS spectra of the product


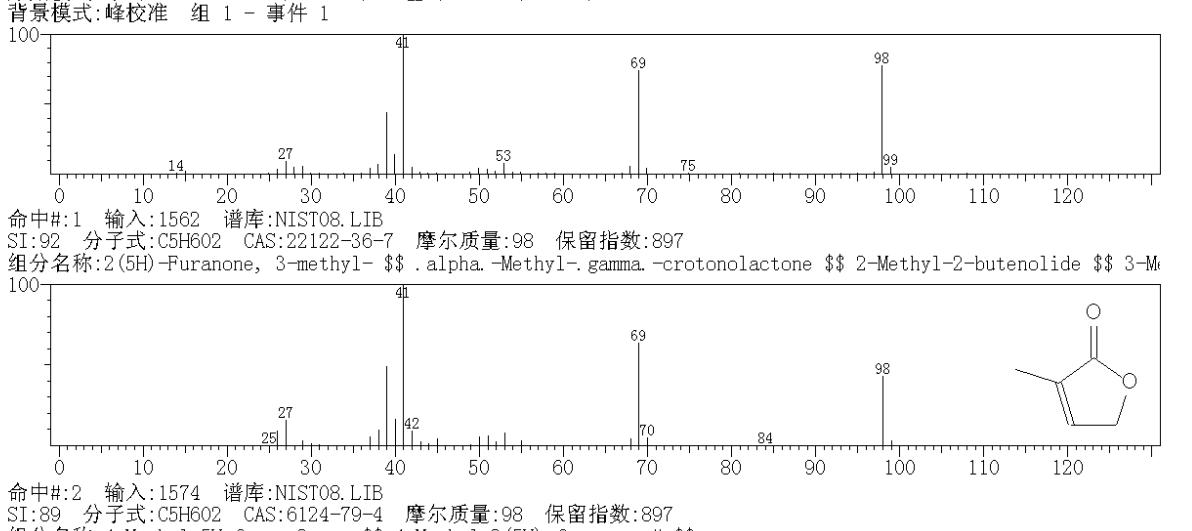


(b) MS spectra of the 3-methyl-2(5*H*)-furanone

**Figure S25.** The mass spectra of the liquid products of isoprene pyrolysis. (3-Methyl-2(5*H*)-furanone)


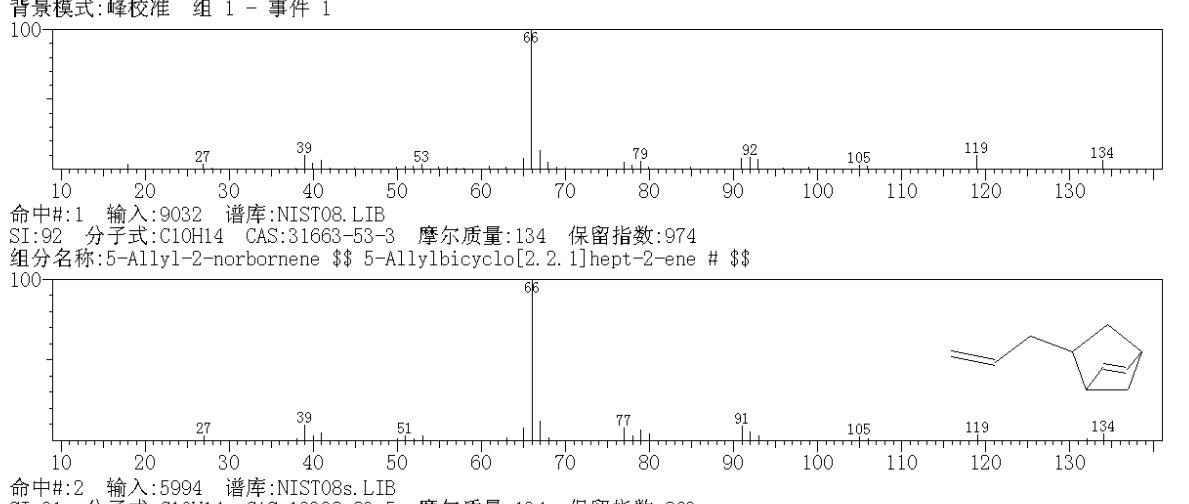


(a) MS spectra of the product


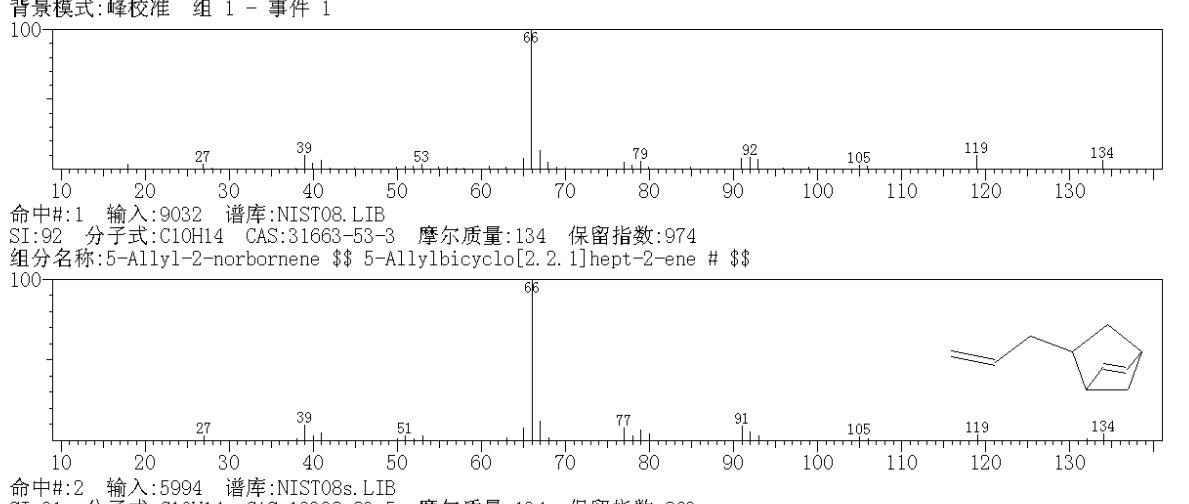


(b) MS spectra of the tricyclo[5.2.1.0^2,6^]dec-8-ene

**Figure S26.** The mass spectra of the liquid products of isoprene pyrolysis. (Tricyclo[5.2.1.0^2,6^]dec-8-ene)


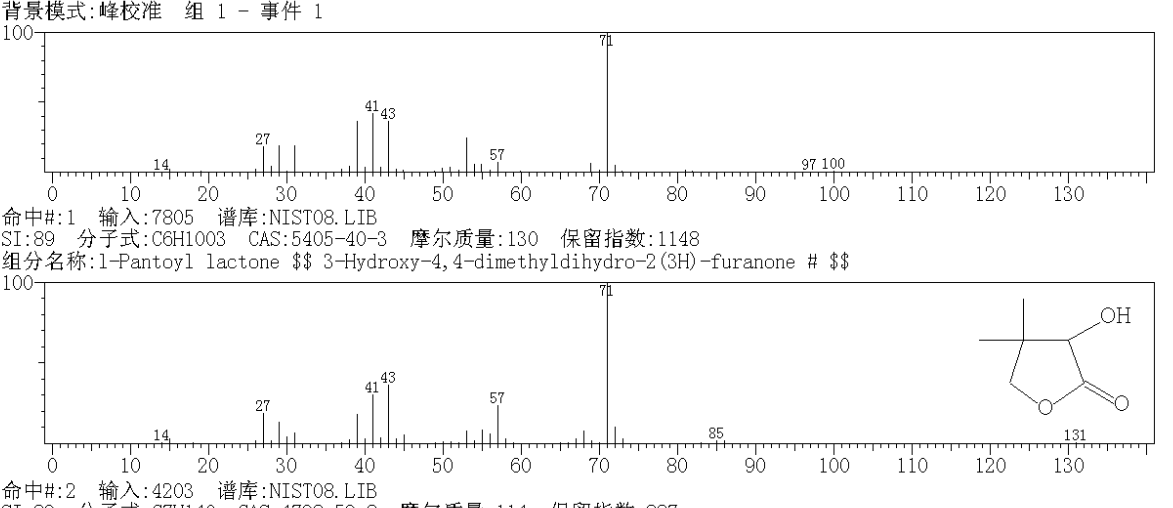


(a) MS spectra of the product


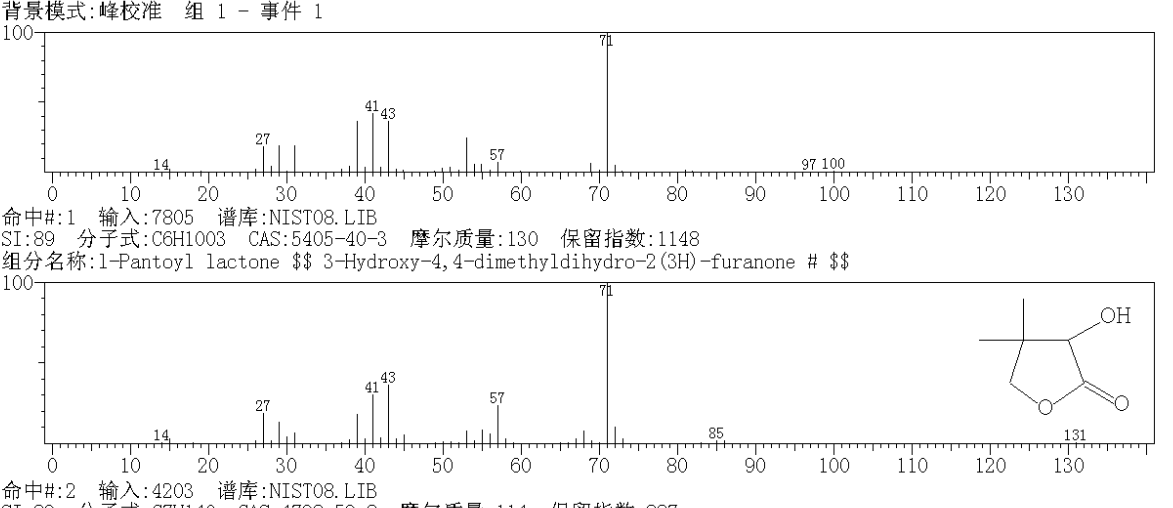


(b) MS spectra of the (+)-pantolactone

**Figure S27.** The mass spectra of the liquid products of isoprene pyrolysis. ((+)-Pantolactone)


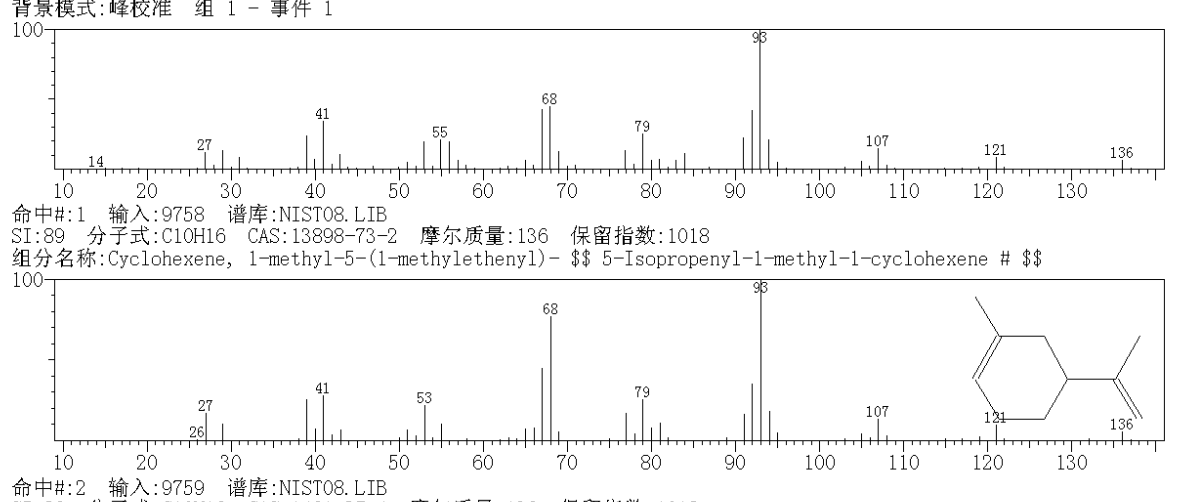


(a) MS spectra of the product


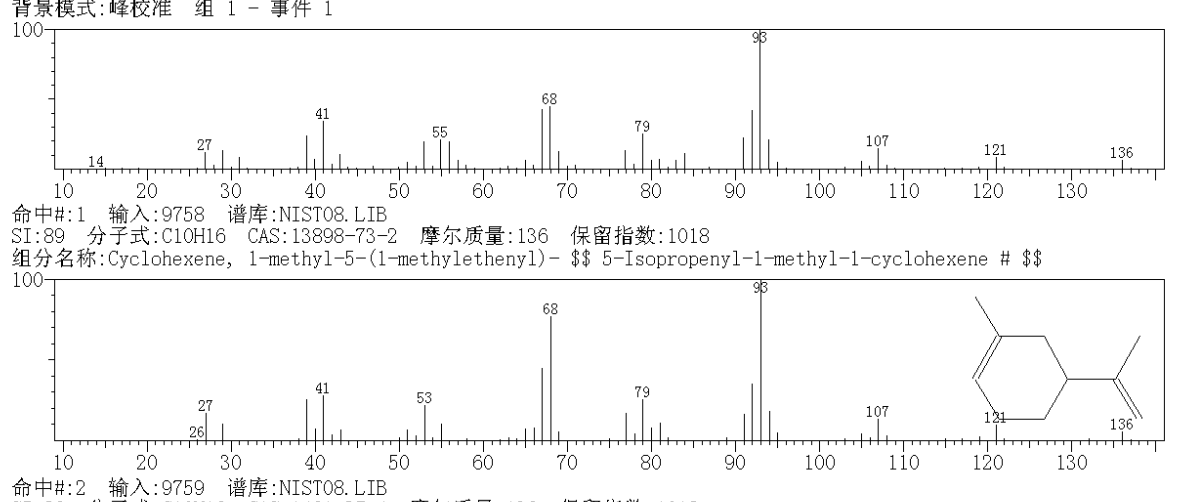


(b) MS spectra of the sylvestrene

**Figure S28.** The mass spectra of the liquid products of isoprene pyrolysis. (Sylvestrene)


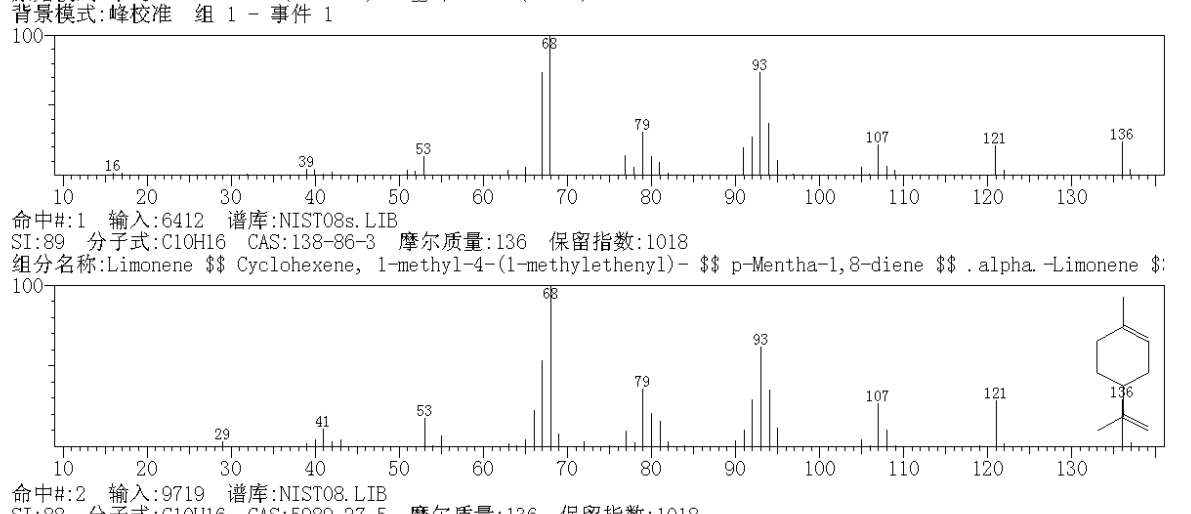


(a) MS spectra of the product


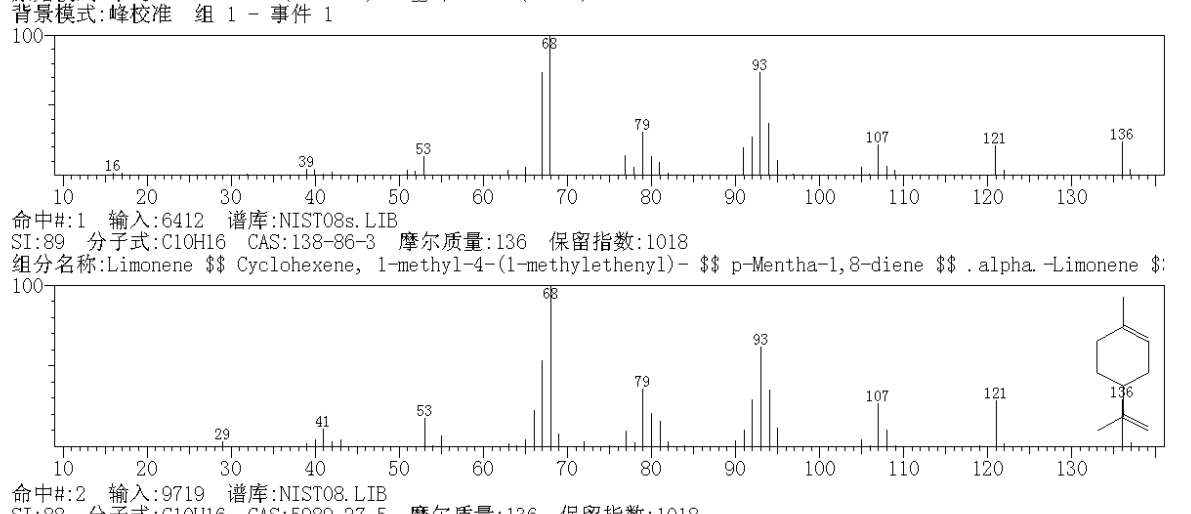


(b) MS spectra of the limonene

**Figure S29.** The mass spectra of the liquid products of isoprene pyrolysis. (Limonene)


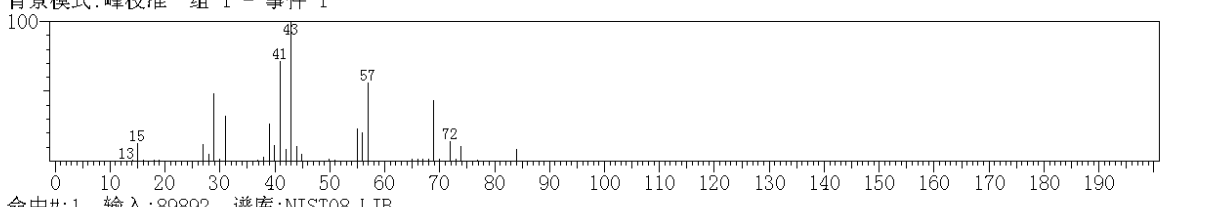


(a) MS spectra of the product


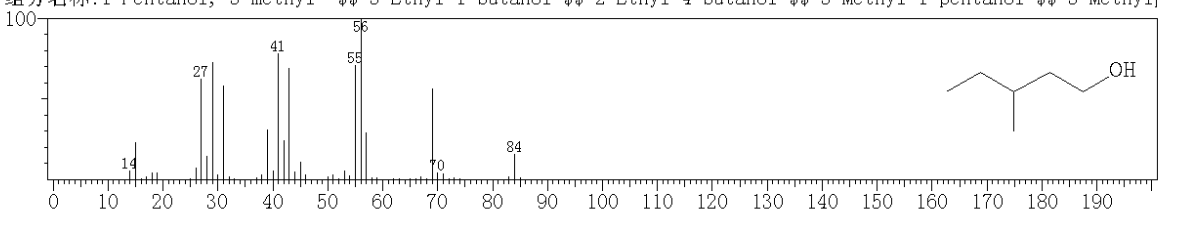


(b) MS spectra of the 3-methyl-1-pentanol

**Figure S30.** The mass spectra of the liquid products of isoprene pyrolysis. (3-Methyl-1-pentanol)


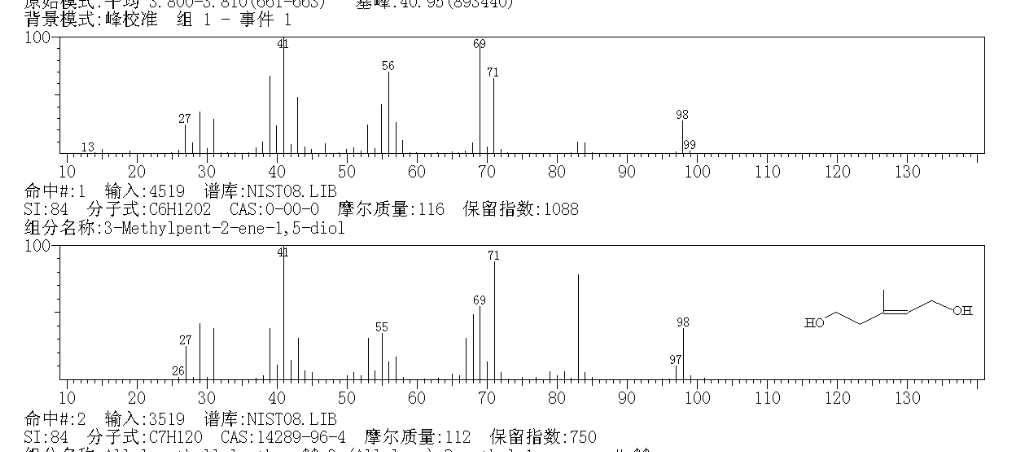


(a) MS spectra of the product


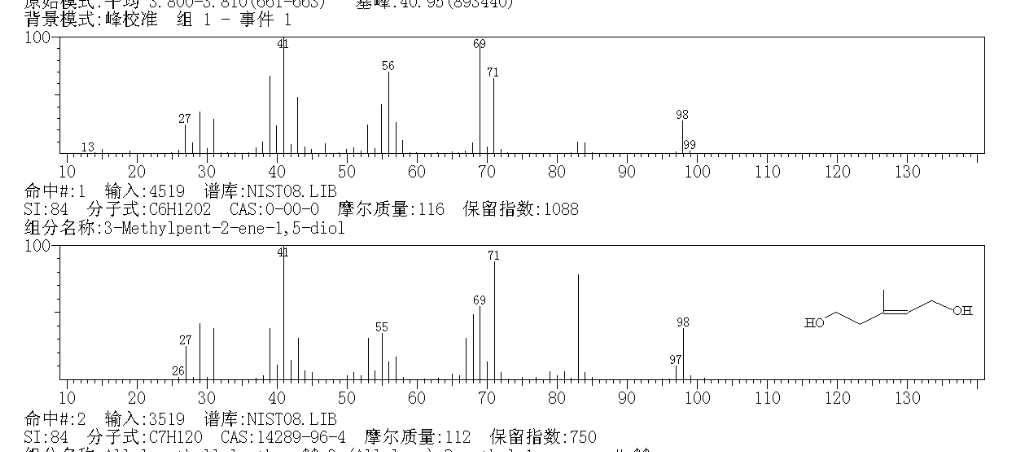


(b) MS spectra of the 3-methyl-2-pentene-1,5-diol

**Figure S31.** The mass spectra of the liquid products of isoprene pyrolysis. (3-Methyl-2-pentene-1,5-diol)


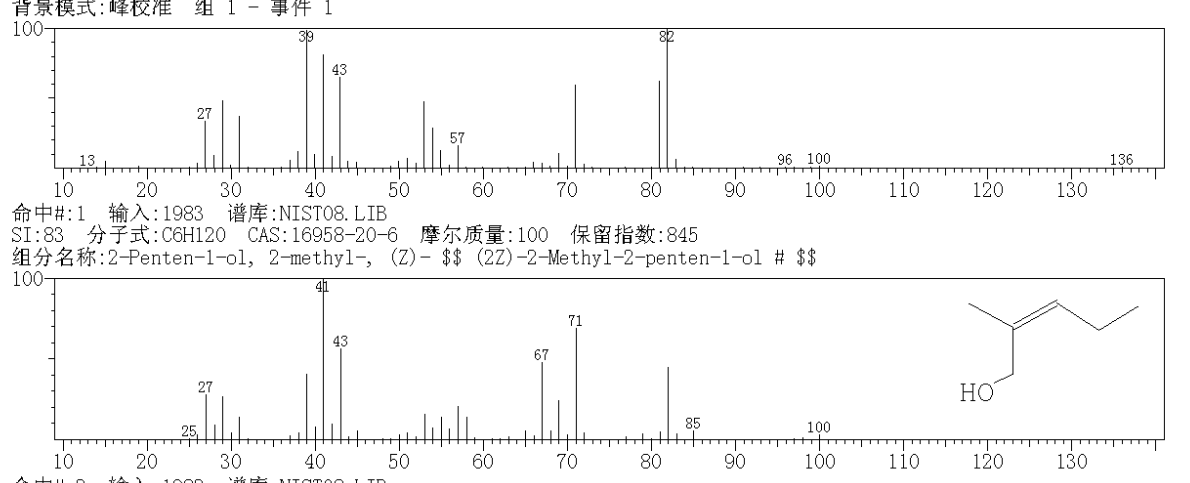


(a) MS spectra of the product


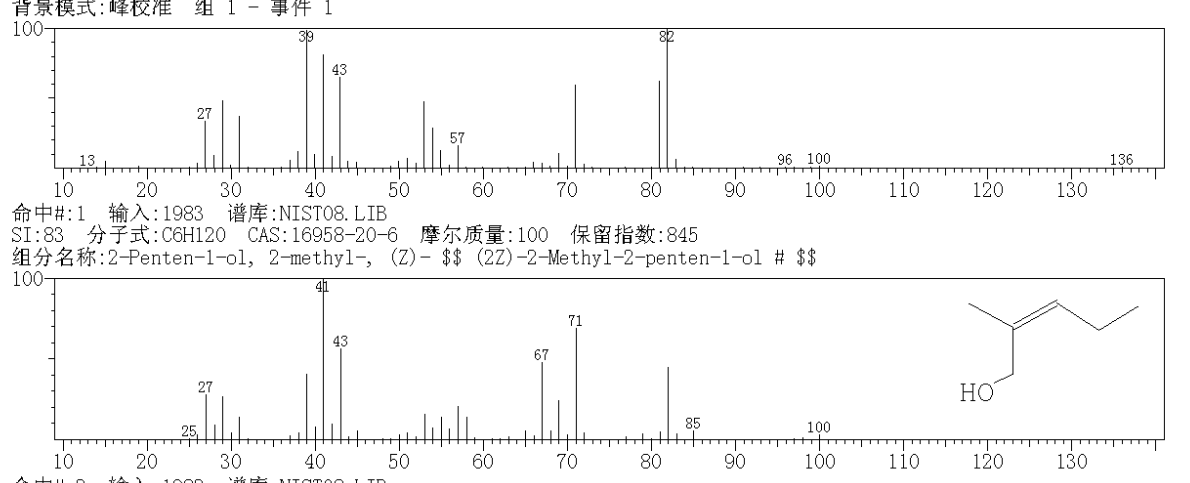


(b) MS spectra of the (2*Z*)-2-methyl-2-penten-1-ol

**Figure S32.** The mass spectra of the liquid products of isoprene pyrolysis. ((2*Z*)-2-Methyl-2-penten-1-ol)


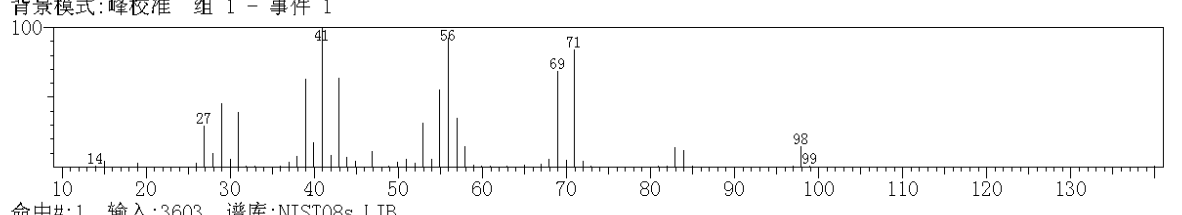


(a) MS spectra of the product


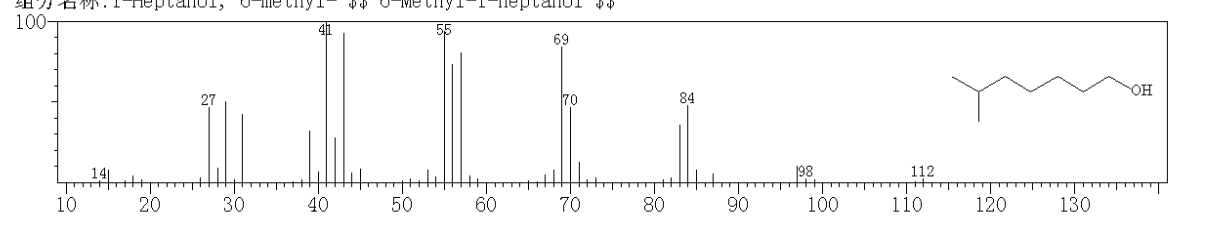


(b) MS spectra of the 6-methyl-1-heptanol

**Figure S33.** The mass spectra of the liquid products of isoprene pyrolysis. (6-Methyl-1-heptanol)


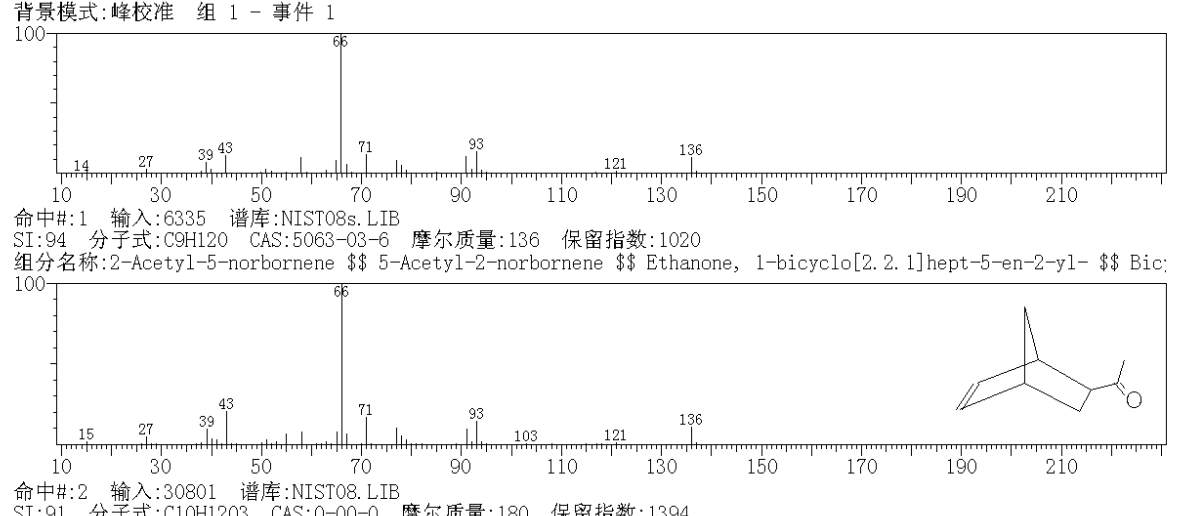


(a) MS spectra of the product


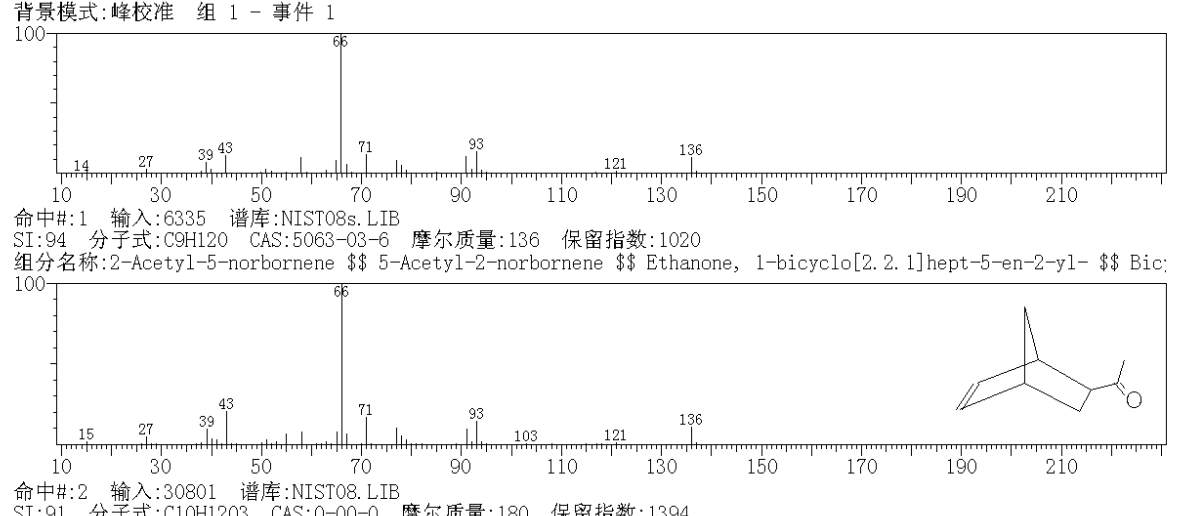


(b) MS spectra of the 1-bicyclo[2.2.1]hept-5-en-2-ylethanone

**Figure S34.** The mass spectra of the liquid products of isoprene pyrolysis. (1-Bicyclo[2.2.1]hept-5-en-2-ylethanone)


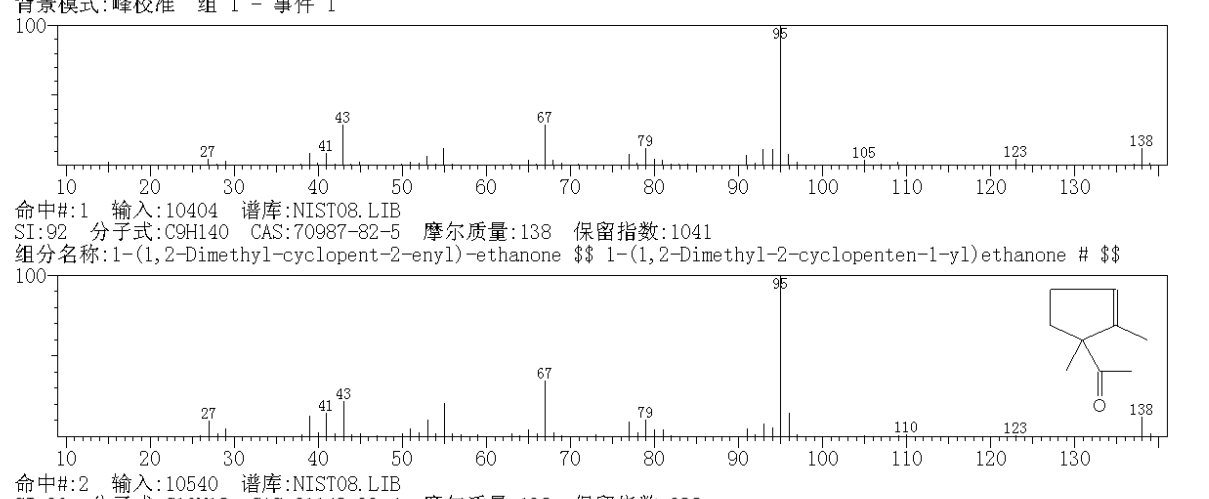


(a) MS spectra of the product


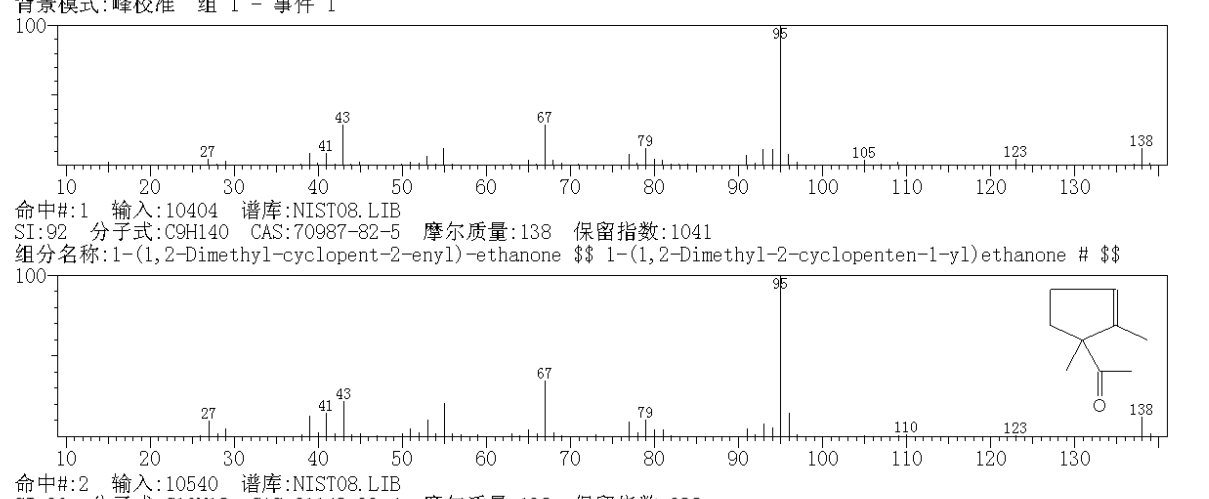


(b) MS spectra of the 1-(1,2-dimethyl-2-cyclopenten-1-yl)ethanone

**Figure S35.** The mass spectra of the liquid products of isoprene pyrolysis. (1-(1,2-dimethyl-2-cyclopenten-1-yl)ethanone)


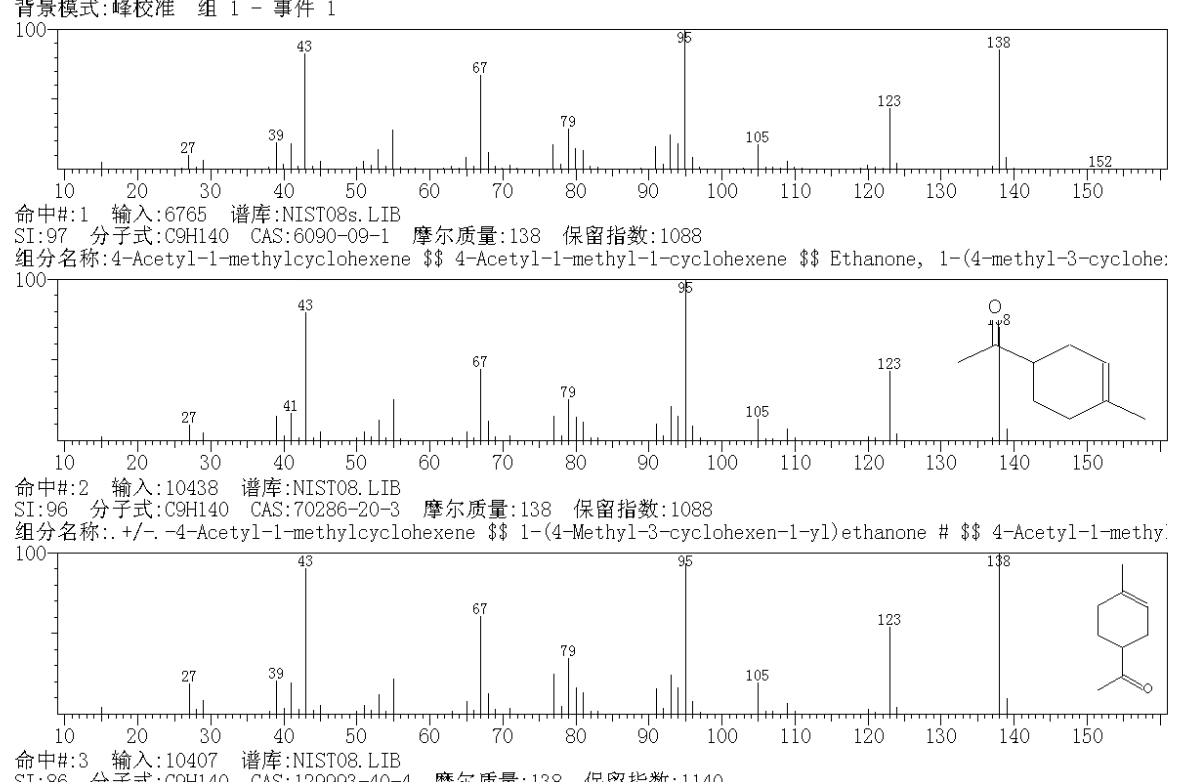


(a) MS spectra of the product


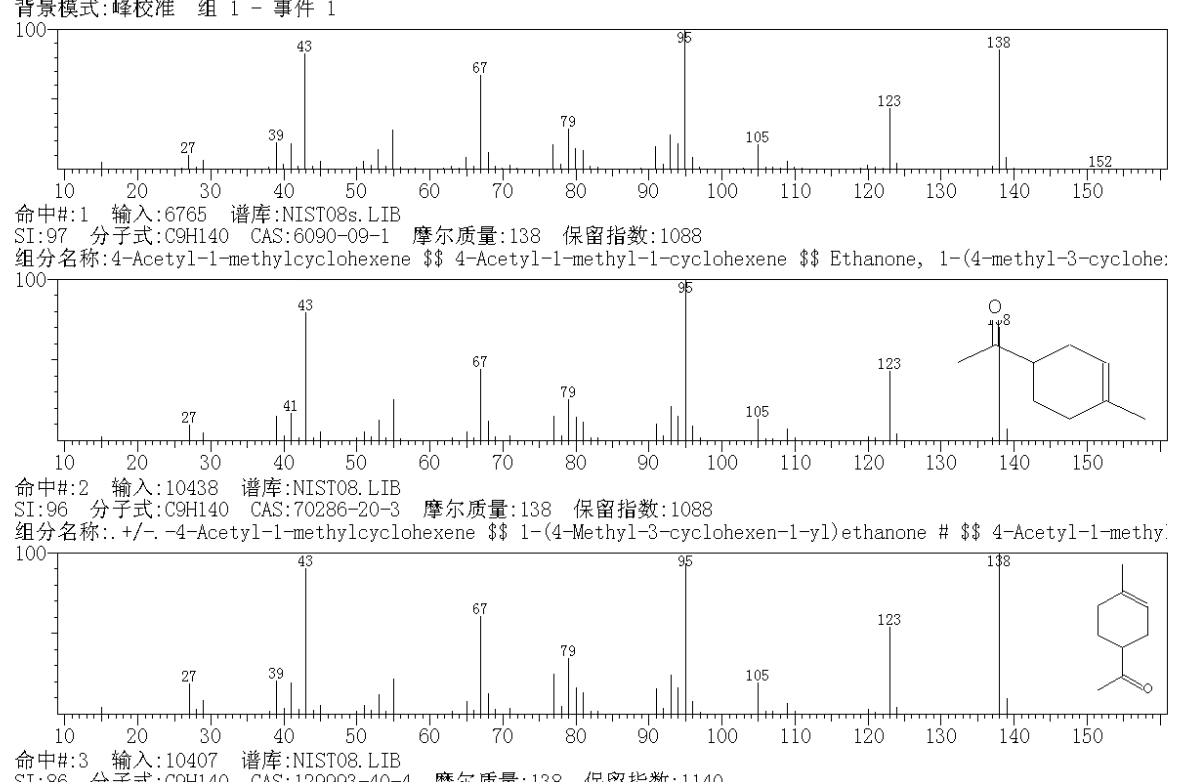


(b) MS spectra of the 4-acetyl-1-methylcyclohexene

**Figure S36.** The mass spectra of the liquid products of isoprene pyrolysis. (4-Acetyl-1-methylcyclohexene)


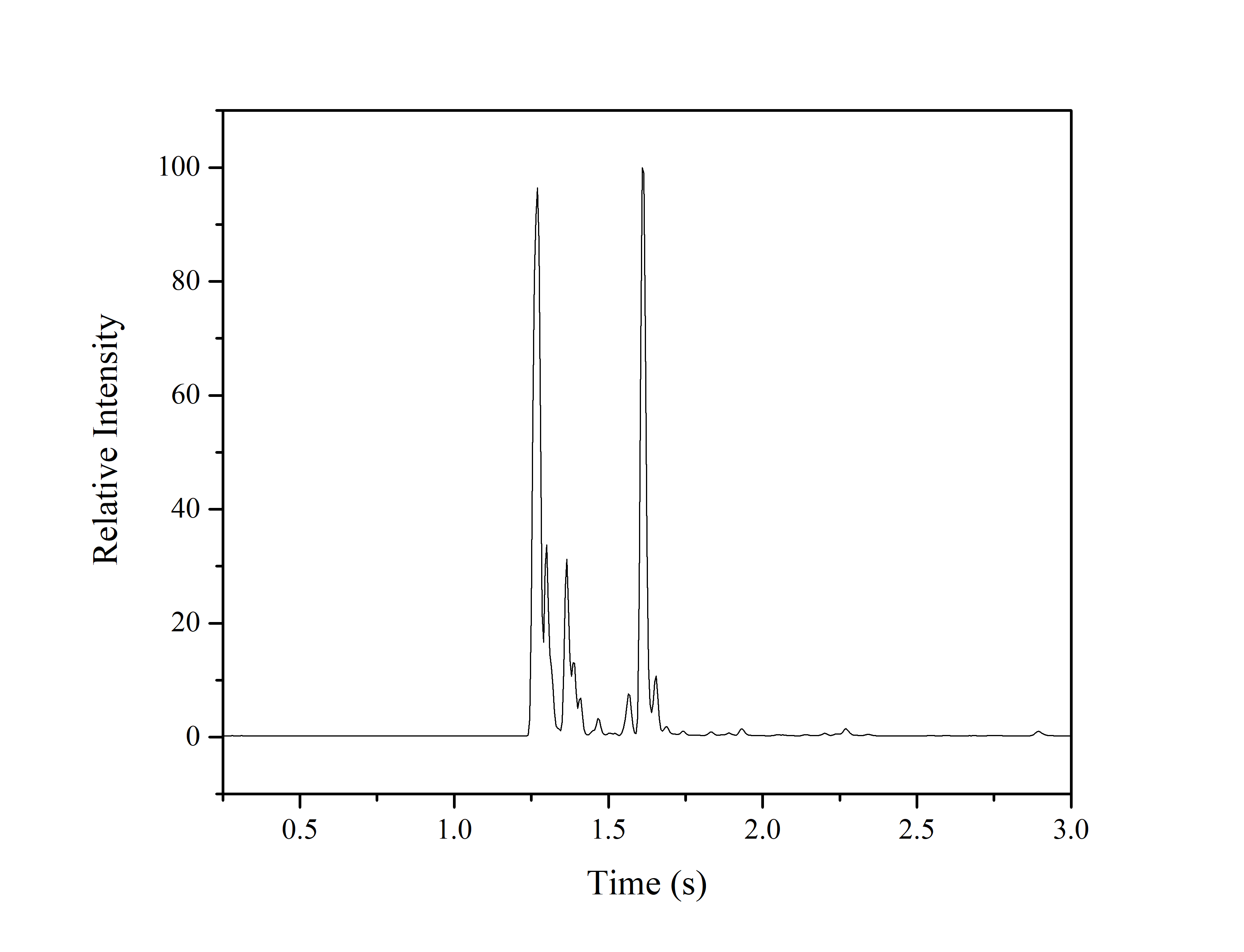


**Figure S37.** The Total ion chromatography of the gas products of isoprene after the explosion.


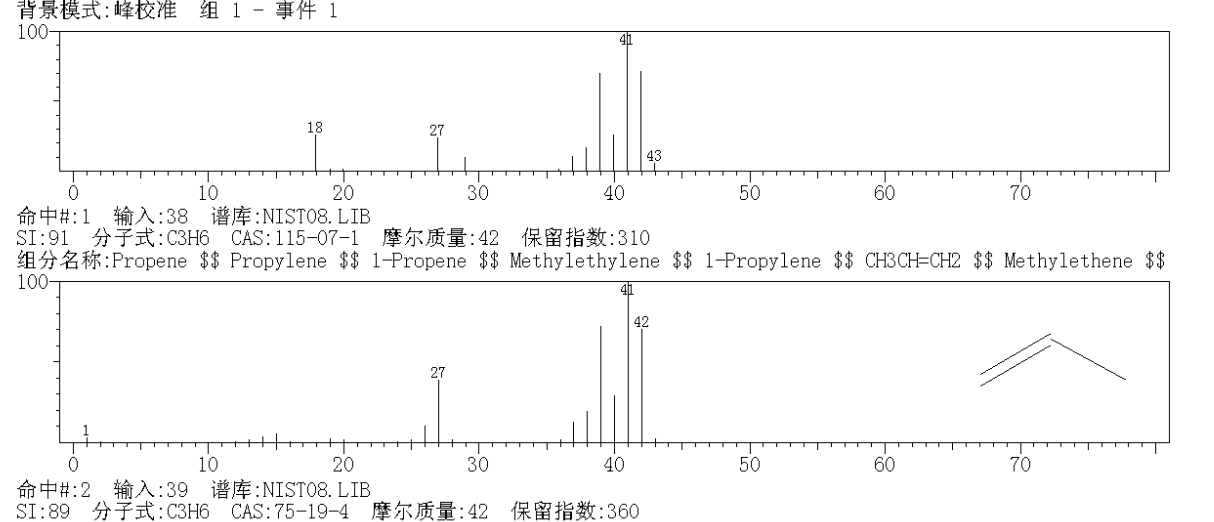


(a) MS spectra of the product


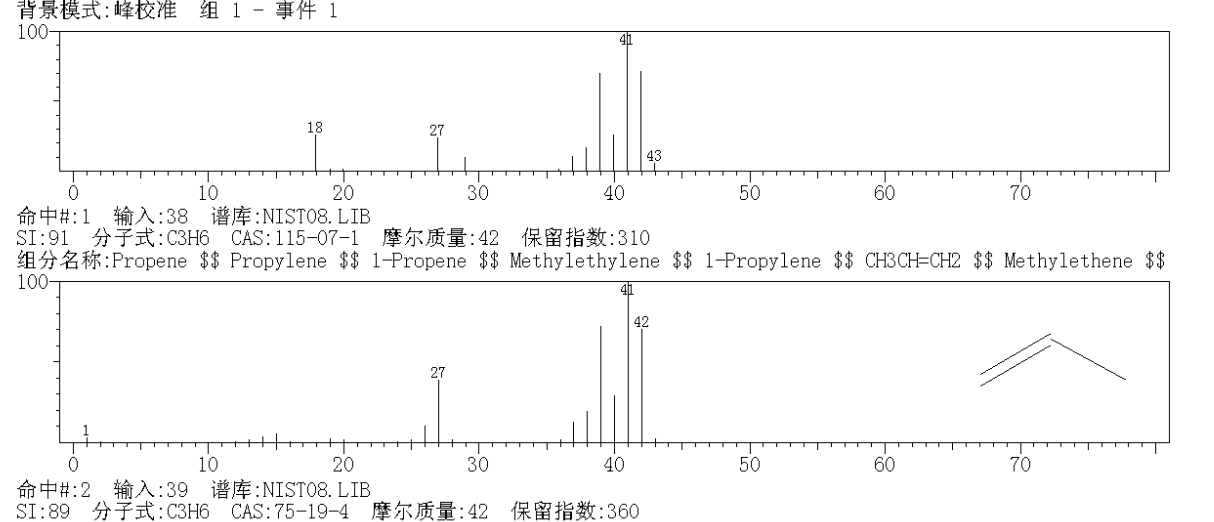


(b) MS spectra of the propene

**Figure S38.** The mass spectra of the gas products of isoprene after the explosion. (Propene)


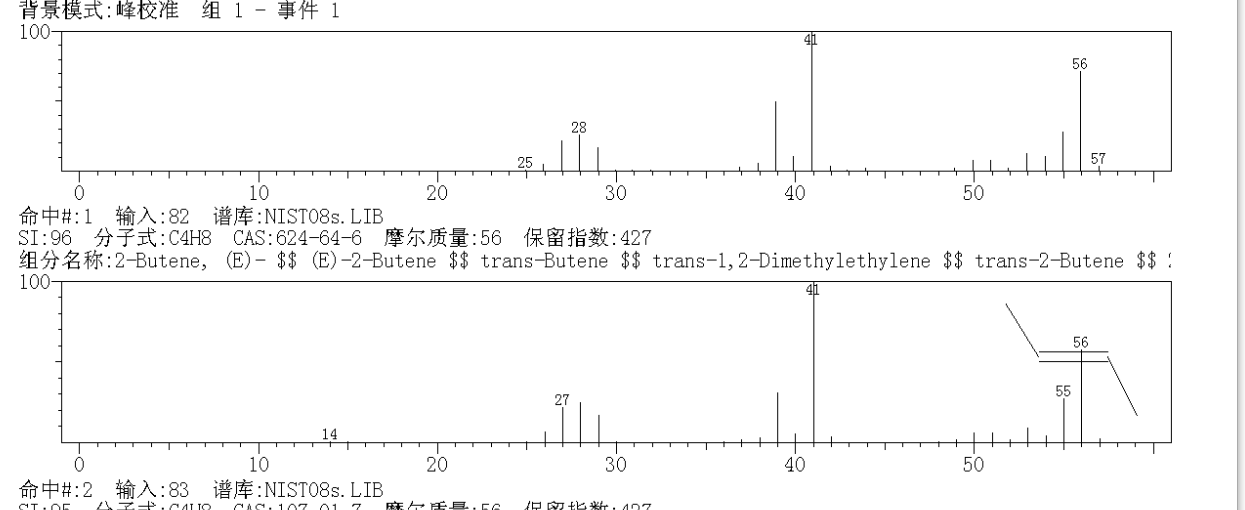


(a) MS spectra of the product


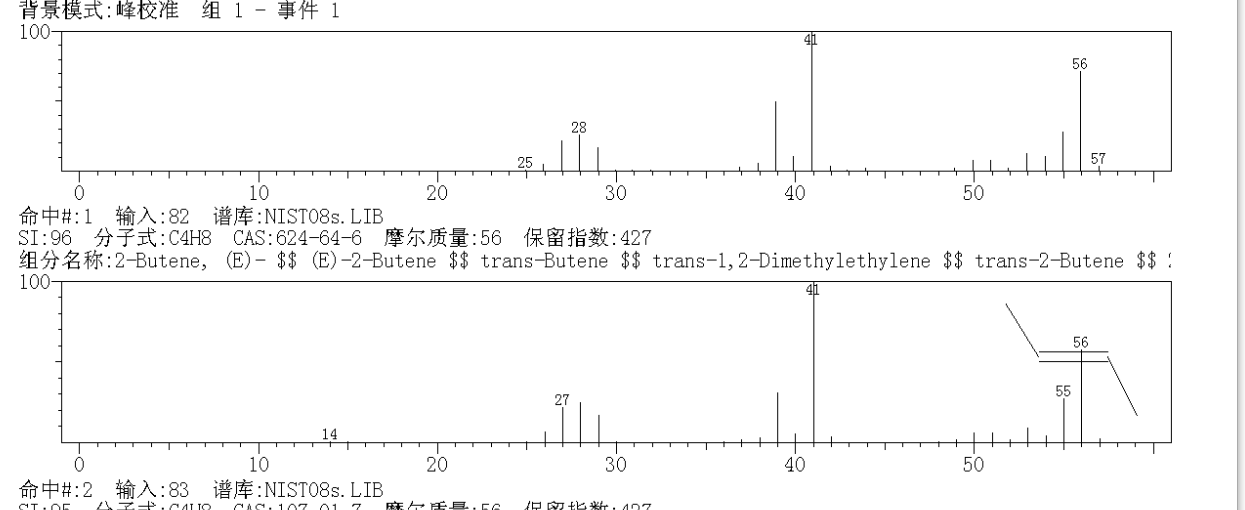


(b) MS spectra of the 2-butene

**Figure S39.** The mass spectra of the gas products of isoprene after the explosion. (2-Butene)


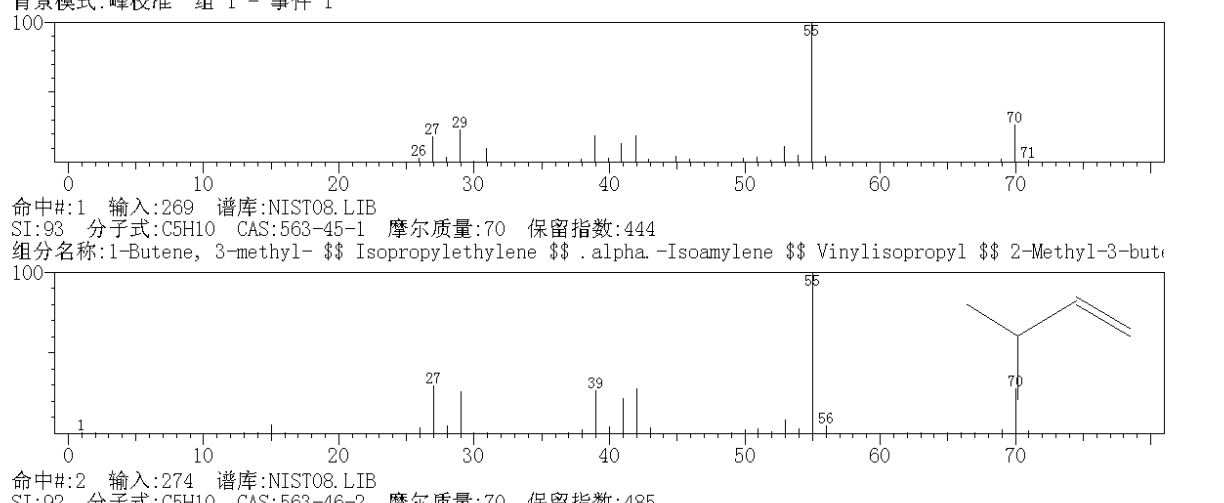


(a) MS spectra of the product


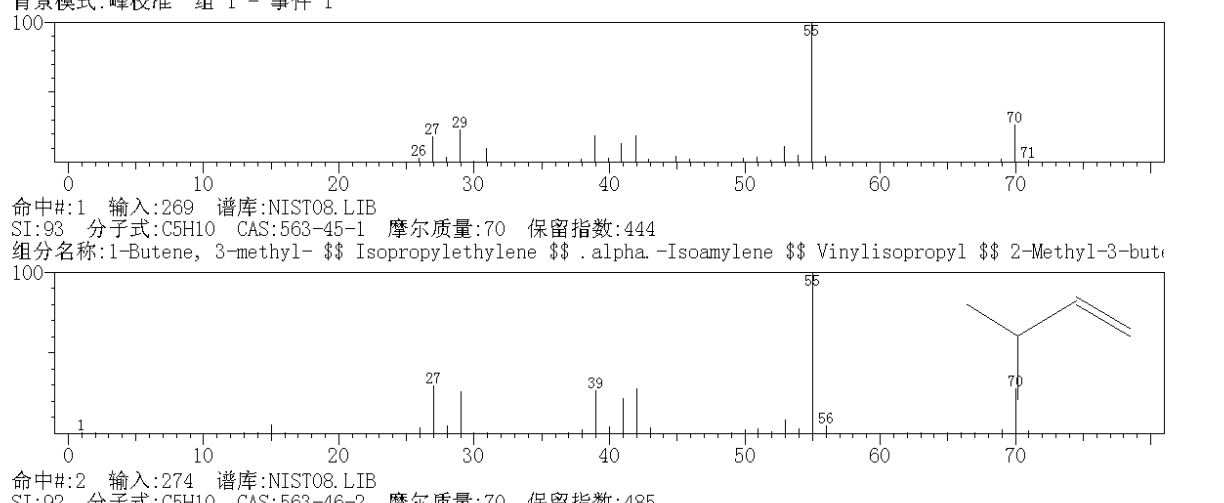


(b) MS spectra of the 3-methyl-1-butene

**Figure S40.** The mass spectra of the gas products of isoprene after the explosion. (3-Methyl-1-butene)


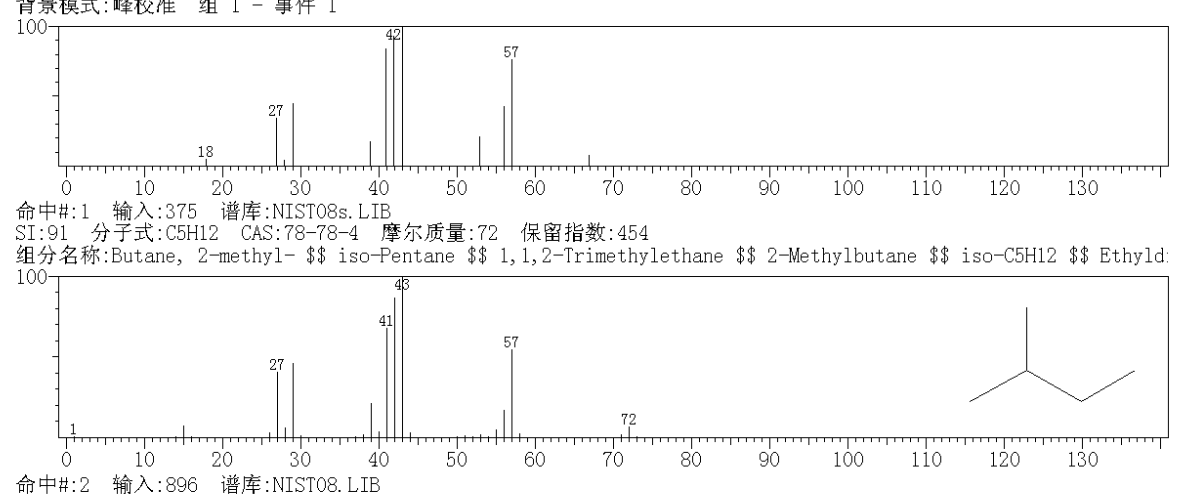


(a) MS spectra of the product


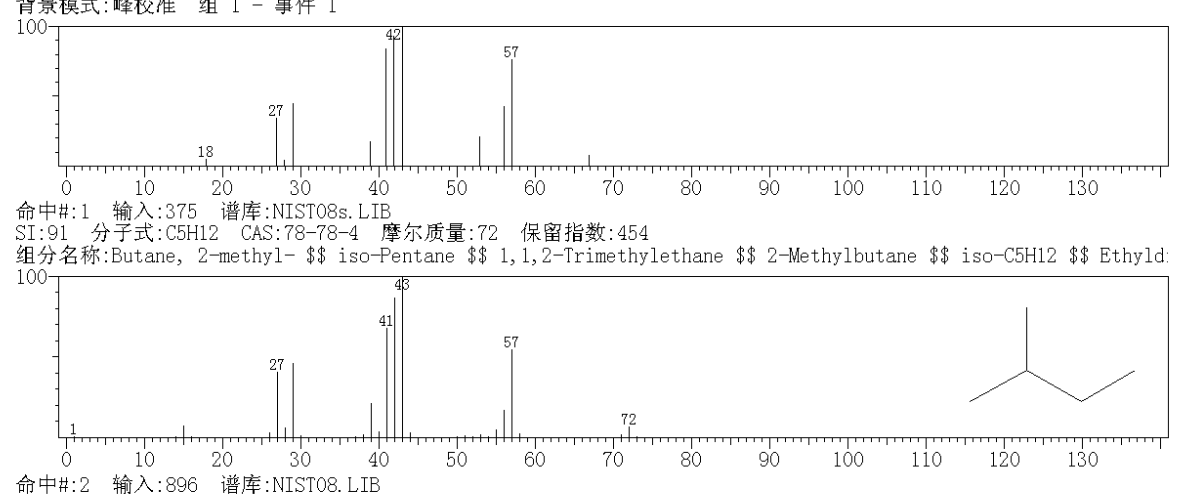


(b) MS spectra of the isopentane

**Figure S41.** The mass spectra of the gas products of isoprene after the explosion. (Isopentane)


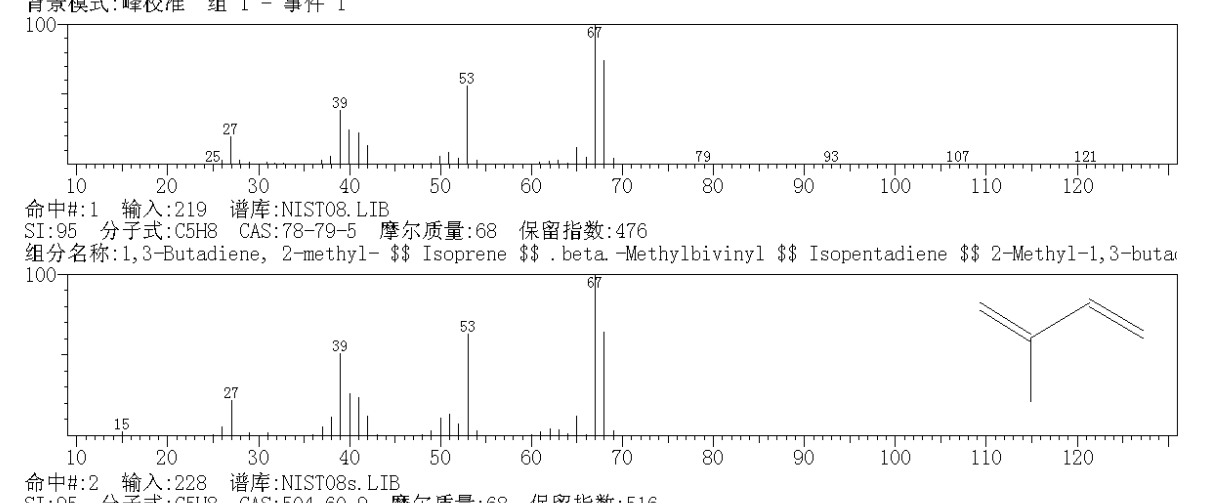


(a) MS spectra of the product


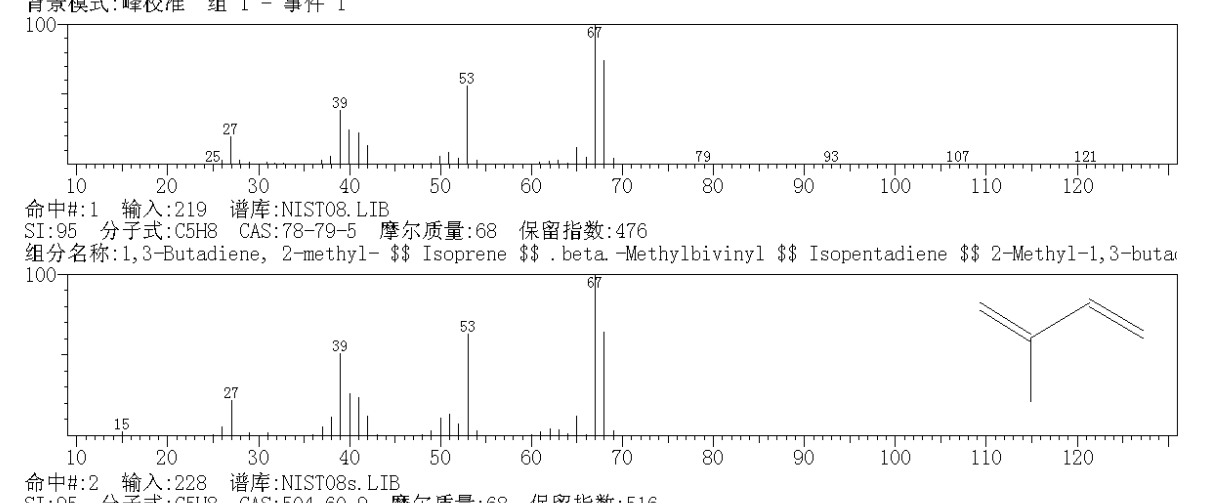


(b) MS spectra of the isoprene

**Figure S42.** The mass spectra of the gas products of isoprene after the explosion. (Isoprene)


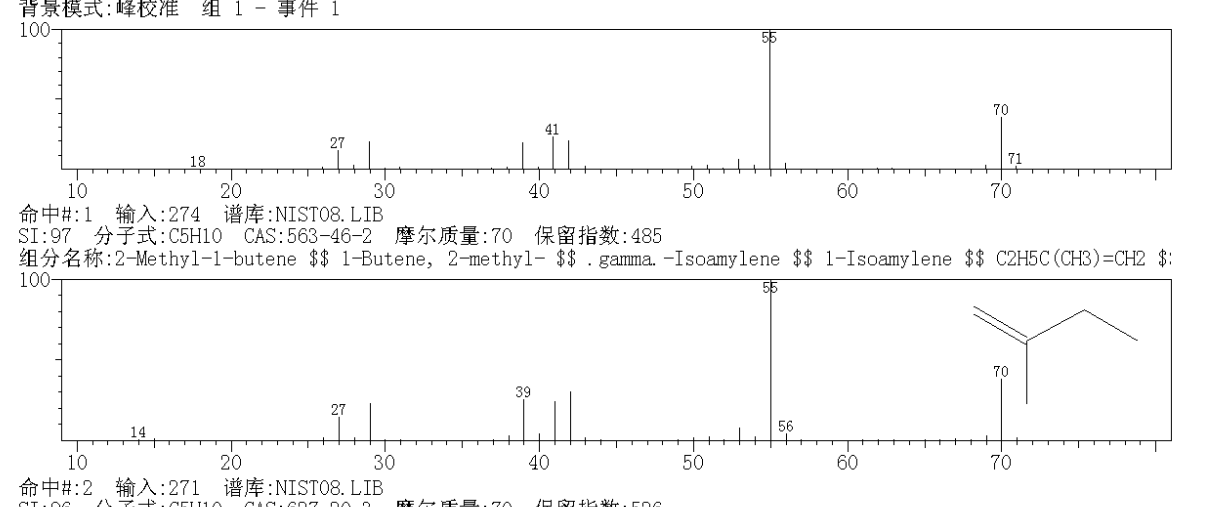


(a) MS spectra of the product


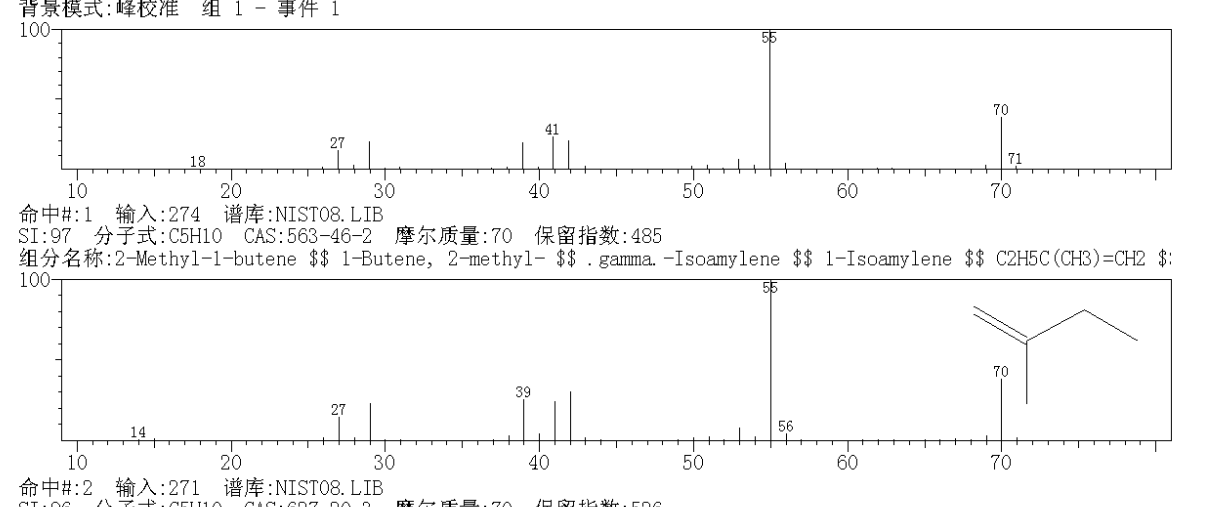


(b) MS spectra of the 2-methyl-1-butene

**Figure S43.** The mass spectra of the gas products of isoprene after the explosion. (2-Methyl-1-butene)


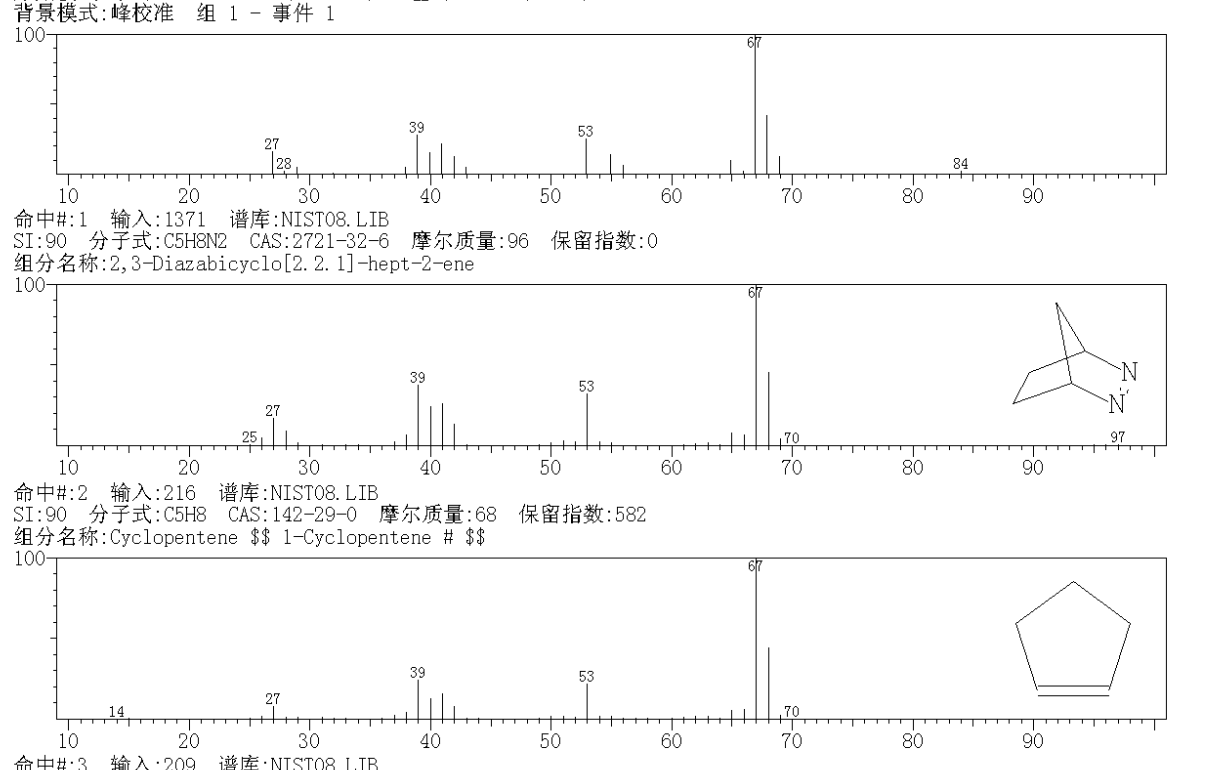


(a) MS spectra of the product


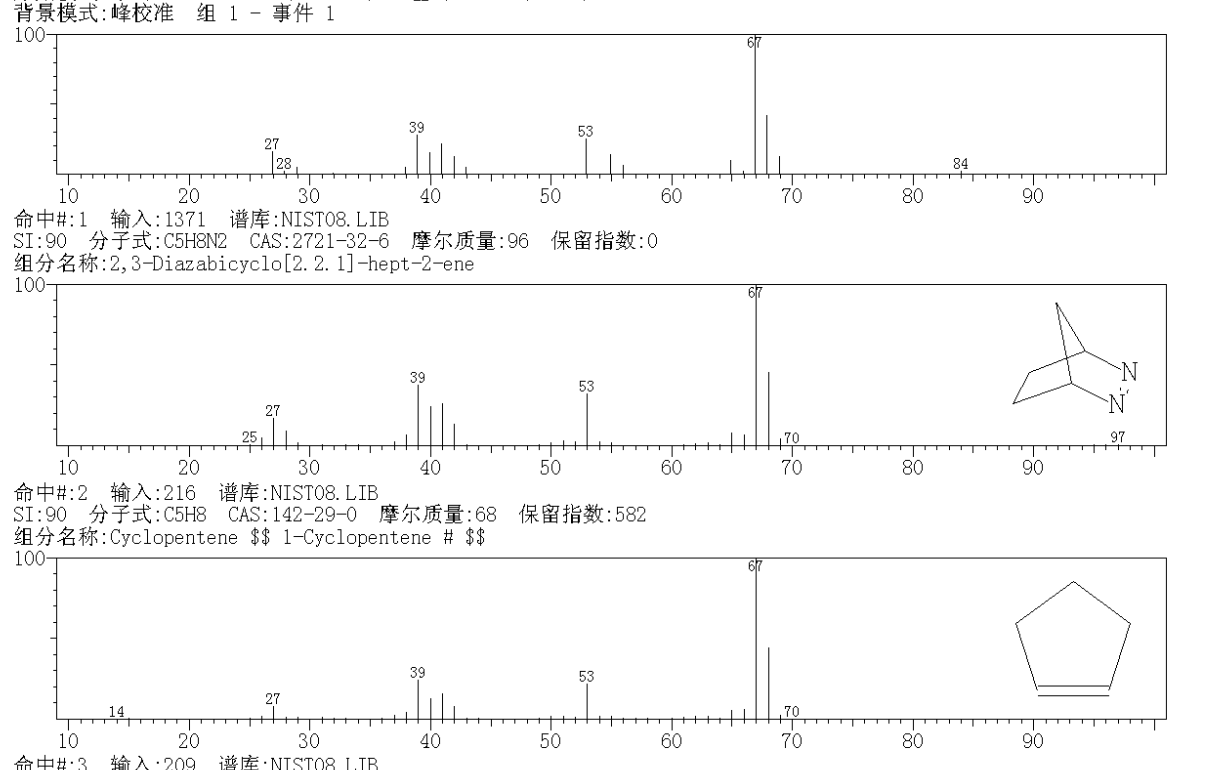


(b) MS spectra of the Cyclopentene

**Figure S44.** The mass spectra of the gas products of isoprene after the explosion. (Cyclopentene)


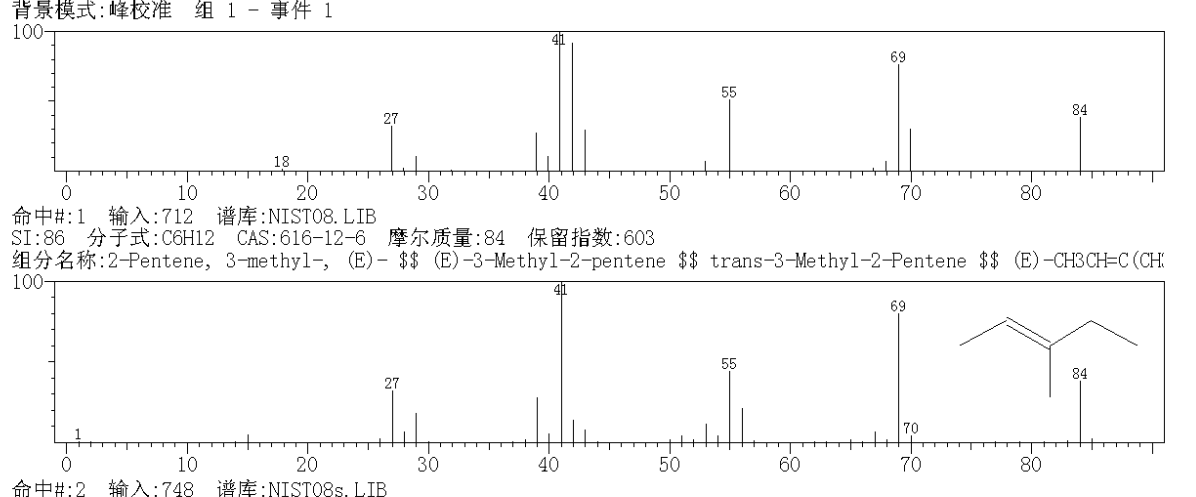


(a) MS spectra of the product


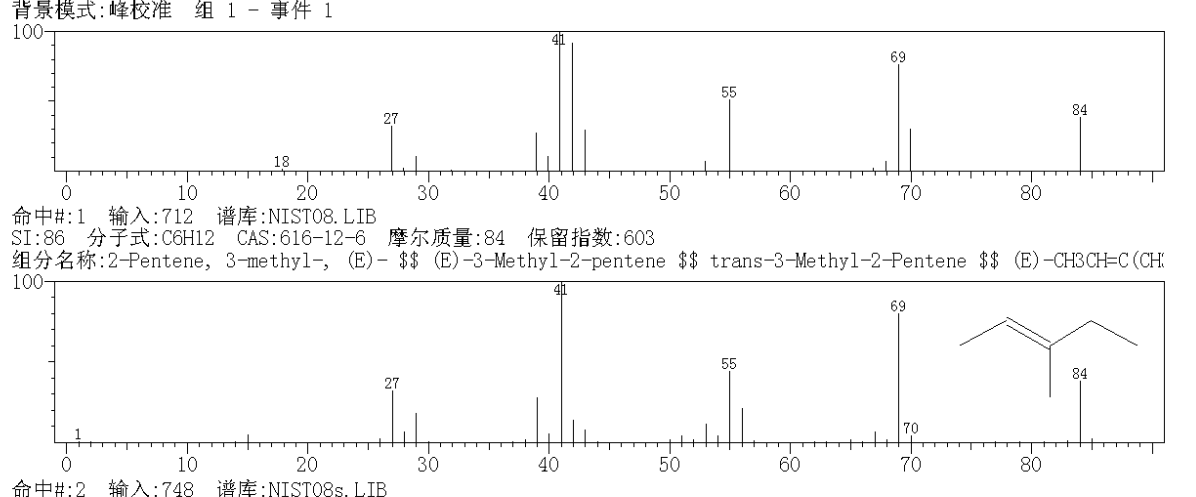


(b) MS spectra of the 3-methyl-2-pentene

**Figure S45.** The mass spectra of the gas products of isoprene after the explosion. (3-Methyl-2-pentene)


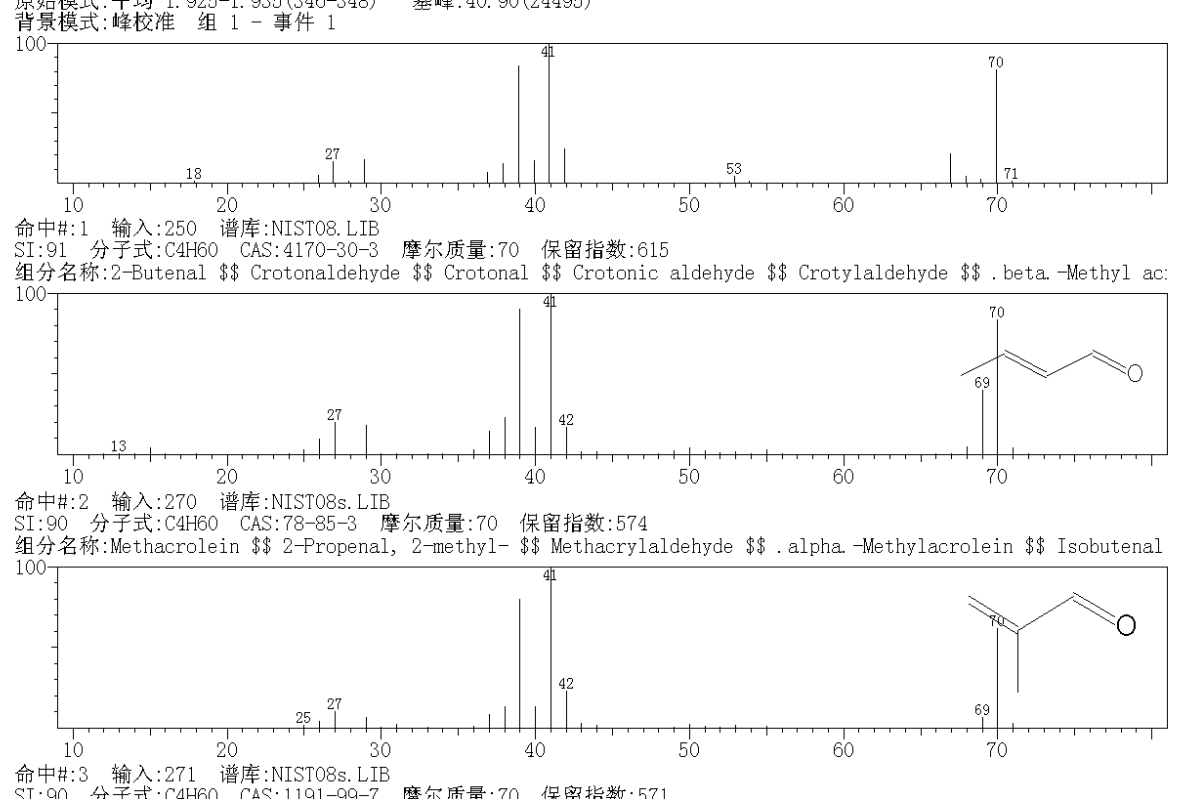


(a) MS spectra of the product


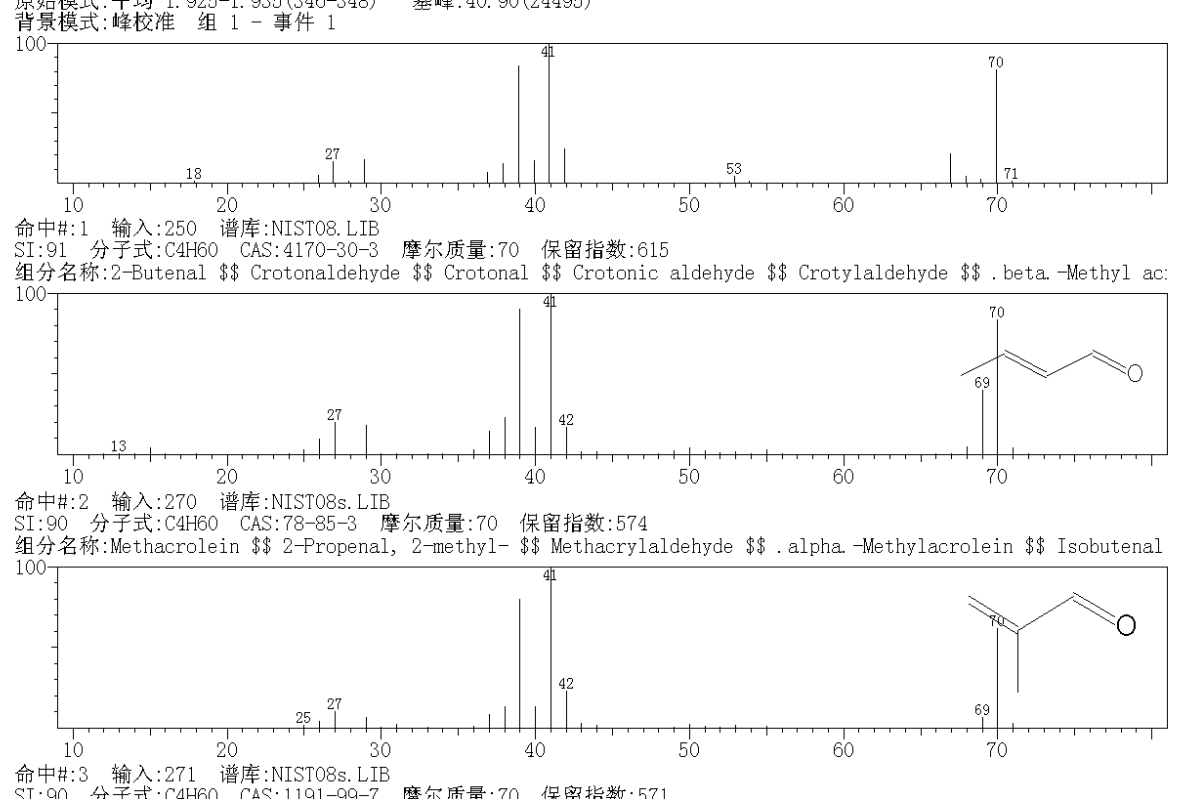


(b) MS spectra of the methacrolein

**Figure S46.** The mass spectra of the gas products of isoprene after the explosion. (Methacrolein)


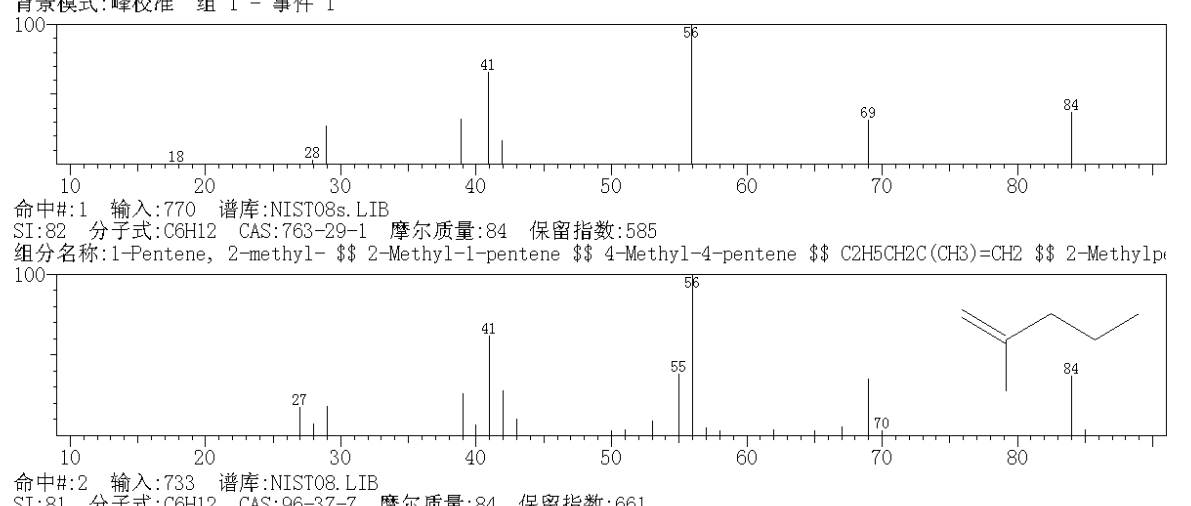


(a) MS spectra of the product


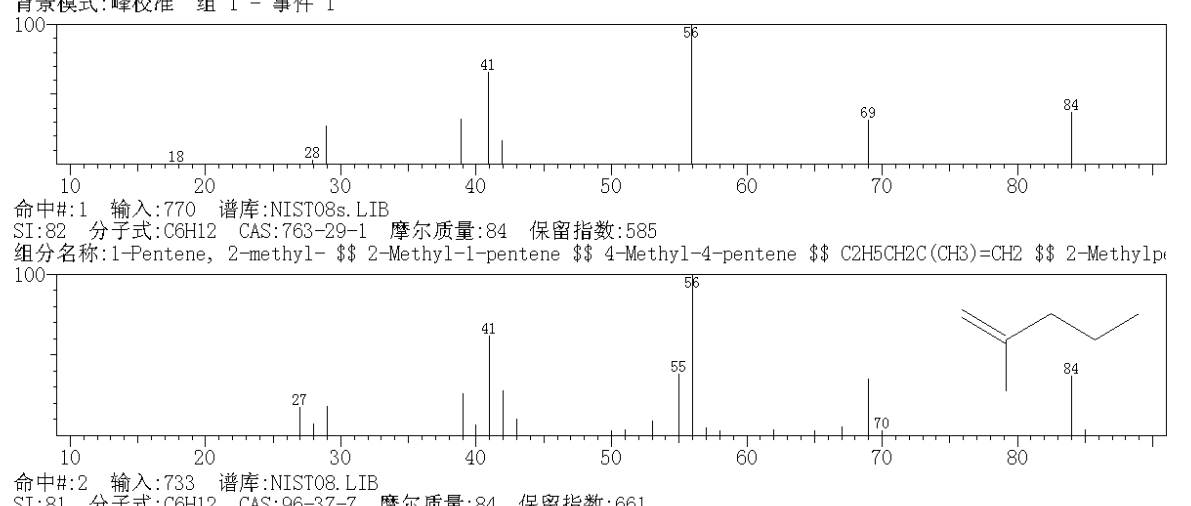


(b) MS spectra of the 2-methyl-1-pentene

**Figure S47.** The mass spectra of the gas products of isoprene after the explosion. (2-Methyl-1-pentene)


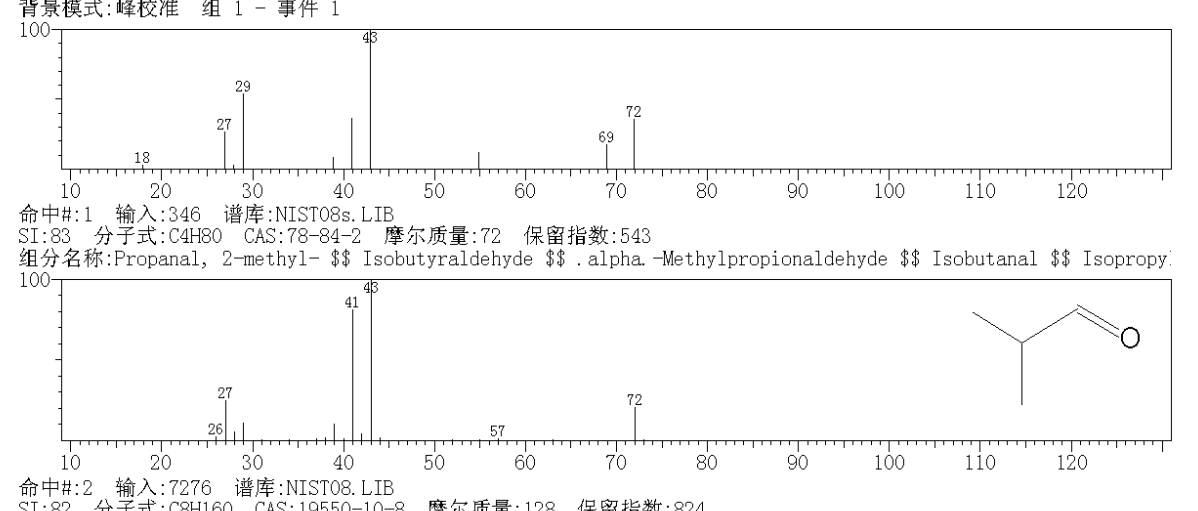


(a) MS spectra of the product


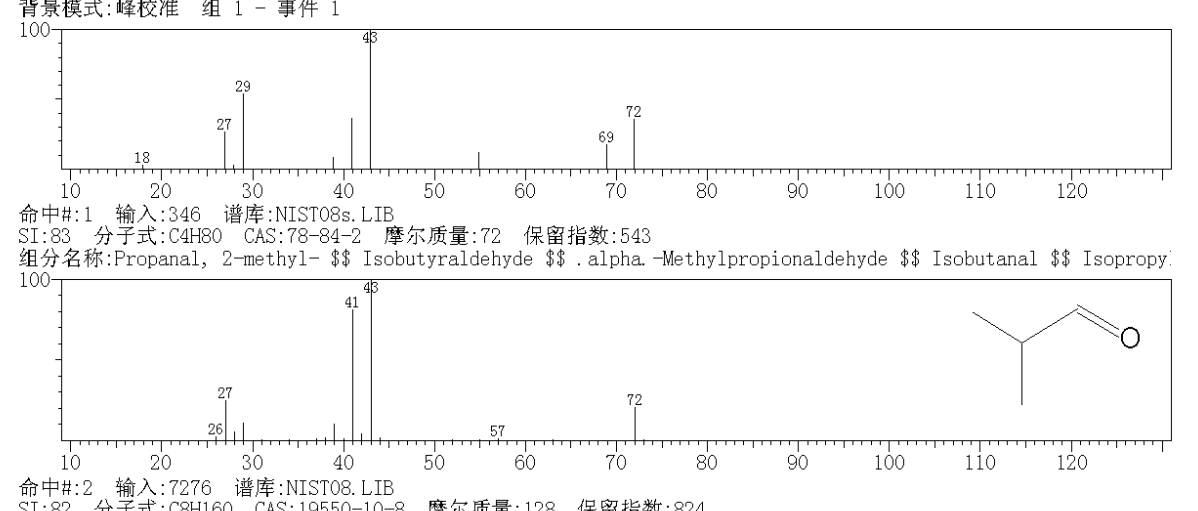


(b) MS spectra of the isobutyraldehyde

**Figure S48.** The mass spectra of the gas products of isoprene after the explosion. (Isobutyraldehyde)


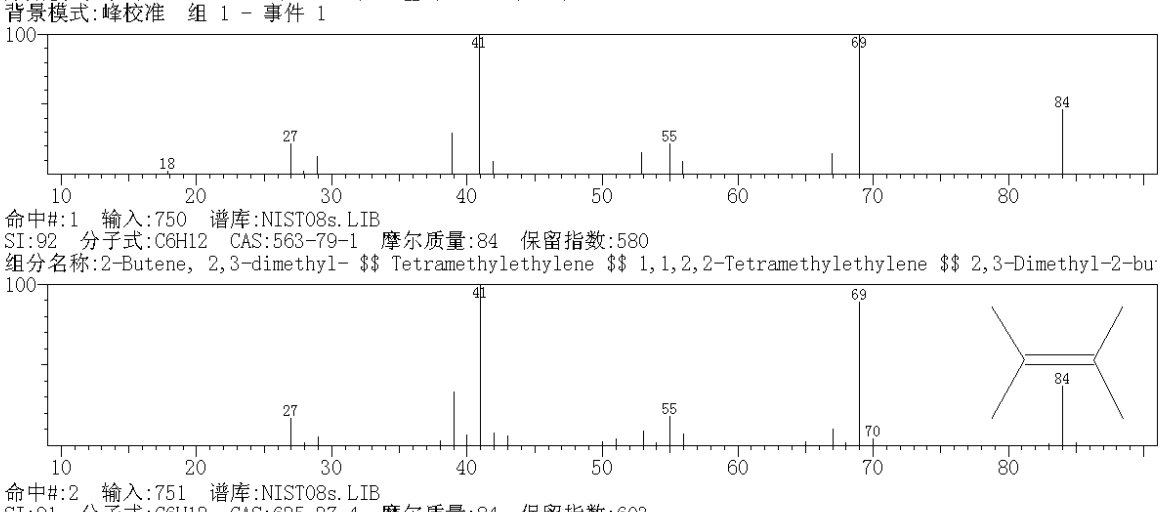


(a) MS spectra of the product


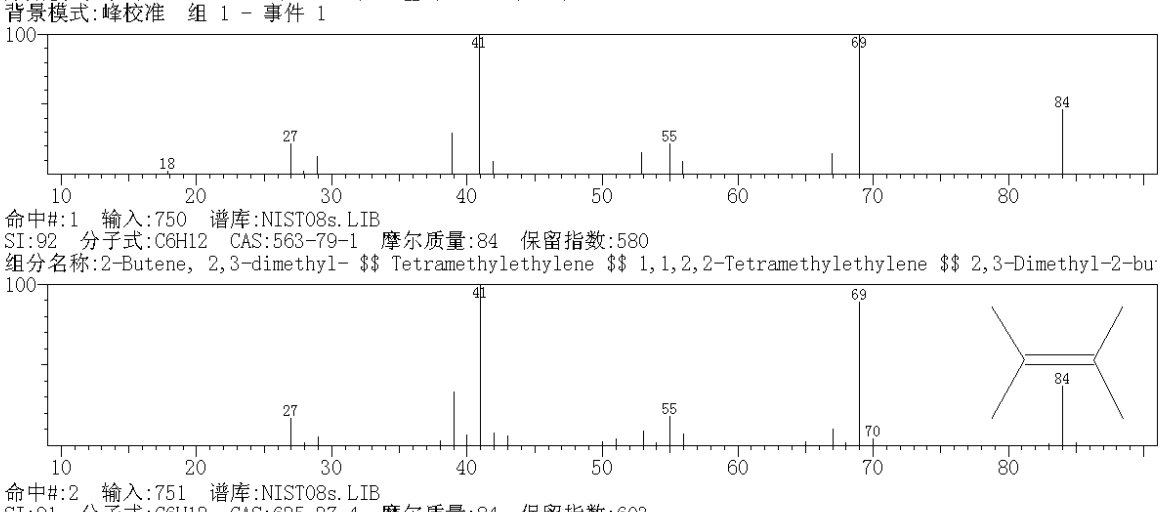


(b) MS spectra of the 2,3-Dimethyl-2-butene

**Figure S49.** The mass spectra of the gas products of isoprene after the explosion. (2,3-Dimethyl-2-butene)


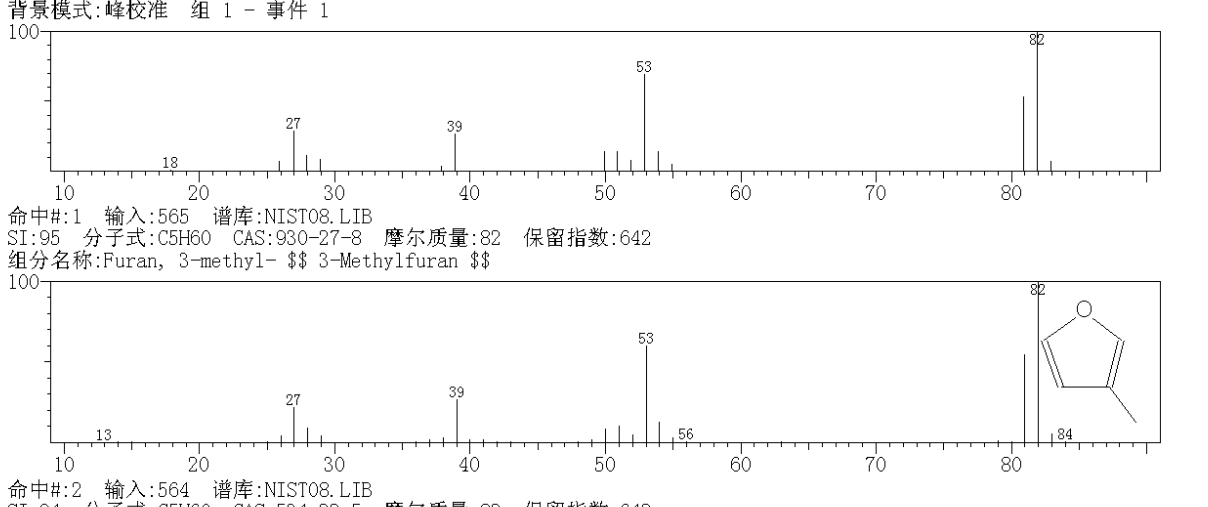


(a) MS spectra of the product


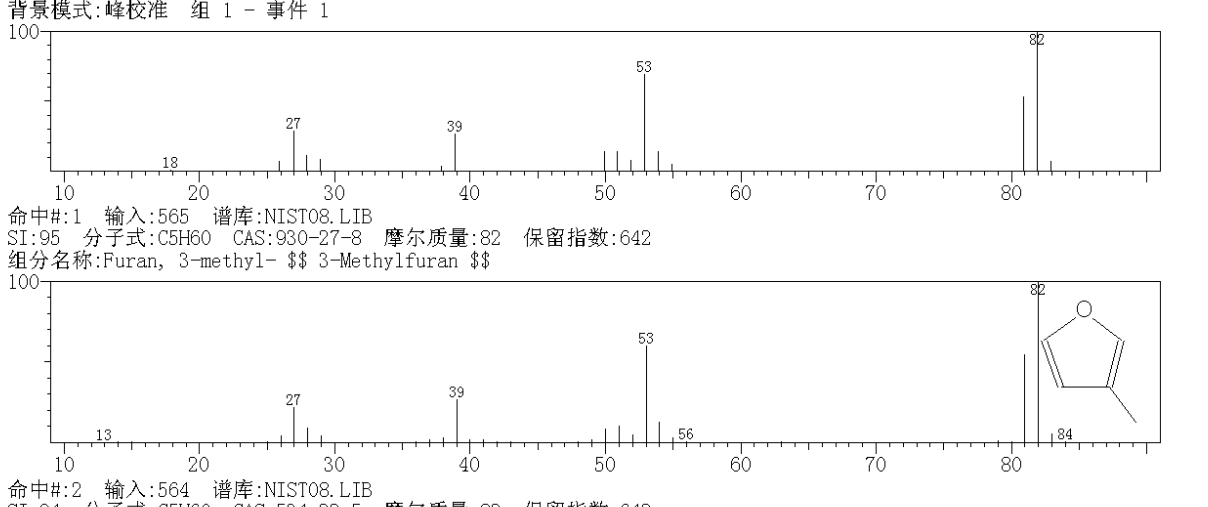


(b) MS spectra of the 3-methylfuran

**Figure S50.** The mass spectra of the gas products of isoprene after the explosion. (3-Methylfuran)


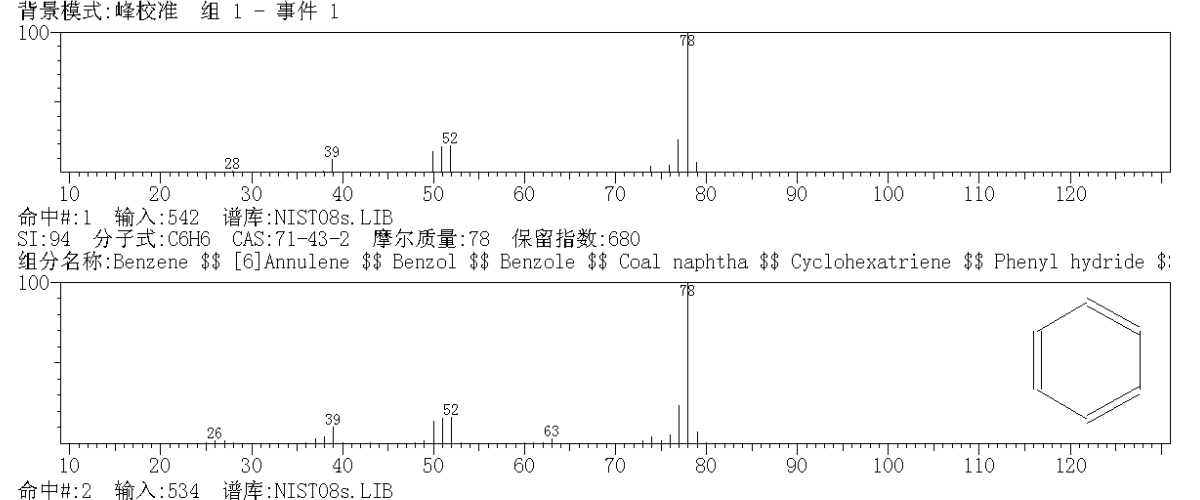


(a) MS spectra of the product


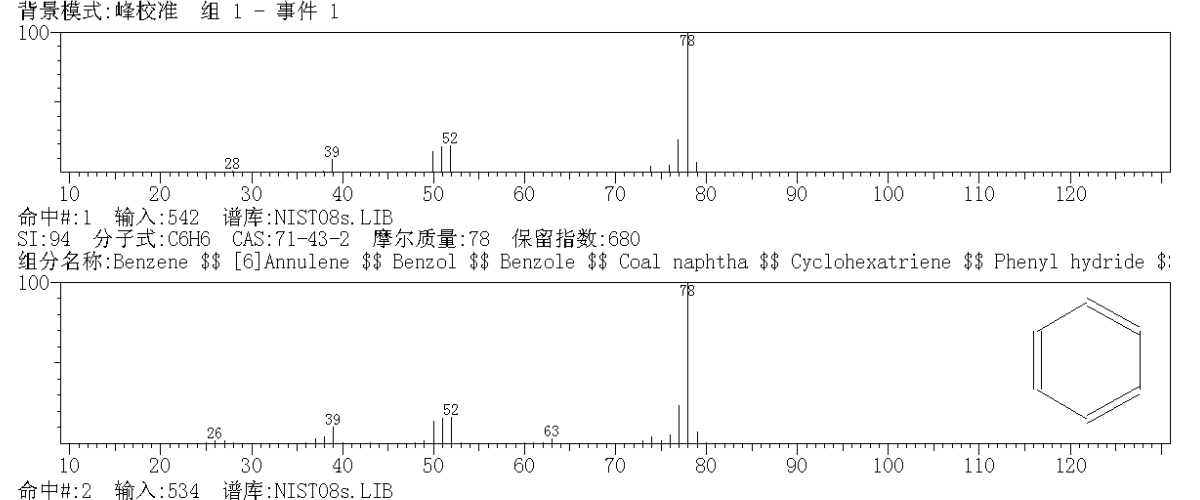


(b) MS spectra of the benzene

**Figure S51.** The mass spectra of the gas products of isoprene after the explosion. (Benzene)


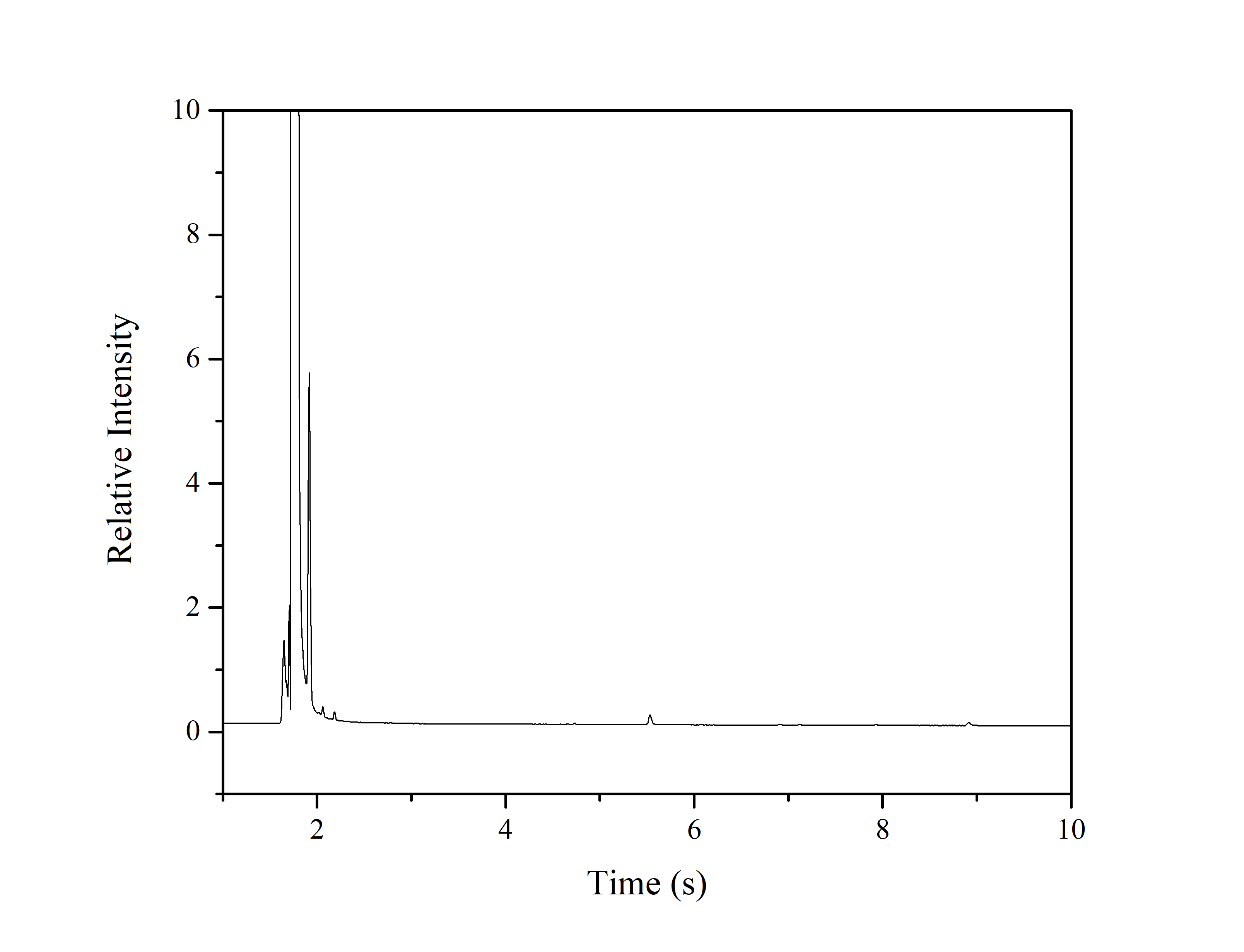


**Figure S52.** The Total ion chromatography of the liquid products of isoprene after the explosion.

(a) MS spectra of the product

(b) MS spectra of the dimethoxymethane

**Figure S53.** The mass spectra of the liquid products of isoprene after the explosion. (Dimethoxymethane)

(a) MS spectra of the product

(b) MS spectra of the 2,3-pentanedione

**Figure S54.** The mass spectra of the liquid products of isoprene after the explosion. (2,3-Pentanedione)

(a) MS spectra of the product

(b) MS spectra of the butyl acetate

**Figure S55.** The mass spectra of the liquid products of isoprene after the explosion. (Butyl acetate)

(a) MS spectra of the product

(b) MS spectra of the naphthalene

**Figure S56.** The mass spectra of the liquid products of isoprene after the explosion. (Naphthalene)

(a) MS spectra of the product

(b) MS spectra of the acenaphthylene

**Figure S57.** The mass spectra of the liquid products of isoprene after the explosion. (Acenaphthylene)

(a) MS spectra of the product

(b) MS spectra of the 9-methylene-9H-fluorene

**Figure S58.** The mass spectra of the liquid products of isoprene after the explosion. (9-Methylene-9H-fluorene)
